# Transcriptomic signature of frontotemporal lobar degeneration with TDP-43 type C pathology

**DOI:** 10.1093/brain/awag085

**Published:** 2026-03-06

**Authors:** Ana Rajicic, Merel O Mol, Shamiram Melhem, Helena Kisic, John C van Swieten, Harro Seelaar, Jeroen G J van Rooij

**Affiliations:** Department of Neurology and Alzheimer Center Erasmus MC, Erasmus University Medical Center, Rotterdam 3015 GD, The Netherlands; Department of Clinical Genetics, Erasmus University Medical Center, Rotterdam 3015 GD, The Netherlands; Department of Neurology and Alzheimer Center Erasmus MC, Erasmus University Medical Center, Rotterdam 3015 GD, The Netherlands; Department of Neurology and Alzheimer Center Erasmus MC, Erasmus University Medical Center, Rotterdam 3015 GD, The Netherlands; Department of Neurology and Alzheimer Center Erasmus MC, Erasmus University Medical Center, Rotterdam 3015 GD, The Netherlands; Department of Neurology and Alzheimer Center Erasmus MC, Erasmus University Medical Center, Rotterdam 3015 GD, The Netherlands; Department of Internal Medicine, Erasmus University Medical Center, Rotterdam 3015 GD, The Netherlands

**Keywords:** gene expression, dementia, frontotemporal dementia, semantic dementia

## Abstract

The semantic variant of primary progressive aphasia is a clinical subtype of frontotemporal lobar degeneration and is marked by TDP-43 subtype C pathology (FTLD-TDP C). It is a sporadic disease, yet has a strikingly homogeneous clinicopathological presentation, suggesting a common pathophysiology. The aim of this study was to discover dysregulated pathways in FTLD-TDP C through transcriptomics of the temporal cortex—its most affected region.

Bulk RNA sequencing was conducted on temporal cortices of a post-mortem cohort of 18 FTLD-TDP C patients and 23 sex- and age-matched controls. Differential expression and functional analyses were performed to identify differentially expressed genes [DEGs; false discovery rate (FDR) < 0.05] and functionally annotate them. We assessed the enrichment of *TARDBP*’s protein interactors and RNA targets among the DEGs. Our findings were compared with other published RNA sequencing data on tauopathies (Alzheimer’s dementia, progressive supranuclear palsy and FTLD with *MAPT*), FTLD-TDP (subtypes A and B) and available proteomics from this cohort. Additionally, we performed a weighted gene co-expression network analysis (WGCNA), adjusting for differences in cell type composition between cases and controls using cell deconvolution, and removing genes dysregulated in temporal cortices identified in other datasets. Among the FTLD-TDP DEGs, we focused on the enrichment of synaptic processes using SynGO.

We found upregulation of (i) damage response, (ii) cell structure and (iii) RNA splicing processes, and (iv) downregulation of synaptic processes in 6322 DEGs and five disease-related WGCNA modules. *TARDBP*-related genes were enriched among the DEGs. Additionally, transmembrane transport across the neurovascular unit was dysregulated. After cell deconvolution and removal of common tau genes, postsynaptic processes remained dysregulated, specifically the gene ontology terms ‘modulation of chemical synaptic transmission’ and ‘neurotransmitter receptor localization to postsynaptic specialization membrane’. We found 11 synaptic FTLD-TDP C-specific genes affected at both the RNA and protein levels in the temporal cortex, which were involved in synaptic adhesion (*CADM1*, *NCAN*), signal transmission (*COMT*, *RGS144*, *SLC1A2*, *TUBB2B*) and synaptic plasticity (*BEGAIN*, *ITPKA*, *LRFN1*, *RAB3B*, *SYNPO*).

In conclusion, a wide range of processes were dysregulated at the RNA level in the temporal cortex of FTLD-TDP C, including commonly affected processes in neurodegeneration, such as structural cell alterations. Dysregulation of *TARDBP*-related genes and RNA splicing has also been observed in other TDP-43 proteinopathies. Importantly, we found that postsynaptic processes were downregulated in FTLD-TDP C, after removing tauopathy-related genes and after cell deconvolution. In particular, the assembly of receptors at the postsynaptic membrane and synaptic signal transmission were affected, both at the RNA and protein levels. Future research on these pathways could elucidate distinct pathophysiological mechanisms and guide targeted clinical approaches.

## Teaser

Rajicic *et al.* performed bulk RNA sequencing on post-mortem temporal cortex from patients with FTLD-TDP C. After excluding tauopathy-related genes and adjusting for neuronal loss, they found a specific downregulation of postsynaptic pathways—particularly those involved in receptor assembly and synaptic transmission.

## Introduction

Frontotemporal lobar degeneration (FTLD) is a heterogeneous neurodegenerative disease with an early onset and varying clinical phenotypes, ranging from behavioural changes to language deficits, and from motor neuron disease to parkinsonism. The semantic variant of primary progressive aphasia (svPPA) is a clinical subtype of FTLD. It is a sporadic disease without any known underlying germline mutations, yet it has a strikingly consistent clinicopathological presentation. Clinically, it is characterized by impaired comprehension of language with initially preserved fluency of speech^1^ and behavioural changes, such as stereotypical behaviours and loss of empathy,^2^ which lead to a significant burden for patients and their caregivers. Neuroimaging shows asymmetrical temporal lobe atrophy, which affects the left side more than the right and spreads across other brain regions as the disease progresses.^3–5^ Pathologically, svPPA is most commonly marked by the accumulation of TAR DNA binding protein 43 (TDP-43)^6^ and is subtyped as FTLD-TDP subtype C (FTLD-TDP C).^7^ Herein, TDP-43 pathology is predominantly found in the temporal cortex in the form of dystrophic neurites and in the dentate gyrus of the hippocampus as compact, round neuronal cytoplasmic inclusions.^7^ Recently, annexin A11 (ANXA11) was discovered to aggregate together with TDP-43 in FTLD-TDP C,^8,9^ forming a new hallmark of the disease. The consistency in disease phenotype, despite the lack of a genetic cause, suggests that this disease might result from the disruption of a common pathway. Currently, little is known about the disease-causing mechanisms, and no targeted treatments exist.

In recent years, omics studies of brain tissue have provided more insights into the disease processes in FTLD, improving our pathophysiological understanding. For genetic FTLD-TDP subtypes A and B, transcriptomics studies on brain tissue have pointed towards blood–brain barrier disruption in progranulin carriers,^10^ whilst in *C9orf72* carriers, alterations in neuronal protein pathways and astrocytic activation and remodelling pathways have been described.^11^ For FTLD-TDP C, our whole exome sequencing study on brain tissue uncovered somatic pathogenic variants in the *TARDBP* gene, coding for TDP-43, in affected brain regions of 2 of 16 studied FTLD-TDP C brains.^12^ Given that *TARDBP* germline variants have been implicated in FTLD^13,14^ and amyotrophic lateral sclerosis (ALS),^15,16^ there may be a potential common molecular pathway wherein different disruptions could lead to distinct clinical phenotypes. In addition, our group has conducted proteomics studies on the dentate gyrus and temporal cortex of the same cohort of FTLD-TDP C patients, showing an increased expression of cell adhesion proteins (the catenin–cadherin complex at the synaptic membrane: β-catenin, γ-catenin and N-cadherin) in the dentate gyrus,^17^ plus disruption in mRNA metabolism and synaptic calcium regulation in the temporal cortex.^18^

Here, we performed the first transcriptomics study on FTLD-TDP C to comprehensively study gene expression in FTLD-TDP C and bridge our previous work at the DNA and protein levels. We conducted bulk RNA sequencing of the temporal cortex—the most affected brain region in FTLD-TDP C—and compared our findings with bulk RNA sequencing data from other neurodegenerative diseases, such as Alzheimer’s disease (AD) and other FTLD subtypes (A and B), to identify distinctly dysregulated pathways. Additionally, our transcriptomic findings were compared to temporal proteomics data on this cohort. We aimed to discover dysregulated pathways in FTLD-TDP C to generate pathophysiological hypotheses that, in future, could enable the identification of biomarkers and potential therapeutic targets specific for svPPA with underlying FTLD-TDP C.

## Materials and methods

### Post-mortem cohort

A post-mortem cohort of 18 FTLD-TDP C cases, clinically diagnosed with svPPA at the Erasmus University Medical Center (EMC), was included. All cases tested negative for pathogenic germline variants in major FTLD-related genes (*MAPT*, *GRN*, *C9orf72*, *TARDBP*, *TBK1*, *OPTN*, *SQSTM1*, *VCP*, *CHMP2B*, *FUS*, *ANXA11*) using whole exome sequencing in 16/18 and whole genome sequencing in 2/18. One case had a somatic variant in *TARDBP* in the medial temporal gyrus (R42H, #30) and another case in the dentate gyrus (L41F, #18) as previously described.^12^ In addition, 23 age- and sex-matched non-demented controls (NDC) were included from the brain donor registry of the Netherlands Brain Bank (NBB). Demographic, clinical and pathological characteristics were collected from medical records and autopsy reports. Informed consent was given by all patients and controls for the use of their brain tissue and clinical information for research. The protocol of the NBB is in accordance with the ethical standards of our institution, and with the 1964 Declaration of Helsinki and its later amendments or similar ethical standards, and has been approved by the ethics committee of the Amsterdam University Medical Center, location VUmc. Approval from an additional internal ethical committee was not required for this study, and all study procedures were performed in accordance with local ethical regulations. The workflow for further steps of the analyses is visualized in Supplementary Fig. 1.

### Brain tissue

Autopsies were performed by the NBB using standardized procedures and according to the Legal and Ethical Code of Conduct of the brain bank. For this study, fresh frozen brain tissue samples of the left temporal cortex (medial temporal gyrus preferred, and if unavailable, superior or inferior temporal gyrus) were acquired from the NBB, except for two cases from whom the temporal cortex of the right hemisphere was obtained (Supplementary Table 1). Neuropathological diagnoses described in autopsy reports were established by expert neuropathologists in keeping with current criteria^19–22^ on formalin-fixed paraffin-embedded tissue of the right hemisphere. Tissue was immunohistochemically stained using well-characterized antibodies for tau, TDP-43, amyloid-β and α-synuclein. In addition, TDP-43-stained slides of frontal and temporal cortices were assessed by A.R. and H.K. to confirm pathological diagnoses.

### RNA sequencing and processing

Total RNA was isolated using the manufacturer’s protocol (Qiagen AllPrep RNA isolation, Cat No. 80224). RNA quality was determined by the Agilent Bioanalyzer platform following the standard protocol. Sequencing was performed after poly-A selection and TruSeq library prep at the Human Genomics facility at the EMC (www.glimdna.org) on a HiSeq2000 at 2 × 50 bp. Data were processed per sample using trim-o-matic (v0.33), STAR (v2.3.0),^23,24^ picard (v1.90) and fastQC (v0.11.3). Transcript quantification was performed using featureCounts (v1.4.3) against all 57 820 gene features in GENCODE (version date: 2013-12-05).^25,26^ Autosomal protein-coding genes were retained for analysis. R software (v4.4.1) was used for data analyses. Counts were normalized using the edgeR (v3.8.6) trimmed mean of *M*-values method to counts per million values, and all low abundance features were omitted (<1 counts per million in 75% of samples). Hierarchical cluster analysis (HCA) and principal component (PC) analyses were performed to identify potential sample outliers. No samples were excluded based on the quality analyses (Supplementary material).

### Other datasets

We sought available RNA sequencing datasets of the temporal cortex of dementias in PubMed and the NCBI GEO database (see Supplementary material for search query and acquisition). Raw gene count processing and quality control steps were similar to our FTLD-TDP C data. We used the available data on tauopathies [AD, progressive supranuclear palsy (PSP) and FTLD with *MAPT* 10 *+* 16 deletion (FTLD-MAPT)] to remove commonly affected genes (Supplementary material). Additionally, we compared the RNA sequencing outcomes to a FTLD-TDP dataset of the temporal cortex containing other subtypes (Supplementary material) and to the proteomics dataset of the temporal cortex of this post-mortem cohort.^18^

### Differential expression analysis

Cohort characteristics were assessed using appropriate descriptive statistics (Supplementary material). We identified differentially expressed genes between diseased samples and controls using linear regression, correcting for age, sex, batch and RNA integrity number (RIN). Genes with an FDR <0.05 using Benjamini–Hochberg were considered significantly differentially expressed genes (DEGs). To determine FTLD-TDP-related DEGs, we removed the following genes: (i) genes that were only detected in FTLD-TDP C, but not in tau datasets, to ensure inclusion of technically reliable genes; and (ii) DEGs that were also FDR-significant with the same fold-change direction in AD or FTLD with underlying tauopathy (FTLD-MAPT, PSP). To further identify FTLD-TDP C-specific genes, we removed overlapping genes with FTLD-TDP, i.e. those that were significant with the same fold-change direction. The ggVennDiagram package (v1.5.4) in R was used to visualize gene overlap.

### Assessing enrichment for *TARDBP*-related genes

As *TARDBP* may play a relevant role in the disease mechanisms underlying this TDP-43 proteinopathy, we assessed the enrichment of *TARDBP*’s RNA targets and protein interactors among the DEGs using the chi-square test. RNA targets of *TARDBP* were identified using the meta-analysis by Cao *et al.*^27^ and the more recent paper by Seddighi *et al.*,^28^ whilst protein interactors were identified using the meta-analysis by Helmold *et al.*,^29^ as well as the String database (v.12.0) using the following evidence settings: experimental interactions, co-expression, phylogenetic co-occurrence and medium confidence of 0.4 (Supplementary material).

### Cell deconvolution

To correct for potential cell composition bias in the differential expression of genes between FTLD-TDP C and controls, cell deconvolution was conducted on raw gene counts using CIBERSORTx^30^ in RStudio (permission granted by the CIBERSORTx team; Supplementary material). Gene counts determined by single-nucleus RNA sequencing of the medial temporal gyrus from the Allen Human Brain Atlas^31^ were used as a signature matrix. Differences in cell composition between FTLD-TDP C and controls were determined for seven cell types: neurons, endothelial cells, microglia, oligodendrocytes, oligodendrocyte precursor cells (OPCs), astrocytes (SLC14A1 positive) and reactive astrocytes (GFAP positive). A differential expression analysis was performed, correcting for differences in neurons and reactive astrocytes across samples, as these cell types contributed most to cell composition differences. Other covariables for which we corrected in this model were age at death, sex and RIN.

### Gene ontology enrichment analysis

Gene ontology enrichment analyses were conducted to determine which gene functions were most abundant among the DEGs in FTLD-TDP C. Significant terms with a gene count of at least five were included. Gene set enrichment analysis (GSEA) enables the detection of classes of genes that are overrepresented in a large set of genes, regardless of the significance level of genes, sorting by fold change.^32^ We ran GSEA for up- and downregulated genes separately using the gseGO() function of the clusterProfiler package (v4.12.2) in RStudio. Overrepresentation analysis (ORA) was performed to identify enriched pathways in the overlapping gene sets between FTLD-TDP C and the other acquired transcriptomics datasets of the temporal cortex: overlap between FTLD-TDP C and neurodegeneration (aggregating tau and FTLD-TDP findings) and overlap between FTLD-TDP C and FTLD-TDP. We also ran ORA on overlapping findings at the gene and protein levels^18^ in this cohort. To determine which functions were related to FTLD-TDP and FTLD-TDP C specifically, ORA was run on these subsets of genes after cell deconvolution. We ran all ORA for up- and downregulated genes separately using enrichGO() from clusterProfiler (v4.12.2) in RStudio, setting all detected genes in our FTLD-TDP C dataset as background. Top significant gene ontology terms (GO terms) for biological processes (BP) and cell components (CC) were visualized in dot plots and tree plots using the enrichplot package (v1.24.4) in RStudio. The redundancy of GO terms was manually curated by comparing the overlap in genes involved in each term. In addition, SynGO^33^ was used to annotate synapse-related genes, using standard settings, with FTLD-TDP-related DEGs as input and our detected genes as background.

### Gene network analysis

Genes annotated by SynGO were visualized in a gene network using stringApp (v2.2.0) in Cytoscape (v3.10.3). The following settings were applied: experimentally determined interactions, phylogenetic co-occurrence and co-expression, with a minimum confidence score of 0.1. Additionally, enriched synaptic structures and functions were visualized in the network. Furthermore, an overview was created of all the SynGO-annotated genes using information from GeneCards,^34^ the Human Protein Atlas^35,36^ and literature retrieved through PubMed by searching for the gene name.

### Co-expression analysis

To analyse the system-level functionality of genes, we ran module-level analyses using the weighted gene co-expression network analysis (WGCNA) package (v1.72-5) in R (Supplementary material). This analysis clusters all genes into modules, which represent groups of genes that are positively or negatively correlated. For interpretation, the modules were correlated to known phenotype characteristics, such as disease status, age at death, sex, RIN and post-mortem delay (PMD), and were functionally annotated using ORA.

FTLD-TDP C-related modules were determined by correlating the modules’ first PCs—termed module eigengenes—to disease status and other known phenotype characteristics. A circular heat map of these correlations was created using the circlize package (v0.4.16) in R. Additionally, modules were correlated to cell types. These correlations were visualized in a heat map using the pheatmap package (v.1.0.12) in R. To annotate the modules, ORA was conducted on each module using the enrichGO() function from the clusterProfiler package (v4.12.2) in R, by setting the module genes as input and total genes as background. Finally, the spread of DEGs across the modules was assessed, and the proportions of DEGs per module were calculated.

## Results

### Cohort description

Demographic, clinical and pathological characteristics of the 18 patients with a clinical diagnosis of svPPA and underlying FTLD-TDP C and 23 NDC are listed in Table 1. There were no significant differences in sex and age at death between cases and NDC, as expected given the sex- and age-matching of the NDC. Co-pathology in the temporal cortex was observed more frequently in NDC (83%) than in FTLD-TDP C (39%) but was not significantly different. It consisted mostly of mild Alzheimer pathology with a median ABC score of 2 [interquartile range (IQR) = 1–2] in NDC and 0 (IQR = 0–2) in FTLD-TDP C. Of the NDC with mild Alzheimer pathology, four additionally showed ageing-related tau astrogliopathy (ARTAG), one primary age-related tauopathy (PART), one signs of hypoxia and one signs of hypertension (more details in Supplementary Table 1). As expected, brain weights of FTLD-TDP C were significantly lower than those of NDC (mean = 971.1 ± 138.4 versus 1249.1 ± 183.1, *P* < 0.0001). Other baseline characteristics such as pH of tissue, PMD and RIN were not significantly different between cases and NDC.

**Table 1:** Demographic, clinical and pathological characteristics of the post-mortem cohort

	FTLD-TDP C (*n* = 18)	NDC (*n* = 23)	*P*-value^a^
Sex, female	0.56 (±0.50)	0.39 (±0.49)	0.465^b^
Age at death, years	68.6 (±3.8)	71.4 (±10.6)	0.251^c^
Age at onset, years	56.7 (±6.2)	NA	NA
Disease duration, years	11.9 (±3.8)	NA	NA
Mean no. of comorbidities per participant	1 (0–2)	2 (1–3)	0.005^d^
Brain weight, g	971.7 (±138.4)	1249.1 (±183.1)	**3.24 × 10^−6c^**
pH	6.48 (±0.32)	6.55 (±0.39)	0.533^c^
Post-mortem delay, min	325 (276–393)	430 (333–505)	0.014^d^
RIN	6.9 (5.7–7.5)	7.3 (7.0–7.8)	0.042^d^
Percentage of samples with co-pathology	39 (±49)	83 (±38)	0.008^e^
ABC score^f^	0 (0–2)	2 (1–2)	0.028^d^

Values are presented as mean (±standard deviation) or median (interquartile range).

FTLD-TDP C = frontotemporal lobar degeneration with TDP-43 subtype C; NA = not applicable; NDC = non-demented controls; RIN = RNA integrity number.

^a^Significant *P* < 0.005 (in bold) according to Bonferroni multiple-testing correction.

^b^Chi-square test.

^c^Independent samples *t*-test.

^d^Mann–Whitney U-test.

^e^Fisher’s exact test.

^f^ABC score for Alzheimer’s disease-related neuropathological changes consisting of amyloid-β deposits (A0–3), neurofibrillary tangles (B0–3) and neuritic plaques (C0–3), with a maximum score of 9.^20^ A score of ≥4 is considered to be potentially related to symptoms and/or dementia.

### Cell structure, immune response and synaptic processes disrupted in FTLD-TDP C

Comparison of gene expression in FTLD-TDP C versus NDC identified 6322 genes with FDR < 0.05, of which 3073 were up- and 3249 were downregulated (Fig. 1A and Supplementary Table 1). GSEA using ClusterProfiler found 517 enriched GO terms for up- and 322 enriched GO terms for downregulated DEGs (Supplementary Table 1). The top 30 significantly enriched terms for upregulated DEGs were processes related to immune response and structural cell components, namely ‘regulation of cytokine activation response’, ‘leukocyte migration’, ‘membrane receptor activity’, ‘collagen-containing matrix’ and ‘extracellular structure organization’ (Fig. 1B). Downregulated DEGs were enriched for terms related to neuronal structures and synapse activity, including: ‘glutamatergic synapse organization’, ‘dendrite’, ‘synapse specialization’, ‘chemical trans-synaptic signaling’ and ‘ion channel transport activity’ (Fig. 1C). Of note, *TARDBP* was one of the significantly upregulated genes in this TDP-43 proteinopathy (fold change = 0.25; FDR = 0.035), while ANXA11 was not (fold change = −0.11; FDR = 0.728). Focusing on *TARDBP*-related genes, we found that both RNA targets of *TARDBP* and *TARDBP*’s protein interactors were enriched in the significant genes, with 42% of the RNA targets (*P* = 0.003) and 39% of protein interactors (*P* = 0.025) being significant, compared to 27% of all genes (Supplementary Table 1).

**Figure 1:**
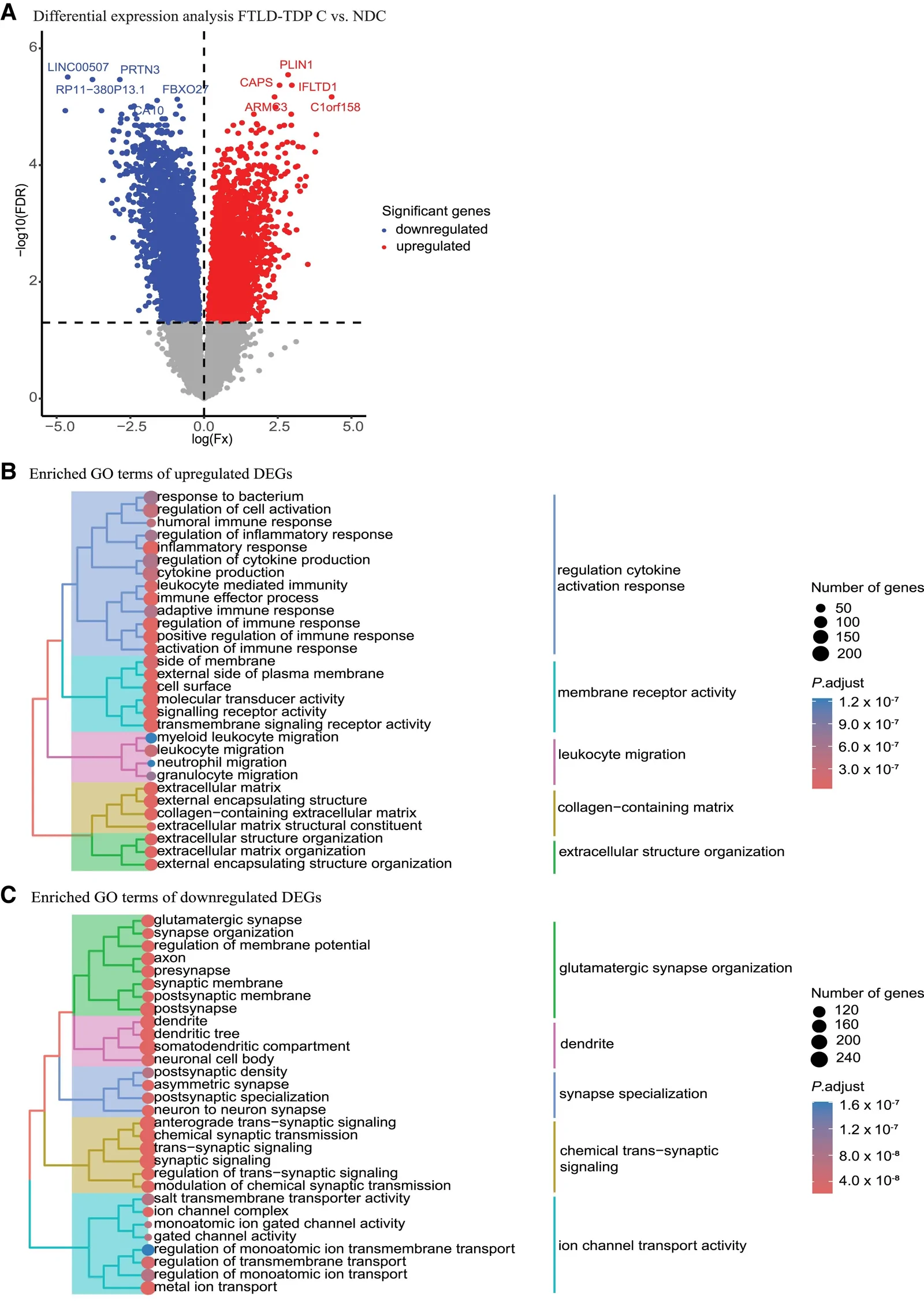
**Differentially expressed genes in the temporal cortex of FTLD-TDP C versus non-demented controls.** (**A**) The volcano plot shows differentially expressed genes (DEGs), depicting significantly upregulated genes in red and significantly downregulated genes in blue. Log_2_ fold changes are shown on the *x*-axis, while *P*-values corrected for multiple testing using Benjamin–Hochberg false discovery rate are shown on the *y*-axis. The tree plots depict significantly enriched gene ontology terms in significantly (**B**) upregulated and (**C**) downregulated genes. The size of each circle per term represents the number of genes related to this term, while the colour indicates the significance level, with red being more significant than blue. The terms are clustered hierarchically and grouped by colour according to an overlapping function. Additionally, a label is given to the grouping on the *right*. (**B**) Among the significantly upregulated genes, the top enriched gene ontology terms are: ‘regulation of cytokine activation response’, ‘leukocyte migration’ and structural cell components, such as ‘membrane receptor activity’, ‘collagen-containing matrix’ and ‘extracellular structure organization’. (**C**) Among significantly downregulated genes, the top enriched gene ontology terms are related to neuronal structures and synapses: ‘glutamatergic synapse organization’, ‘dendrite’, ‘synapse specialization’, ‘chemical trans-synaptic signaling’ and ‘ion channel transport activity’. DEGs = differentially expressed genes, FDR < 0.05; Fx = fold change; FDR = false discovery rate corrected *P*-value; FTLD-TDP C = frontotemporal lobar degeneration with underlying TDP-43 proteinopathy subtype C; GO terms = gene ontology terms; *P*.adjust = Benjamini–Hochberg corrected *P*-value.

### Overlap in transcriptomics of FTLD-TDP C with tau, TDP-proteinopathy and FTLD-TDP C proteomics

We assessed the overlap of differential gene expression in the temporal cortex between FTLD-TDP C, tauopathies (AD, PSP, FTLD-MAPT) and other FTLD-TDP subtypes (A and B). Of the 6322 DEGs in FTLD-TDP C in our cohort, 3619 (57%) were also significantly dysregulated in tauopathies or other FTLD-TDP (Fig. 2A), with 3142 (50%) showing the same fold-change direction. The top three overrepresented upregulated biological processes in these overlapping genes were ‘response to wounding’, ‘angiogenesis’ and ‘cytokine-mediated signaling pathway’, whereas the top downregulated biological processes were ‘modulation of chemical synaptic transmission’, ‘synapse organization’ and ‘regulation of membrane potential’ (Fig. 2B and Supplementary Table 2). Most overrepresented upregulated processes were related to damage response, immune response and alterations in cell structure, while downregulated processes involved synaptic function and structure, similar to the functional enrichment in the significant genes of this FTLD-TDP C cohort (Fig. 1B).

**Figure 2:**
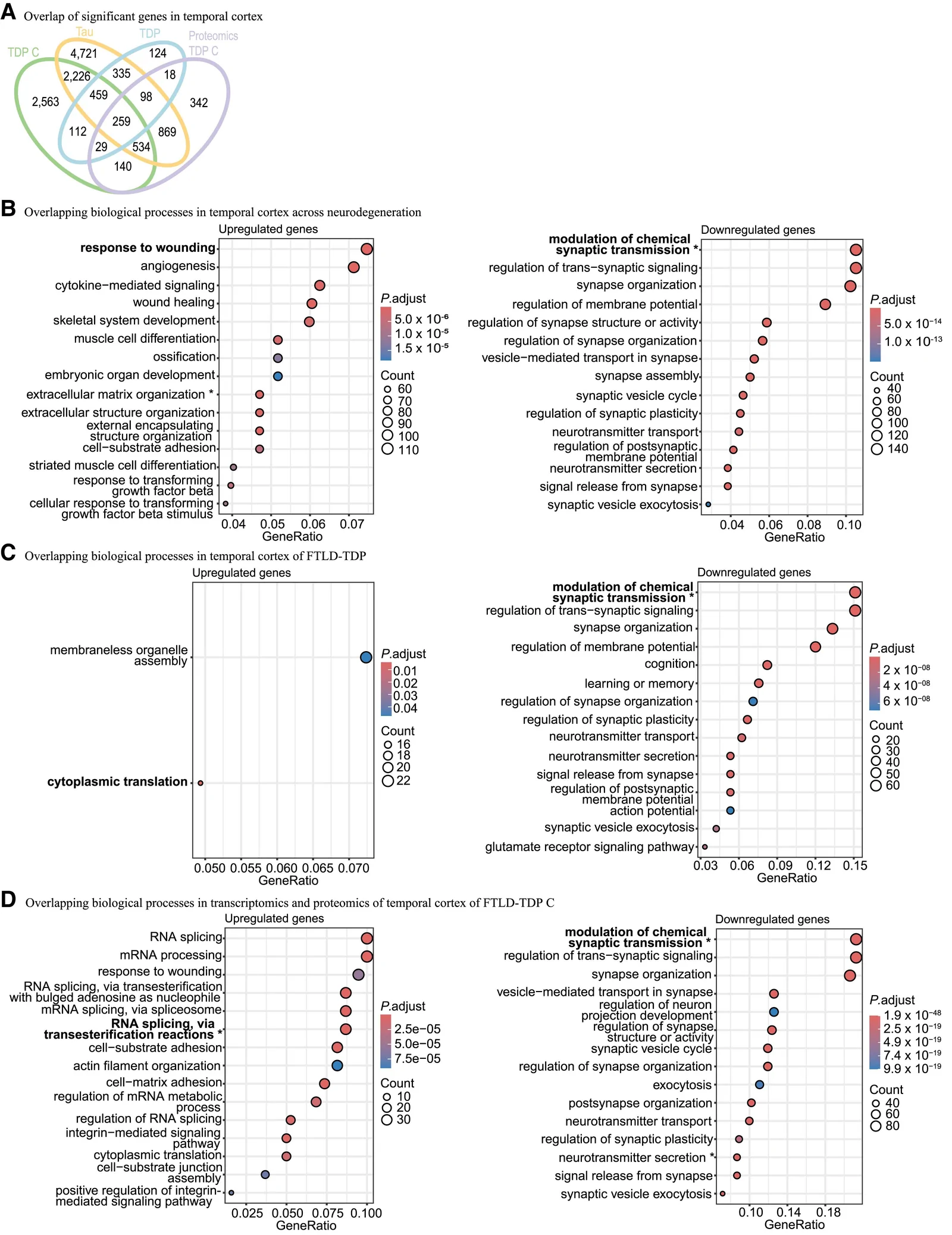
**Overlap between differentially expressed genes in the temporal cortex of FTLD-TDP C, tauopathies, other FTLD-TDP and proteomics of FTLD-TDP C**. (**A**) The Venn diagram depicts the overlap in significantly differentially expressed genes in the temporal cortex across FTLD-TDP C, tauopathies, other FTLD-TDP and proteomics of this FTLD-TDP C cohort. (**B**) Dot plots showing the overrepresented GO terms for biological processes in up- and downregulated differentially expressed genes in FTLD-TDP C which overlapped (both in significance and fold-change direction) with either tauopathies or other FTLD-TDP (aggregated as ‘neurodegeneration’). (**C**) Dot plots depicting overrepresented GO terms for biological processes in up- and downregulated differentially expressed genes that overlapped (both in significance and fold-change direction) with other FTLD-TDP. (**D**) Dot plots visualizing overrepresented GO terms for biological processes in up- and downregulated differentially expressed genes that overlapped (both in significance and fold-change direction) with proteomics of the same FTLD-TDP C cohort and region. All dot plots show the GO terms by significance level and gene ratio. The most significant term of each dot plot is shown in bold. Asterisk denotes a non-redundant term, in the case that multiple terms describe identical or nearly identical gene sets, which can be seen as circles of the same size and significance level. FTLD-TDP = frontotemporal lobar degeneration with underlying TDP-43 proteinopathy; GO term = gene ontology term; *P*.adjust = Benjamini–Hochberg corrected *P*-value; Tau = tauopathies which here indicate Alzheimer’s disease, progressive supranuclear palsy and frontotemporal lobar degeneration with *MAPT* variant; TDP C = frontotemporal lobar degeneration with underlying TDP-43 proteinopathy subtype C.

Focusing solely on genes overlapping between FTLD-TDP C and other FTLD-TDP subtypes, we found 859 (14%) overlapping DEGs (Fig. 2A), of which 854 (14%) were dysregulated in the same direction. The upregulated overlapping DEGs were enriched for the biological processes ‘cytoplasmic translation’ and ‘membraneless organelle assembly’ and for cell components ‘focal adhesion’, ‘ribosome’ and ‘spliceosomal complex’, among others. The downregulated FTLD-TDP common genes were enriched for biological processes involved in synaptic function, plasticity and structure (Fig. 2C and Supplementary Table 2).

In addition, we compared the significantly dysregulated genes detected in the temporal cortex of FTLD-TDP C to previously described dysregulated proteins in the temporal cortex of the same cohort.^18^ We identified 962 (15%) common genes (Fig. 2A), of which 854 (89%) had the same fold-change direction. ‘RNA splicing’, ‘mRNA processing’ and ‘response to wounding’ were overrepresented upregulated processes, as well as cell adhesion-related and cytokine signalling-related processes. Overrepresented downregulated processes were ‘modulation of chemical synaptic transmission’, ‘synapse organization’ and other synaptic functions and structures (Fig. 2D and Supplementary Table 2).

### Synapse, RNA splicing, damage response and transmembrane transport affected in FTLD-TDP C

Module-level analysis of all detected genes using WGCNA identified 17 modules and an 18th rest module containing genes that did not fit into any module (Fig. 3, Supplementary Table 2 and Supplementary Fig. 5). The five modules only correlating to disease status (M1, M11, M13, M16, M17) were deemed most relevant to understanding disease mechanisms. Modules were labelled according to their top overrepresented GO terms (Table 2). M1 was annotated with synaptic function and positively correlated to neurons and astrocytes. Most of the genes in this module were downregulated in FTLD-TDP C. M11 genes, including *TARDBP*, were enriched for RNA splicing and overexpressed in FTLD-TDP C compared to NDC, correlating slightly to several non-neuronal cell types. M13, consisting mostly of genes upregulated in FTLD-TDP C, was annotated as a damage response module and positively correlated to all non-neuronal cell types, except for oligodendrocytes, indicating an upregulated non-neuronal damage response mechanism. M16 represented transmembrane transport and cell binding, with most genes being upregulated. It correlated to endothelial cells, reactive astrocytes and microglia, suggesting that transmembrane transport might be involved in the blood–brain barrier. For M17, no enriched functions were found.

**Figure 3:**
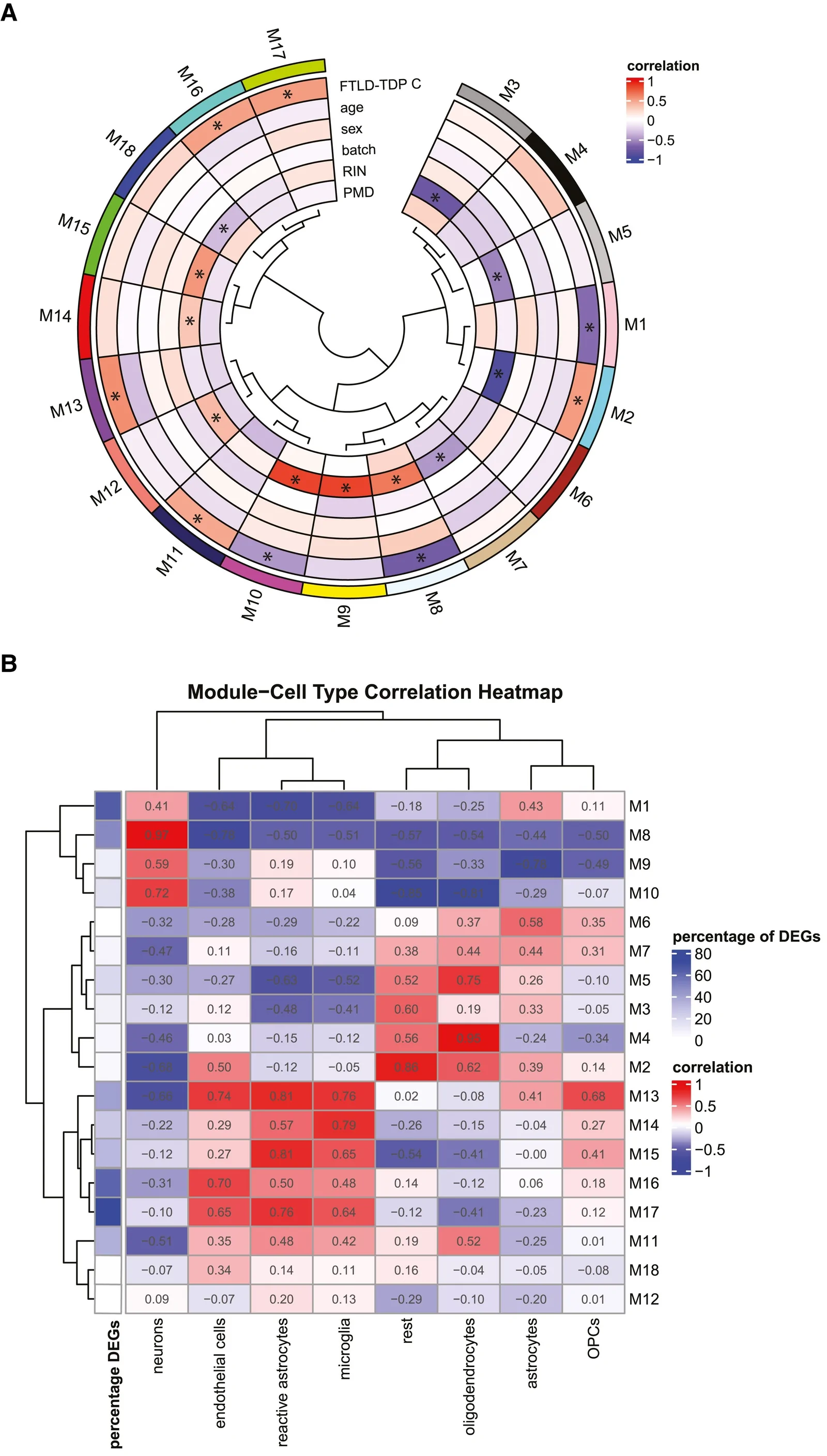
**Modules of co-expressed genes determined using WGCNA.** A total of 18 modules were identified using WGCNA. (**A**) Correlations of modules to FTLD-TDP C, age at death, sex, batch, RIN and PMD are displayed in the circular heat map. An asterisk denotes significant correlations. A dendrogram of the module clustering is depicted in the inner circle. (**B**) Correlations of modules to cell types shown in the heat map demonstrate that modules could be clustered into three groups based on their correlation to cell types: modules correlating positively mainly to neurons (M1 and M8–10), modules correlating to oligodendrocytes and astrocytes (M2–7) and modules correlating to endothelial cells, reactive astrocytes and microglia (M11–18). In addition, the percentages of differentially expressed genes per module are visualized in the first column. Age = age at death; DEGs = differentially expressed genes, false discovery rate <0.05; FTLD-TDP C = frontotemporal lobar degeneration with underlying TDP-43 proteinopathy subtype C; PMD = post-mortem delay; RIN = RNA integrity number; WGCNA = weighted gene co-expression network analysis.

**Table 2:** Labels of weighted gene co-expression network analysis modules

Module	Module size	Label
M1	1280	Synapse I
M2	3990	Reproductive pathways
M3	183	No overrepresented pathways
M4	854	Myelination
M5	157	Cell matrix adhesion
M6	47	Ribonucleoprotein
M7	653	Protein folding
M8	7059	Synapse II
M9	4598	Cytoplasmic RNA processing
M10	207	Mitochondrial respiration
M11	454	RNA splicing
M12	166	DNA biosynthetic processes
M13	1301	Damage response
M14	228	Immune reaction
M15	1250	Catabolic processes
M16	55	Transmembrane transport and cell binding
M17	61	No overrepresented pathways

The labels given to the modules based on overrepresentation analysis are listed in the table, as well as the number of co-expressed genes per module.

### Dysregulation of synaptic processes in FTLD-TDP C

We performed cell deconvolution analysis and corrected for potential cell type composition differences in neurons and glia driving differential gene expression between FTLD-TDP C and NDC. After adjusting for differences in cell type composition, 609 FTLD-TDP C genes were significantly differentially expressed, of which 223 were up- and 386 were downregulated (Fig. 4A, Supplementary Table 1 and Supplementary material). Among the downregulated DEGs, after adjustment for cell type composition, synapse-related cell components were overrepresented, whilst among the upregulated DEGs, no overrepresented pathways were found (Fig. 4B). The top three overrepresented non-redundant cell compartments were ‘neuron to neuron synapse’, ‘postsynapse’ and ‘dendrite’.

**Figure 4:**
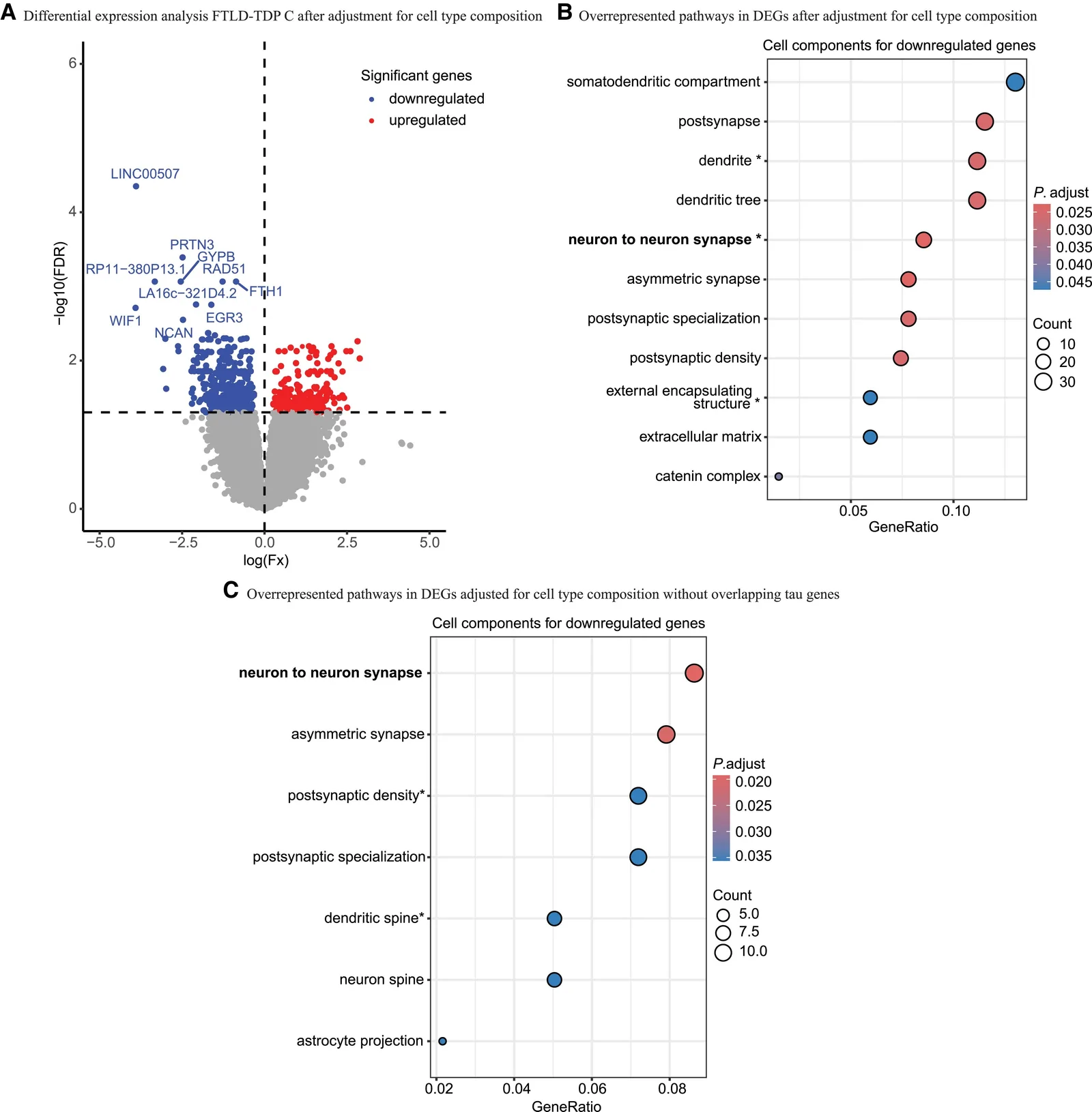
**Differentially expressed genes in FTLD-TDP C after adjustment for cell type composition**. (**A**) The volcano plot shows differentially expressed genes after adjustment for cell-type composition, depicting significantly upregulated genes in red and significantly downregulated genes in blue. Fold changes are shown on the *x*-axis, while *P*-values corrected for multiple testing using Benjamin–Hochberg false discovery rate are shown on the *y*-axis. The top 10 most significant genes are labelled. (**B**) The *top* dot plot depicts the overrepresented GO terms for cell components in downregulated differentially expressed genes adjusted for cell type composition, while (**C**) the *bottom* dot plot depicts the overrepresented GO terms for downregulated FTLD-TDP-related differentially expressed genes, from which genes shared with tauopathies have been removed. The dot plots show the GO terms by significance level and gene ratio. The most significant term of each dot plot is shown in bold. Asterisk denotes a non-redundant term, in the case that multiple terms describe identical or nearly identical gene sets, which can be seen as circles of the same size and significance level. DEGs = differentially expressed genes, FDR < 0.05; Fx = fold change; FDR = false discovery rate corrected *P*-value; FTLD-TDP C = frontotemporal lobar degeneration with underlying TDP-43 proteinopathy subtype C; GO term = gene ontology term; *P*.adjust = Benjamini–Hochberg corrected *P*-value.

To identify FTLD-TDP-related DEGs, we removed genes that were also dysregulated in the temporal cortices of tauopathies, namely AD, PSP and FTLD-MAPT (Supplementary material and Supplementary Table 3), leaving 247 genes (103 upregulated, 144 downregulated; Supplementary Table 1). When comparing these FTLD-TDP genes to proteomics hits from the temporal cortex of the same patients in this post-mortem cohort,^18^ we identified 43 of these genes (17%) to be significantly dysregulated at the protein level too, with 31 (13%) exhibiting the same fold-change direction (Supplementary Table 1). In the 247 FTLD-TDP genes, similar synapse-related cell components were overrepresented, as was found prior to filtering, with ‘neuron to neuron synapse’ again being the most significantly enriched term (Fig. 4C and Supplementary Table 4). After removing genes that were also dysregulated in the temporal cortex of other FTLD-TDP subtypes, we identified 225 FTLD-TDP C-specific genes (100 upregulated, 125 downregulated). This subset showed no statistically significant functional enrichment. Consequently, we used the FTLD-TDP set of 247 genes, including genes overlapping with other TDP subtypes, for further analysis of synaptic processes using SynGO (Supplementary Table 5). Within the synaptic genes of this FTLD-TDP gene set, we found enrichment of postsynaptic structures (Fig. 5A), in particular, the enrichment of genes involved in the ‘modulation of chemical synaptic transmission’ and ‘neurotransmitter receptor localization to the postsynaptic specialization membrane’ (Fig. 5B). The gene network behind these synaptic processes is visualized in Fig. 6 and contains 28 genes, of which 22 (79%) were FTLD-TDP C-specific (in bold and italic in Fig. 6 and Supplementary Table 5). In addition, of the 28 genes within the network, 15 (54%) were also significantly dysregulated at the protein level (delineated in the network in Fig. 6), of which 13 (46%) showed the same fold-change direction (solid delineation in the network in Fig. 6; see also  Supplementary Tables 1 and 5), affirming the relevance of these processes in FTLD-TDP C. Eleven genes in this synaptic gene network are both specific to FTLD-TDP C and dysregulated at the protein level: *BEGAIN*, *CADM1*, *COMT*, *ITPKA*, *LRFN1*, *NCAN*, *RAB3B*, *RGS14*, *SLC1A2*, *SYNPO*, *TUBB2B*—potentially key drivers of synaptic pathology in FTLD-TDP C, detectable at both the RNA and protein levels.

**Figure 5:**
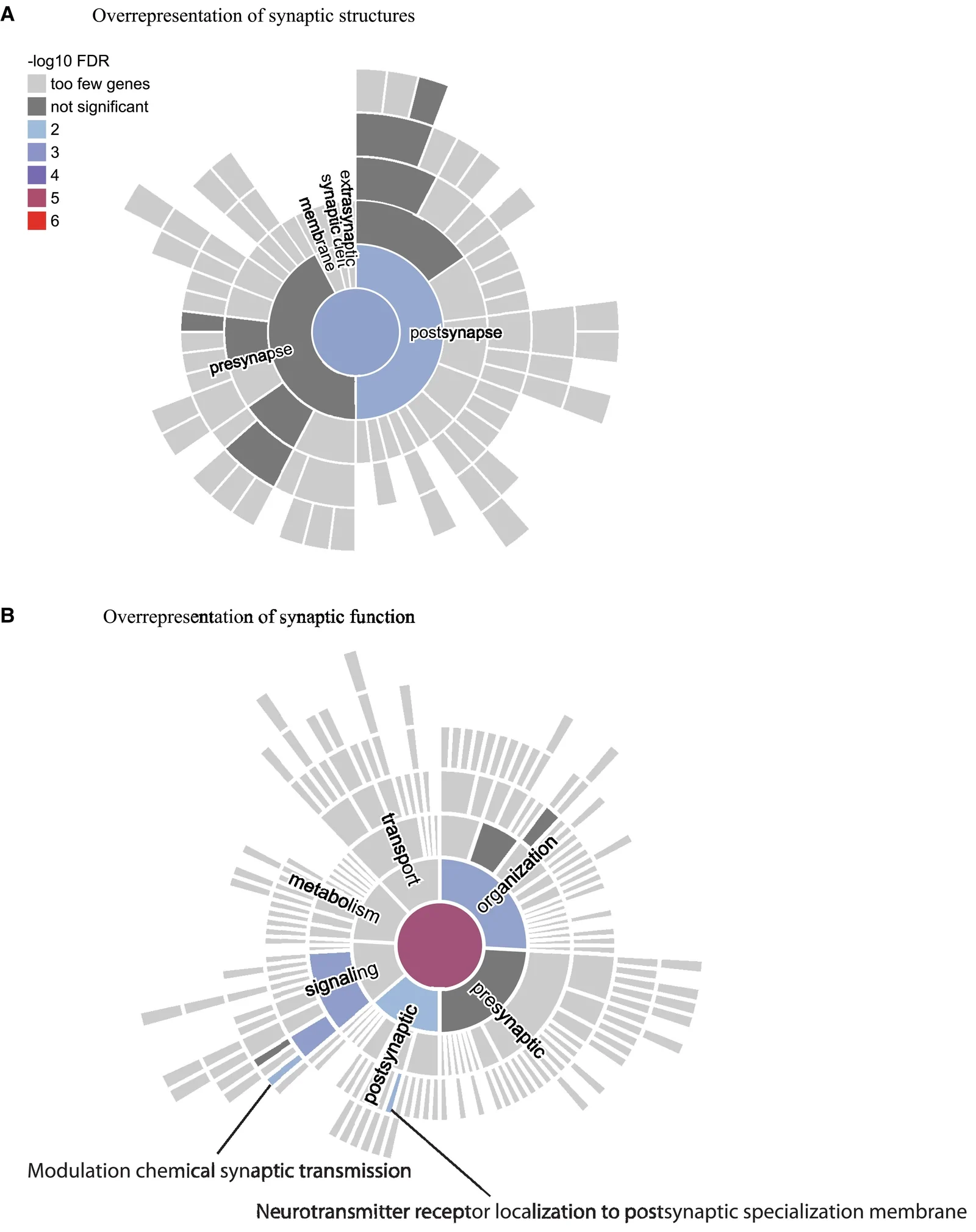
**Enrichment of synaptic gene ontology terms in FTLD-TDP genes.** (**A**) The circular plots show SynGO enriched terms of FTLD-TDP genes, i.e. differentially expressed genes after correction for cell composition differences in FTLD-TDP C from which overlapping tau-related genes were removed, for synaptic structures and (**B**) synaptic function with colour-coded significance levels. The centre of the circles represents generic terms, whereas outer layers represent more specific terms. FDR = false discovery rate corrected *P*-value; FTLD-TDP C = frontotemporal lobar degeneration with underlying TDP-43 proteinopathy subtype C.

**Figure 6:**
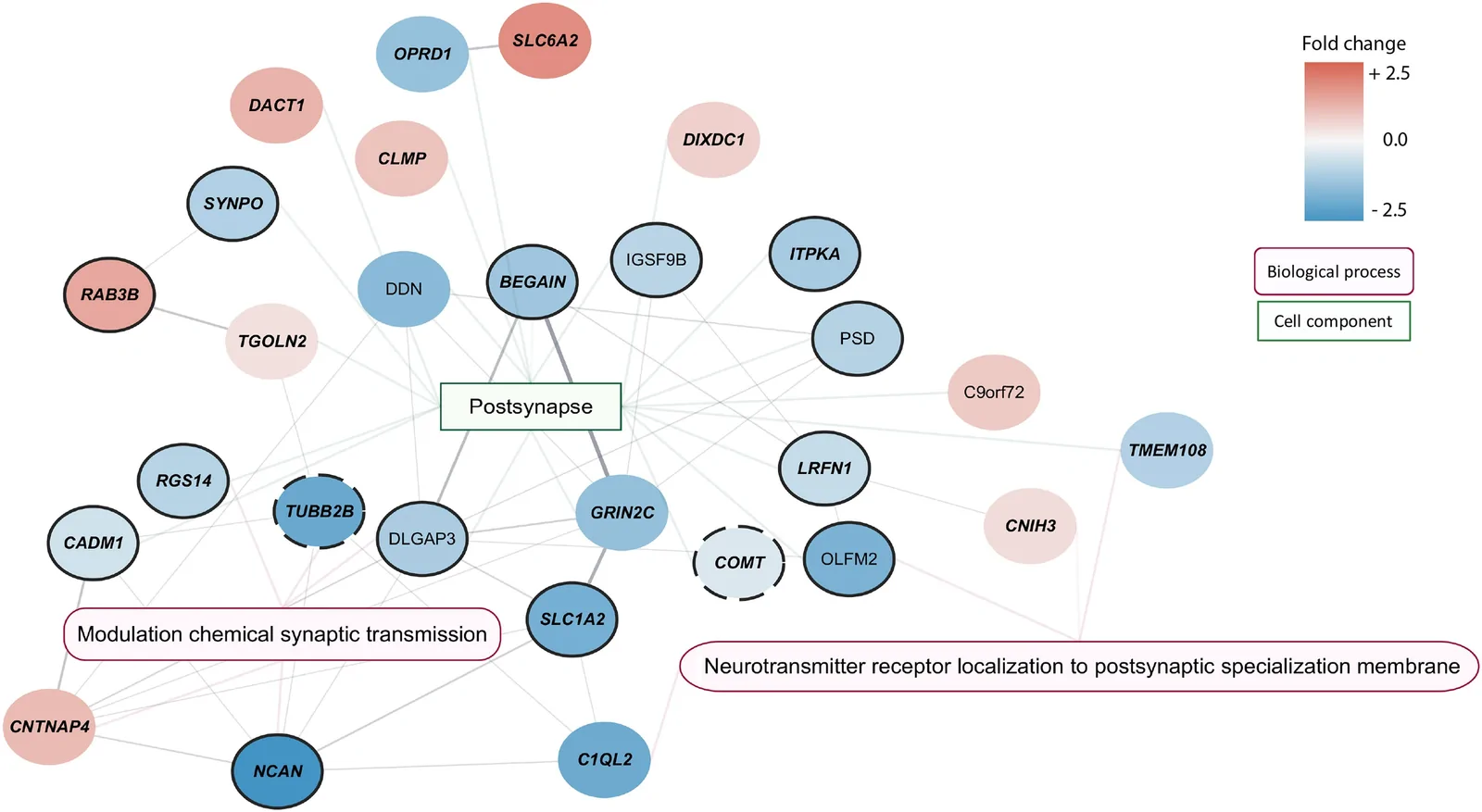
**Gene network of synaptic genes dysregulated in FTLD-TDP C.** Genes are colour-coded according to fold-change direction. In addition, genes are connected to the enriched SynGO terms for synaptic structure and function. Genes in bold and italic were specific to FTLD-TDP C. Solid delineation indicates genes that were significant with the same fold-change direction in the proteomics data of the temporal cortex, whereas dashed delineation indicates genes that were significant with a different fold-change direction in the proteomics data. FTLD-TDP C = frontotemporal lobar degeneration with underlying TDP-43 proteinopathy subtype C.

## Discussion

This is, to our knowledge, the first study to assess gene expression in the human temporal cortex of FTLD-TDP C patients using bulk RNA sequencing. We described gene expression differences and investigated specific alterations in this unique post-mortem cohort of FTLD-TDP C cases (*n* = 18), with age- and sex-matched NDC (*n* = 23), for which mass spectrometry^17,18^ has been performed previously. In FTLD-TDP C, processes related to immune response, cell structure, RNA splicing and transmembrane transport were upregulated, and synaptic processes were downregulated. Particularly at the postsynapse, we found dysregulation of signal transmission and assembly of the postsynaptic membrane after adjusting for cell-type composition and filtering genes previously found to be dysregulated in transcriptome studies of tauopathies. Moreover, more than half of the genes involved in these dysregulated postsynaptic processes were also disrupted at the protein level, emphasizing the relevance of these processes in the disease mechanisms in FTLD-TDP C and directing future research.

RNA bulk sequencing of the temporal cortex yielded 6322 significant DEGs, representing approximately 27% of all detected autosomal protein-coding genes, indicating that many biological processes are affected in the left temporal cortex of end-stage FTLD-TDP C. Indeed, this is the region from which the disease initiates, and it is clinically^3,4^ and pathologically^5,37^ the most affected. The hallmark inclusions of TDP-43 were reflected at the RNA level by moderate overexpression of its gene, *TARDBP*. Similarly, proteomics showed higher protein abundance of TDP-43 in the temporal cortex.^18^ Moreover, *TARDBP*’s RNA targets as well as protein interactors were enriched in the significant genes, affirming the relevance of *TARDBP* dysregulation in this TDP-43 proteinopathy. Interestingly, ANXA11, which has recently been found to co-aggregate with TDP-43 in FTLD-TDP C,^8,9^ was not significantly dysregulated at the RNA level in the temporal cortex, yet was overexpressed at the protein level.^18^ This suggests that dysfunction of other processes, such as post-transcriptional modification or protein degradation, may contribute to ANXA11 aggregation, or, alternatively, that its transcription may be upregulated at an earlier stage of the disease, leading to aggregates which are not efficiently cleared.

Module-level analysis further revealed dysregulation of RNA splicing processes (M11) in FTLD-TDP C. We also identified RNA splicing as an enriched GO term among significant genes that were likewise dysregulated at the protein level. TDP-43 is involved in RNA splicing^38^ and loss of this protein has been shown to induce erroneous splicing with introduction of cryptic exons.^28,39^ Although this process seems relevant to the disease mechanism, it is likely not specific to FTLD-TDP C, as we found the spliceosomal complex to be enriched in the DEGs shared between our FTLD-TDP C cohort and that of another transcriptomics study on temporal cortex of FTLD-TDP (mix of subtypes A and B).^40^ RNA splicing did not remain enriched as a GO term after cell deconvolution and filtering of commonly affected genes, further underscoring the commonality of this process in FTLD-TDP.

In addition, several commonly affected neurodegenerative processes were disturbed in FTLD-TDP C. Changes in processes involved in cell structure and extracellular matrix, as well as immune response, were upregulated among the significant genes. Similarly, through co-expression analysis, we identified one of the FTLD-TDP C modules to encompass a non-neuronal damage response (M13). When comparing our results to other FTLD and AD, we found half of our significant genes to be dysregulated similarly and the overlapping upregulated genes to be enriched for functions related to damage and immune response. These processes likely reflect severe neurodegenerative changes in the highly atrophic temporal cortices of FTLD-TDP C and were therefore not enriched in the gene subset after removing genes commonly affected by neurodegeneration. Using WGCNA, we also identified dysregulation of transmembrane transport and cell binding, particularly in endothelial cells, reactive astrocytes and microglia. As these cell types are part of the neurovascular unit, dysregulation of transport across them could implicate blood–brain barrier disruptions. Previously, single-nucleus RNA sequencing has shown indications of blood–brain barrier disruption in cortical regions of FTLD-TDP A with underlying progranulin mutation.^10^ Among the DEGs shared in FTLD-TDP, at the gene and protein level, we found enrichment of cell adhesion components, which could play a role in maintaining tissue structure and consequently blood–brain barrier integrity. Although no directly transport-related biological processes were found to be common in FTLD-TDP, possibly neurovascular dysfunction is a shared process in the different FTLD-TDP subtypes.

Of the various dysregulated gene functions, only postsynaptic processes remained significantly enriched among downregulated genes after filtering out tau-related genes. Notably, this enrichment persisted even after correcting for neuronal loss in the temporal cortex, indicating that these processes were downregulated within the remaining active neurons, rather than simply reflecting a reduction in neuronal population. Two modules of co-expressed genes correlating to FTLD-TDP C also represented neuronal synaptic processes, underscoring the robustness of this finding. As synaptic functions were found to be enriched across all comparisons (genes common in tau and FTLD-TDP, genes common in FTLD-TDP and genes dysregulated at the protein level too), these are presumably significantly affected in dementia with underlying tauopathy or TDP-43 proteinopathy. We focused on a gene network of synaptic genes found in our FTLD-TDP C data, without excluding genes overlapping with other FTLD-TDP subtypes, as the filtered FTLD-TDP C-specific gene set showed no significantly enriched functions. We retrieved ‘modulation of chemical synaptic transmission’ and ‘neurotransmitter receptor localization to postsynaptic specialization membrane’ as enriched gene ontology processes in these synaptic FTLD-TDP-related genes. Most of the synaptic genes were involved in excitatory synapses. In the context of FTLD-TDP, both downregulation of excitatory^41,42^ and inhibitory synapses^43^ have been described in pathological studies on human brain tissue. In the case of FTLD-TDP C here, the synaptic imbalance was characterized by diminished postsynaptic excitation. In a recent whole-genome sequencing study of FTLD-TDP patients,^44^ enrichment of excitatory postsynaptic potential was identified in the FTLD-TDP C subgroup through pathway analysis of prioritized common variants, corroborating our findings.

Although the 225 differentially expressed FTLD-TDP C-specific genes showed no significantly functionally enriched GO terms, genes with known synaptic roles were identified among them. In fact, within the gene network of 28 synaptic FTLD-TDP genes, 22 were distinct for FTLD-TDP C, indicating that although certain synaptic processes might be shared within FTLD-TDP, within this network, most are characteristic of the subtype FTLD-TDP C.

To further confirm our findings, we compared our gene expression results to the proteomics results from temporal cortex of the same post-mortem FTLD-TDP C cohort.^18^ When comparing all significant genes to proteins, 15% of the genes were dysregulated at the RNA and protein levels. Similarly, in comparing DEGs after correcting for cell type composition differences, a 15% overlap at the protein level was found. Upon filtering for genes in common with tauopathies, we observed a slight increase to 17% overlap. Correlations between RNA and protein expression are notoriously low to moderate,^45,46^ making these findings unsurprising. Potentially subtle changes in gene and protein expression may not be detectable at both the transcriptomic and proteomic levels. However, it is interesting that in our synaptic gene network of interest, curated after filtering steps and enrichment analyses, we found a 54% overlap between significant genes and proteins, with 46% exhibiting the same direction of change, suggesting that these synaptic processes are severely affected and detectable at both the RNA and protein levels.

We focused on the 11 synaptic FTLD-TDP C-specific genes within our gene network, affected at both the RNA and protein levels in the temporal cortex, as these most likely reflect biologically relevant pathophysiological processes in FTLD-TDP C: *BEGAIN*, *CADM1*, *COMT*, *ITPKA*, *LRFN1*, *NCAN*, *RAB3B*, *RGS14*, *SLC1A2*, *SYNPO*, *TUBB2B*. Among these genes, two code for synaptic adhesion molecules: *CADM1* and *NCAN*. Our previous study on the dentate gyrus proteome of this FTLD-TDP C cohort showed an increase in cell adhesion proteins at the synaptic membrane, crucial for signalling.^17^ Similarly, RNA sequencing of the frontal cortex in FTLD-TDP subtype A found upregulated cell adhesion genes.^43^ Although the cell adhesion-related genes from our RNA sequencing data differ from those previously described and are downregulated rather than upregulated, synaptic adhesion appears to be commonly dysregulated. The discrepancy in expression pattern might reflect brain region-specific changes. *NCAN* variants have been associated with other neurodegenerative diseases,^47,48^ whilst *CADM1* exosome-derived mRNA expression has shown associations with AD diagnosis^49^ and cognitive performance,^50^ implying a potentially broader role in neurodegeneration, although not disrupted in RNA sequencing data from tauopathies.

Four of these genes affect signal transmission*: COMT*, *RGS144*, *SLC1A2* and *TUBB2B*. Notably, *COMT* and *TUBB2B* exhibited opposite fold-change directions at the RNAand protein levels, with lower gene and higher protein expression, suggesting perhaps decreased protein degradation, a potential negative feedback loop or simply coincidental overlap. *RGS14*^51^ and *COMT*^52^ have been associated with Parkinson’s disease, while *SLC1A2* was downregulated in a progranulin knockout mouse model of FTLD-TDP A,^53^ indicating that these genes involved in signal transmission are also affected in other neurodegenerative diseases.

Finally, five synaptic genes regulate synaptic plasticity: *BEGAIN*, *ITPKA*, *LRFN1*, *RAB3B*, *SYNPO*. Of these, only *ITPKA* has been associated with neurodegenerative diseases,^54^ suggesting that the other synaptic plasticity-related genes are possibly uniquely affected in FTLD-TDP C. Future research should investigate the complex interactions between the synaptic genes dysregulated in FTLD-TDP C in various *in vitro* and *in vivo* models, using electrophysiological and imaging experiments. Better understanding of these pathways could lead to the identification of novel biomarkers or even therapeutic targets for this subtype.

In this bulk RNA sequencing study of FTLD-TDP C, we combined differential expression with co-expression analysis to create a global overview of dysregulated genes and biological processes. We used published RNA sequencing data from the temporal cortex in dementias and related neurodegenerative diseases to uncover common processes and those specific to FTLD-TDP. Furthermore, we conducted in-depth analyses by deconvoluting our data by cell type, filtering out non-specific genes and comparing our findings with proteomics results from the same cohort and region to identify dysregulated processes.

Here, we studied the most affected brain region at the end stage of this disease, so we were unable to draw conclusions about the timing of the gene expression changes described in the disease process. Possibly, relevant disease-specific mechanisms occur earlier in the disease process, whereas mostly similarly dysregulated features across neurodegeneration are left to the final stages. Even though brain tissue from patients in the early stage of the disease is quite rare, a future study using brain tissue from patients in earlier phases of the disease (e.g. deceased due to competing diseases) could uncover prior disease mechanisms. Future research could also include multiple brain regions to assess varying degrees of affectedness and potentially discern a disease-dissemination pattern, using, for instance, spatial transcriptomics. This could also identify region-specific changes.

For our comparisons with FTLD-TDP RNA sequencing data from the temporal cortex, we were limited to one dataset^40^ with a mixed FTLD-TDP and ALS cohort (subtypes A and B). To further characterize the different FTLD-TDP subtypes, more data from the separate subtypes would be beneficial. As FTLD is a relatively uncommon disease, acquiring large datasets can be difficult. In future, efforts to increase cohort sizes in post-mortem studies of distinct FTLD subtypes could benefit discriminative power.

In this post-mortem cohort, we reported mild ageing-related co-pathology, such as early Alzheimer pathology, ARTAG and PART, in temporal cortices of both NDC and FTLD-TDP C, which occurred more often in NDC than in FTLD-TDP C, though did not differ significantly. The described gene expression differences between FTLD-TDP C and NDC may therefore reflect differences in co-pathology too, limiting the specificity of our findings. Potentially, the detected differences underestimate the actual gene expression differences, given that the NDC in this study showed more neurodegenerative co-pathology. Our findings should thus be interpreted as the predominant differences in gene expression between FTLD-TDP C and NDC, but possibly not the only ones. As co-pathology is unavoidable in the brain tissue of individuals of a certain age, future studies should attempt to have a balanced distribution of co-pathology between disease and control tissue.

Furthermore, it is important to note that in our differential expression analysis we corrected for RIN, as a multivariate analysis revealed grouping of samples based on RIN, with disease samples having lower RIN values. Given that we were not interested in gene expression differences based on tissue quality, we sought to remove this noise by correcting for RIN. However, since RIN was also correlated with disease status, this correction might have led to the overadjustment of gene expression differences between groups. Future studies should aim to have similar RIN across all samples to avoid this potential bias.

Finally, although we attempted to correct for differences in cell-type composition across samples, single-cell/nucleus RNA sequencing could provide higher resolution and elucidate expression differences at the cell level.

## Conclusion

To conclude, glial damage response, RNA splicing and transport across the neurovascular unit are upregulated, while synaptic processes are downregulated in the temporal cortex of FTLD-TDP C patients. Although FTLD-TDP C is a clinically and pathologically distinct subtype, many genes and functions overlap with tauopathies and other FTLD-TDP subtypes, indicating that the disease mechanisms are likely similar, at least at the end stage of disease in post-mortem brains. Postsynaptic excitation was downregulated after correcting for differences in cell-type composition and filtering out genes in common with tauopathies. In particular, the assembly of receptors at the postsynaptic membrane and synaptic signal transmission were affected at both the RNA and protein levels. Future studies should focus on these postsynaptic processes, especially on the genes involved in synaptic plasticity that have not yet been described in other neurodegenerative diseases, to shed light on the distinct pathophysiological mechanisms and pave the way for targeted clinical approaches.

## Supplementary Material

awag085_Supplementary_Data

## Data Availability

The authors confirm that the data supporting the findings of this study are available within the article and its Supplementary material.
