# Supplementary material for: Transcriptomic signature of frontotemporal lobar degeneration with TDP-43 type C pathology
Source: Brain. 2026 Mar 6;149(11):3875–89. doi: 10.1093/brain/awag085 (PMC13730574; doi:10.1093/brain/awag085)
Supplement: Supplementary file 1 [file awag085_supplementary_data.zip › brain-2025-02148-File010.pdf]

Supplementary materials

Other RNA sequencing datasets

To retrieve data from studies of RNA sequencing from brain tissue of dementia, the following PubMed search was conducted on 22.04.2024:

((RNA-sequencing OR transcriptomic*[tiab]) AND dementia*[tiab] AND (cortex OR brain-tissue)) NOT (animals[mh] NOT humans[mh])

This resulted in retrieval of 203 unique articles. Articles were included if they described bulk RNA sequencing from the temporal cortex of a subtype of dementia and had more than ten samples per group. Articles were excluded if the studied disease was ill-defined, other RNA sequencing techniques were used (e.g. snRNA, scRNA, miRNA), other brain regions were sampled, group sizes were smaller than ten, other human tissues were used, and if described data was only used for replication or methodological purposes. Reviews and case reports were also excluded. After scanning titles, 129 articles were excluded (95 not relevant, 17 not RNA sequencing, eight other tissue, eight reviews, one case report), and after reading abstract and methods sections another 68 were excluded (23 other RNA sequencing, 17 other brain regions, nine n<10, eight not original data, seven not on human tissue, two methodological papers, two ill-defined case group). The selection resulted in six articles, describing a total of four datasets. One was a preprint at the time, but was published shortly afterwards.[1]

In addition, the NCBI GEO database was searched on 30.05.2024 using the following search:
((RNA-sequencing OR transcriptomic*) AND dementia* AND (cortex OR brain-tissue))
The same in- and exclusion criteria were applied. No additional datasets were found through this search.

Ultimately, four datasets were retrieved: the University of Edinburgh's dataset of FTLD with MAPT 10+16 deletion (FTLD-MAPT) from the superior temporal gyrus (https://doi.org/10.7488/ds/7790),[1] the New York Genome Center's (NYGC) dataset of FTLD-

TDP from the temporal cortex (acquired by contacting first author),[2] the Mount Sinai Brain Bank's (MSBB) dataset of Alzheimer's disease (AD) and mild cognitive impairment (MCI) from the superior temporal gyrus (AD Knowledge Portal Synapse syn22447899) and the Mayo Clinic Brain Bank's (Mayo) dataset of AD and progressive supranuclear palsy (PSP) from the temporal cortex (AD Knowledge Portal Synapse syn5550404). Each dataset had its own non-demented control group. Baseline characteristics and raw gene count tables of the datasets were acquired. For differential expression analysis linear regression was performed correcting for age, sex, batch, RIN and PMD.

Statistical analysis of cohort characteristics

Descriptive statistics were used to assess cohort characteristics. For our FTLD-TDP C dataset (FTLD-TDP C versus controls), the NYGC dataset (FTLD-TDP versus controls) and the Edinburgh dataset (*MAPT* versus controls), differences between parametric baseline variables were determined using the independent samples t-test. Non-parametric variables were compared using the Mann-Whitney U test, whereas categorical variables were compared using the chi-square test or the Fisher's exact test (when $n<5$ for subgroup). For MSBB (AD, MCI, controls) and Mayo data (AD, PSP, controls), ANOVA was used for parametric variables, the Kruskall-Wallis test for non-parametric variables with the Dunn test for post-hoc comparisons. For categorical variables either the chi-square test or the Fisher's exact test (when $n<5$ for subgroup) was used with post-hoc pairwise comparisons. The two-sided significance level was set at $\alpha=0.05$. Multiple testing was corrected for using the Bonferroni method.

Assessing enrichment for *TARDBP*-related genes

Genes were considered to be RNA targets of *TARDBP* if their expression had been altered in at least two original papers on human cell lines with TDP-43 knockdown/out (n=76). To be considered protein interactors, genes had to be found both in the String network of *TARDBP* and in the experimentally determined binding partners of TDP-43 protein in the meta-analysis by Helmold et al.[3] ($n=72$). There was no overlap in the curated RNA targets and protein interactors.

Cell deconvolution

The parameters of the CIBERSORT function were set as follows: p=1000, absolute=F and QN=F (1000 permutations, relative cell proportions used, no quantile normalization), based on CIBERSORTx' recommendations for RNA sequencing data. Initially, 75 transcriptionally distinct cell types were annotated from the signature data (snRNA sequencing of medial temporal gyrus from Allen Human Brain Atlas),[4] consisting of 69 neuronal and 6 non-neuronal subtypes (endothelial cells, microglia, oligodendrocytes, OPCs, astrocytes (SLC14A1 positive) and reactive astrocytes (GFAP positive)). Correction for cell types was deemed relevant, if a difference >5% between cases and controls was found, according to recommendations by Sutton et al..[5] Since the difference in cell composition between FTLD-TDP C and controls was <5% for all of these 75 cell types (23 cell types were not found in either group and only 22 cell types were above 1% present in both groups), the number of cell types used for cell deconvolution was reduced by congregating neuronal markers as one signature for neurons. This resulted in seven remaining cell types: neurons, endothelial cells, microglia, oligodendrocytes, OPCs, astrocytes (SLC14A1 positive) and reactive astrocytes (GFAP positive). In FTLD-TDP C versus controls neurons were -39% present, oligodendrocytes +14%, reactive astrocytes +10% and endothelial cells +5%, whilst for other cell types the difference was <5%. Supplementary Fig. 2 shows how cell types contributed to clustering of samples. Cell composition differences of neurons, oligodendrocytes and reactive astrocytes mostly affected clustering of samples. Since neurons and oligodendrocytes had a mirrored pattern, we only corrected for neurons. In addition, we corrected for composition differences in reactive astrocytes.

Co-expression analysis

We performed single-block clustering of all genes simultaneously, with the soft-thresholding power set to 5, the minimum module size set to 30 and the merge height for clustering set to 0.25. For functional annotation of the modules ORA was run and redundant enriched GO terms were manually curated based on overlap in involved genes per term. Using enrichplot() GO plots were created for each of the three subcategories BP, CC and molecular function (MF). To summarise the most relevant GO terms, per module the top three most significant, non-redundant

terms per subcategory were selected by reviewing output tables from ORA and eyeing end-branch terms on the GO plots. Based on the top three GO-terms per category, a label was given to each module. If two modules had similar GO terms, an additional ORA was run in g:Profiler web server (v. e111_eg58_p18_f463989d) comparing the genes of the two modules, by setting genes of one module as input and genes of both modules as background, and using the Benjamini-Hochberg correction for multiple testing ($P<0.05$). This aided in setting distinctive labels for modules.

Supplementary Results

Multivariate analysis

Principal component analysis (PCA) showed a clear distinction between FTLD-TDP C and NDC across PC1 and PC2 (Supplementary Fig. 3A). Two cases were found grouped amongst NDC; one of which (#29) had undergone euthanasia. In addition, for this case, the right temporal cortex had been sampled, whilst there was asymmetrical atrophy to the left, which means the less affected side was analysed. Possibly, the sequenced tissue of this case had not reached end-stage disease as the tissues of other cases, which explains its grouping among NDC. For the other case (#1) no rationale for grouping among NDC was identified, other than biological variability within the diseased phenotype. Hierarchical clustering analysis (HCA) clustered cases and controls separately too (Supplementary Fig. 3B). Three cases were found among the controls, all of which were also grouped with or near NDC in PCA. Interestingly, the two cases with somatic variants in *TARDBP* (#18 in dentate gyrus, #30 in medial temporal gyrus) clustered together among the cases. Positioning of samples across PC1 and PC2 seemed to be determined not only by disease status, but also by RIN (Supplementary Fig. 3C). RIN is a proxy for tissue quality and consequently difference between cases and controls based on this technical variable were not deemed interesting. Hence, correction for RIN was applied in the differential expression analysis to detect true-positive DEG. Distribution of samples across PC1 and PC2 did not appear to be driven by PMD (Supplementary Fig. 3D), nor by the sampled subregion within the temporal cortex (Supplementary Fig. 3E). No samples were excluded based on multivariate analyses, as no striking outliers were identified. Cases grouping amongst controls were considered part of biological variability that should be retained in this limited sample size.

Other RNA sequencing datasets

PCA and HCA of the other RNA datasets used for comparisons showed that clustering of samples was affected by age at death, RIN, PMD and batch. To account for any confounding based on differences in these variables, the linear regression model used for differential expression analysis was corrected for age at death, RIN, PMD, batch and sex. In one of the datasets, the Mayo dataset, two outliers (one control, one PSP) were identified and excluded from analysis (Supplementary Fig. 4). Differential expression analysis found 8792 DEG in AD, 569 in PSP and 5809 in FTLD-MAPT. These genes were used for comparisons and filtering of commonly affected DEG in FTLD-TDP C (Supplementary Table 2). For comparison of FTLD-TDP-related-genes, 1434 significant genes found in the temporal cortex of the NYGC FTLD-ALS cohort [2] were used (Supplementary Table 2).

Comparing cell deconvoluted differentially expressed genes to other datasets

We identified 609 DEG in the temporal cortex of FTLD-TDP C after adjusting for differences in cell-type composition, and compared them to DEG identified in the other transcriptomics data from the temporal cortex. In the comparison with tauopathies and other FTLD-TDP subtypes, we found 407 (67%) DEG to overlap, of which 220 (36%) had the same fold-change direction. Among the latter, 'trans-synaptic signaling' and 'synapse organization' were enriched biological processes in the downregulated genes (Supplementary Table 4). Among the common upregulated genes there was no functional enrichment of GO terms. When comparing the 609 DEG after correction for cell-type composition in FTLD-TDP C to other FTLD-TDP subtypes, 87 (14%) common FTLD-TDP genes were identified, with 85 (14%) having the same direction of change. Functional enrichment analysis found three cell components among the downregulated common FTLD-TDP genes, namely 'postsynaptic specialization', 'postsynaptic density' and 'transport vesicle membrane' (Supplementary Table 4). Here too, no enriched terms were found among the upregulated common genes. Finally, comparing these 609 DEG after adjustment for cell type composition to the proteomics findings on the temporal cortex of the same cohort of FTLD-TDP C,[6] we found 90 (15%) overlapping genes/proteins, of which 75 (12%) were dysregulated in the

same direction. Overrepresentation analysis revealed synapse-related GO terms enriched among the downregulated genes, i.e. ‘modulation of chemical synaptic transmission’, ‘synapse organization’ and ‘regulation of synaptic plasticity’ (Supplementary Table 4). No terms were enriched among the shared upregulated genes/proteins.

Figures

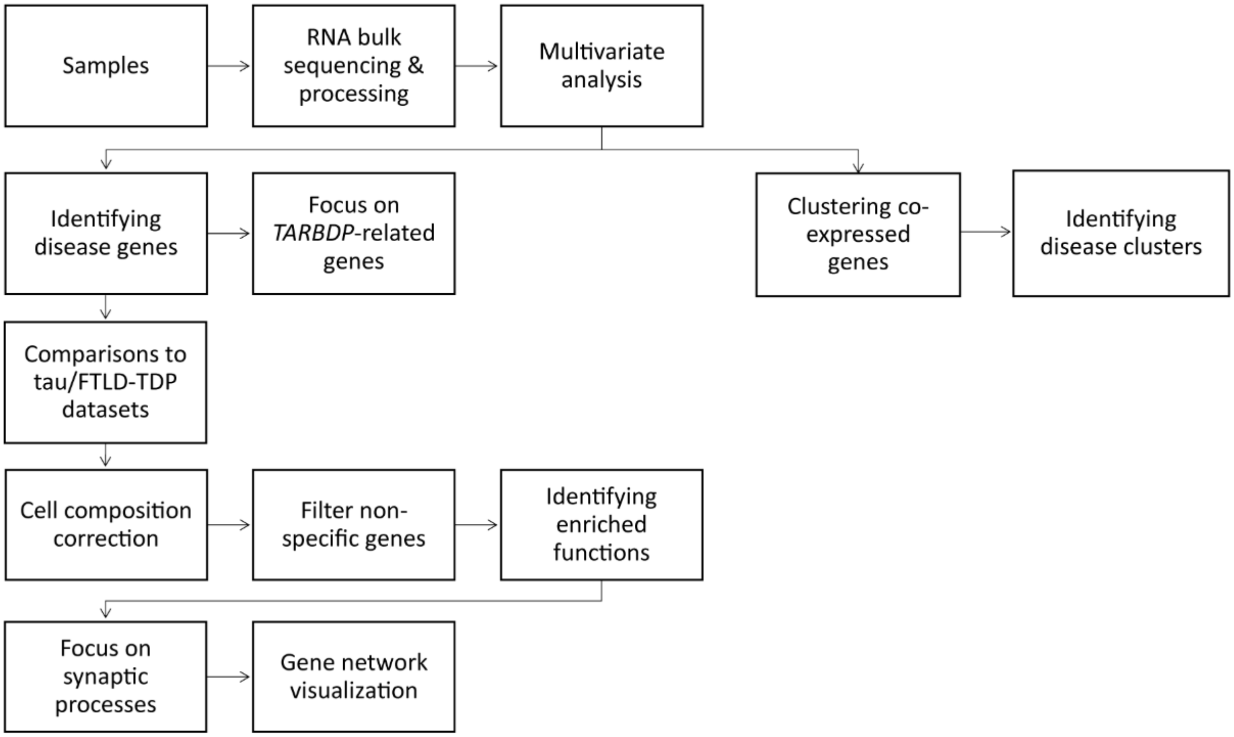

Supplementary Figure 1: Workflow of bulk RNA sequencing analyses steps

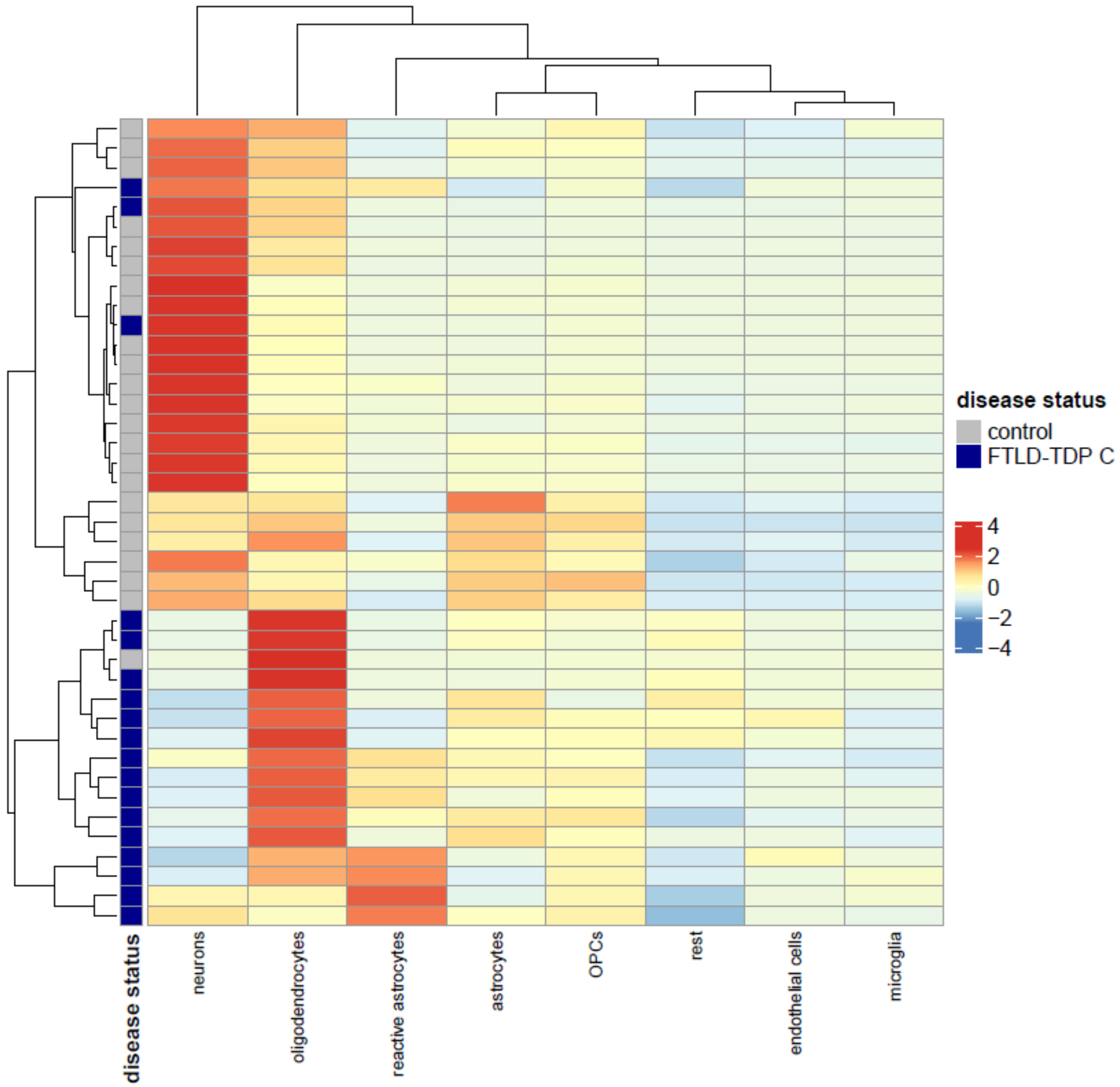

Supplementary Figure 2: Heatmap of effect of cell-type composition on clustering of samples

Samples are clustered in a dendrogram to the left according to cell-type composition. The rows represent the different samples, either FTLD-TDP C or controls, as can be seen on the right y-axis. Expression of seven different cell types is shown in columns with red and blue indicating a higher and lower expression compared to other samples, respectively. Controls are found in the upper rows and have a higher expression of neuronal markers than FTLD-TDP C, whereas FTLD-TDP C samples are found in the lower rows and have a higher expression of oligodendrocyte markers than controls. There is an inverse relationship between the presence of neurons and oligodendrocytes in the samples. Notably, a subgroup of FTLD-TDP C found in the lowest rows has a higher expression of reactive astrocytes compared to other FTLD-TDP C and controls. Other cell types show little to no effect on the clustering of samples according to disease status.

Abbreviations: FTLD-TDP C = frontotemporal lobar degeneration with underlying TDP-43 proteinopathy subtype C, OPCs = Oligodendrocyte precursor cells

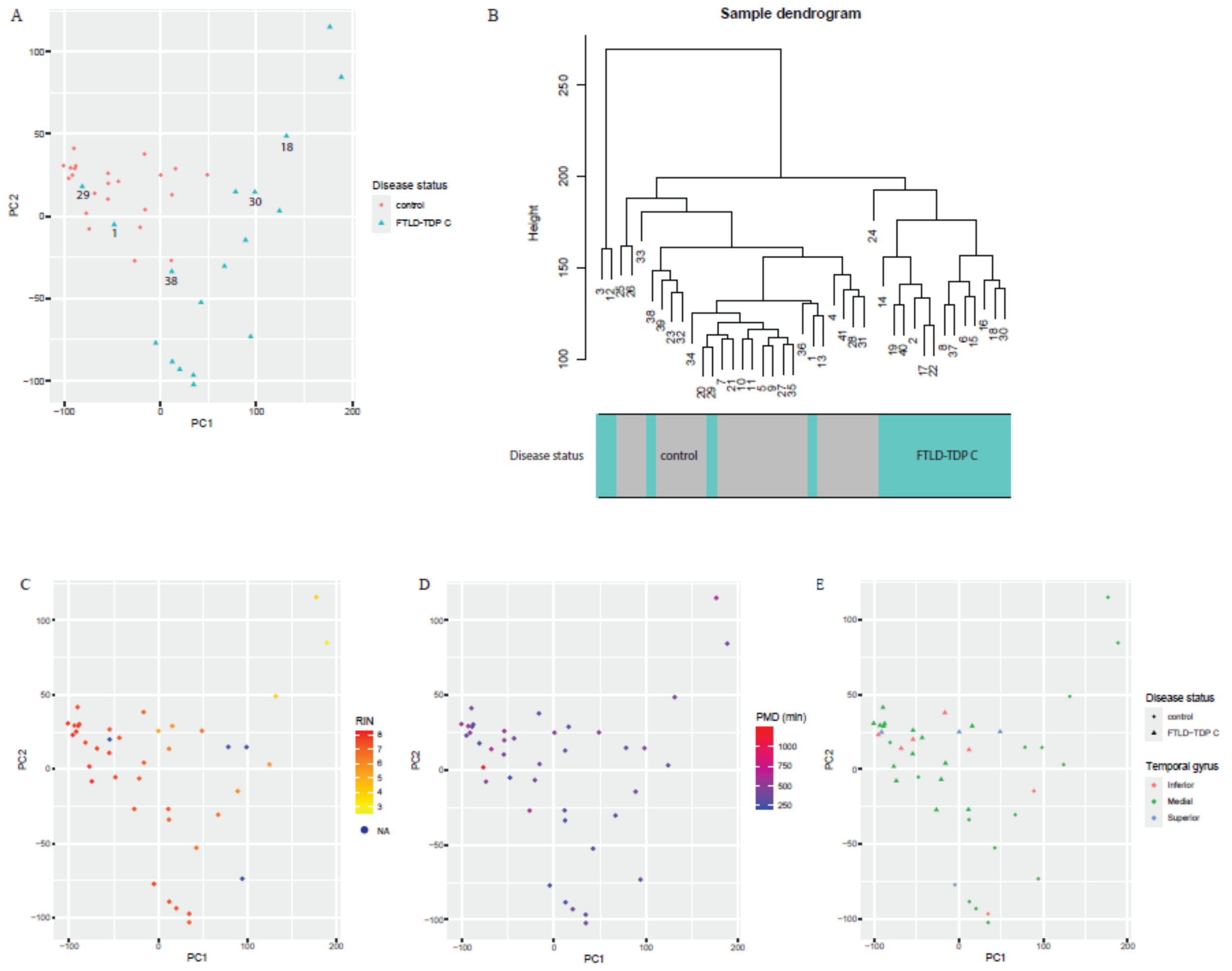

Supplementary Figure 3: Multivariate analyses of FTLD-TDP C and non-demented control samples

(**A**) Samples are plotted on PC1 (x-axis) and 2 (y-axis), showing grouping of non-demented controls (red circles) to the left and FTLD-TDP C (light-blue triangles) to the right, with the exception of two cases found amongst controls (#1, #29). The two cases with somatic variants in *TARDBP* are also shown (#18 with variant in dentate gyrus, #30 variant in medial temporal gyrus). (**B**) Hierarchical clustering analysis shows separate clustering of FTLD-TDP C (turquoise) and non-demented controls (grey), with the exception of three cases clustered among controls (#1, #29, #38). These three cases are grouped with or near controls on the PC plot too. (**C**) Distribution of samples across PC 1 and 2 is partly determined by RIN, with samples with low RIN (yellow) found in the upper right corner and samples with higher RIN (red) spread toward the lower left quarter of the plot. (**D**) Samples across PC 1 and 2 are colour-coded blue to red according to PMD and appear randomly distributed. (**E**) Samples across PC 1 and 2 are colour-labelled according to the sampled subregion of the temporal cortex, with the shape indicating disease status.

Abbreviations: FTLD-TDP C = frontotemporal lobar degeneration with underlying TDP-43 proteinopathy subtype C, PC = principal component, PMD = post-mortem duration, RIN = RNA integrity number.

Supplementary Figure 4: Hierarchical clustering of RNA sequencing datasets of temporal cortex of other dementias

(**A**) Hierarchical clustering analysis of Mayo samples consisting of 82 AD, 84 PSP, 71 PA and 78 NDC, detected two outliers (one PSP, one NDC) which were excluded from further analysis. (**B**) Hierarchical clustering analysis of MSBB samples consisting of 112 AD, 95 MCI and 33 NDC, found no outliers. (**C**) Hierarchical clustering analysis of Edinburgh samples consisting of 12 FTLD-MAPT cases and 21 NDC, showed separate clustering based on disease status. (**D**) Hierarchical clustering of NYGC samples consisting of 20 TDP confirmed cases and 15 NDC, showed scattered and mixed clustering of cases and controls.

Abbreviations: AD = Alzheimer's disease, DE = differentially expressed with FDR < 0.05, FTLD-MAPT = frontotemporal lobar degeneration with underlying microtubule associated protein tau 10+16 deletion, Mayo = Mayo Clinic Brain Bank, MCI = mild cognitive impairment, MSBB = Mount Sinai Brain Bank, NDC = non-demented controls, NYGC = New York Genome Center, PA = pathological aging, PMD = post-mortem delay, PSP = progressive supranuclear palsy, RIN = RNA integrity number, TDP = TAR DNA binding protein-43 pathology

Supplementary Figure 5: GO plots of overrepresented gene ontology terms per module of the weighted gene co-expression network analysis

Per module the overrepresented terms are displayed hierarchically for each of the three categories of gene ontology terms: biological processes, cellular components and molecular functions. Significance is indicated by colour code.

Abbreviations: isa = 'is a' indicating that node A is a subtype of node B.

References

1. Dando O, McGeachan R, McQueen J, et al. Synaptic gene expression changes in frontotemporal dementia due to the MAPT 10 + 16 mutation. *Neuropathol Appl Neurobiol*. 2024;50:e13006.
2. Hasan R, Humphrey J, Bettencourt C, *et al.* Transcriptomic analysis of frontotemporal lobar degeneration with TDP-43 pathology reveals cellular alterations across multiple brain regions. *Acta Neuropathol*. 2022;143(3):383-401.
3. Helmold BR, Pauss KE, Ozdinler PH. TDP-43 protein interactome informs about perturbed canonical pathways and may help develop personalized medicine approaches for patients with TDP-43 pathology. *Drug Discov Today*. 2023;28:103769.
4. Allen Institute for Brain Science. Human MTG SMART-seq Middle Temporal Gyrus. Accessed 20 June 2024. https://portal.brain-map.org/atlases-and-data/rnaseq/human-mtg-smart-seq
5. Sutton GJ, Poppe D, Simmons RK, *et al.* Comprehensive evaluation of deconvolution methods for human brain gene expression. *Nat Commun*. 2022;13:1358.
6. Miedema SSM, Rajicic A, Mol MO, et al. Proteomics of the temporal cortex in semantic dementia reveals brain-region specific molecular pathology and regulation of the TDP-43-ANXA11 interactome. Acta Neuropathol Commun. 2025;13:162.
