# Supplementary material for: Transcriptomic signature of frontotemporal lobar degeneration with TDP-43 type C pathology
Source: Brain. 2026 Mar 6;149(11):3875–89. doi: 10.1093/brain/awag085 (PMC13730574; doi:10.1093/brain/awag085)
Supplement: Supplementary file 1 [file awag085_supplementary_data.zip › brain-2025-02148-File011.pdf]

A

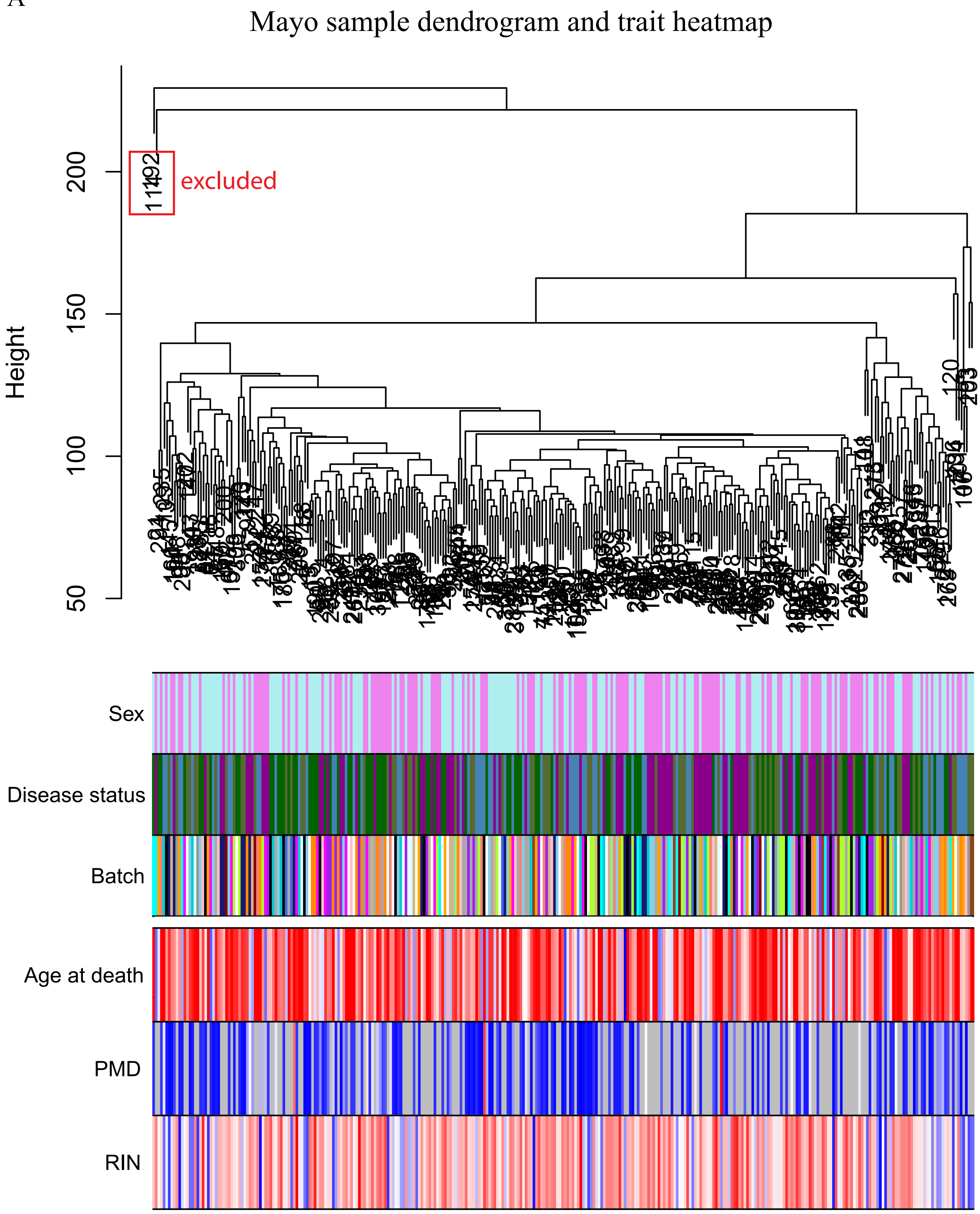

Mayo sample dendrogram and trait heatmap
Height
200
150
100
50
excluded
Sex
Disease status
Batch
Age at death
PMD
RIN

B

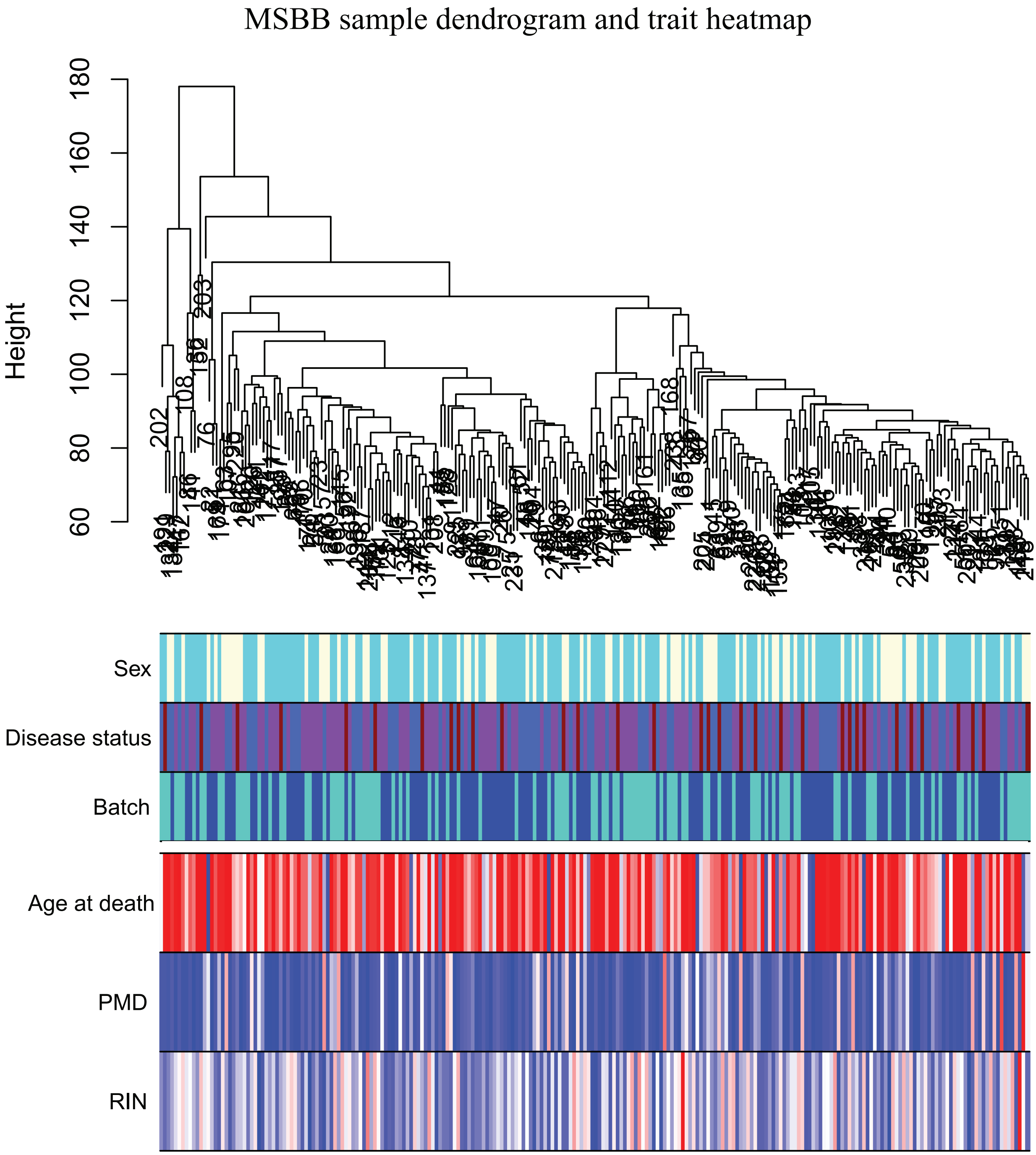
MSBB sample dendrogram and trait heatmap
Height
180
160
140
120
100
80
60
Sex
Disease status
Batch
Age at death
PMD
RIN

C

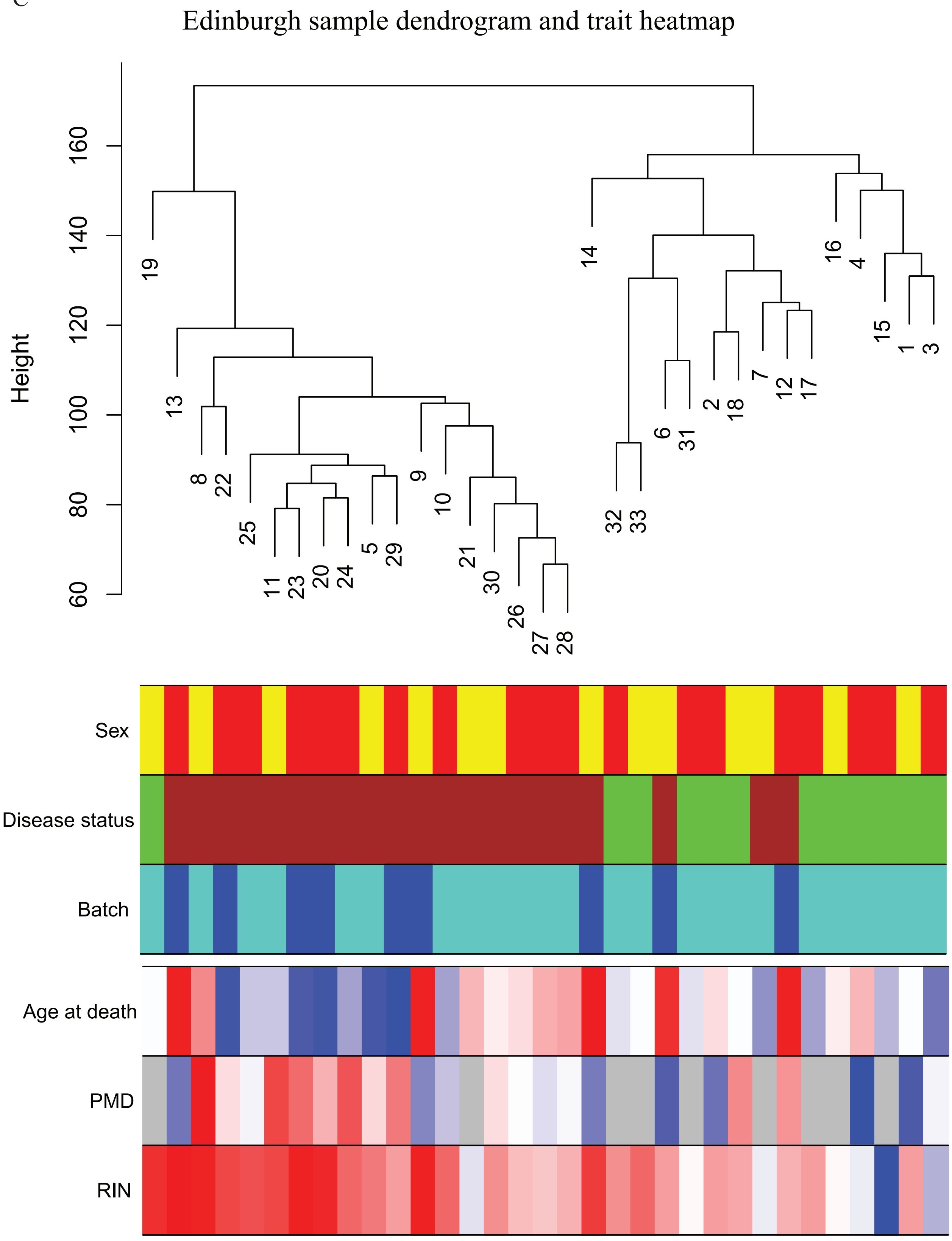
Edinburgh sample dendrogram and trait heatmap
Height
160
140
120
100
80
60
19
13
8
22
25
11
23
20
24
5
29
9
10
21
30
26
27
28
14
32
33
6
31
2
18
7
12
17
16
4
15
1
3
Sex
Disease status
Batch
Age at death
PMD
RIN

D

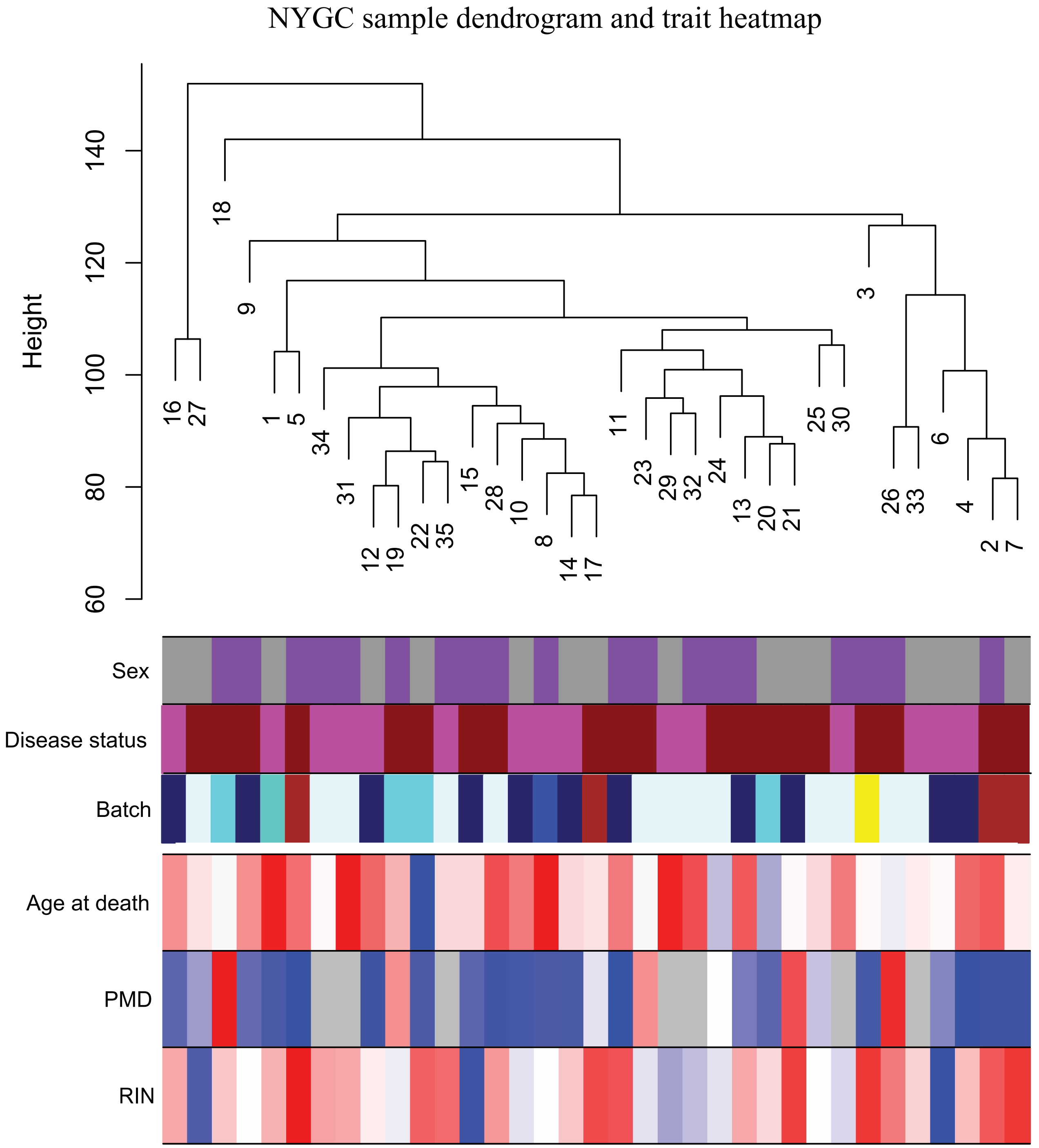
NYGC sample dendrogram and trait heatmap
Height
60
80
100
120
140
16 27 18 1 5 9 34 31 12 19 22 35 15 28 10 8 14 17 11 23 29 32 24 13 20 21 25 30 3 26 33 6 4 2 7
Sex
Disease status
Batch
Age at death
PMD
RIN
