# Supplementary material for: Transcriptomic signature of frontotemporal lobar degeneration with TDP-43 type C pathology
Source: Brain. 2026 Mar 6;149(11):3875–89. doi: 10.1093/brain/awag085 (PMC13730574; doi:10.1093/brain/awag085)

M1 Biological Process

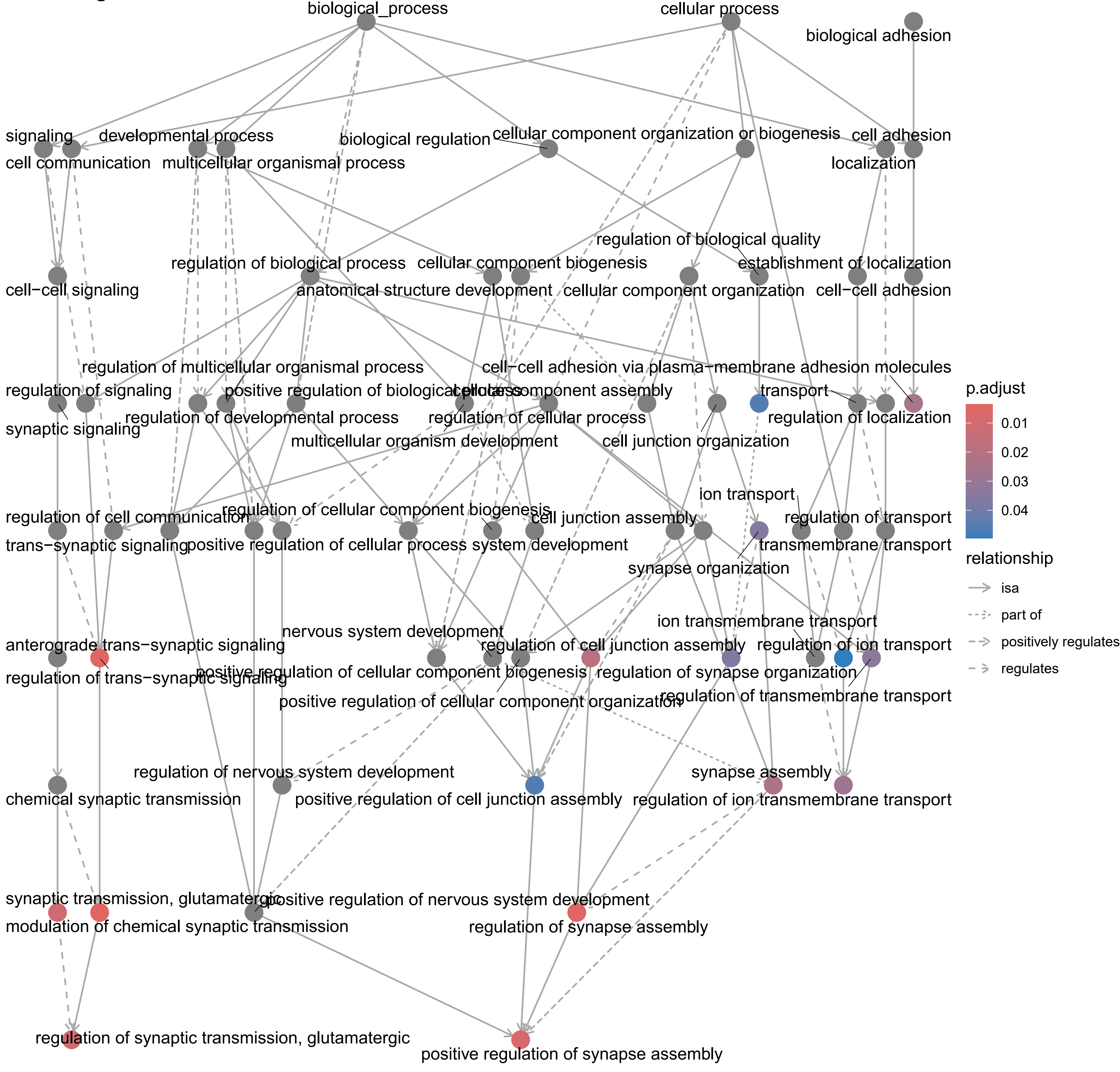

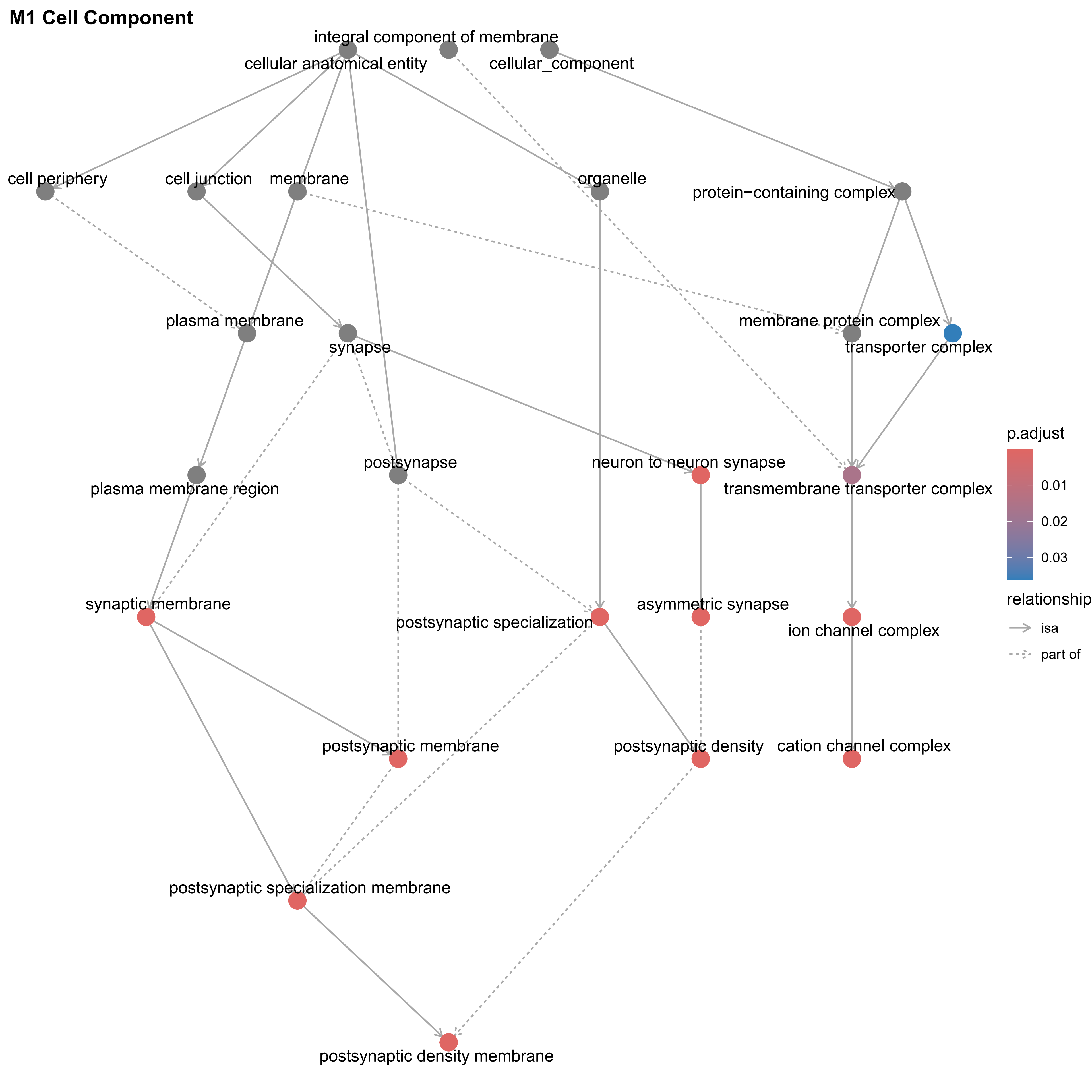
M1 Cell Component
integral component of membrane
cellular anatomical entity
cellular_component
cell periphery
cell junction
membrane
organelle
protein−containing complex
plasma membrane
synapse
membrane protein complex
transporter complex
postsynapse
neuron to neuron synapse
plasma membrane region
transmembrane transporter complex
p.adjust
0.01
0.02
0.03
relationship
isa
part of
synaptic membrane
postsynaptic specialization
asymmetric synapse
ion channel complex
postsynaptic membrane
postsynaptic density
cation channel complex
postsynaptic specialization membrane
postsynaptic density membrane

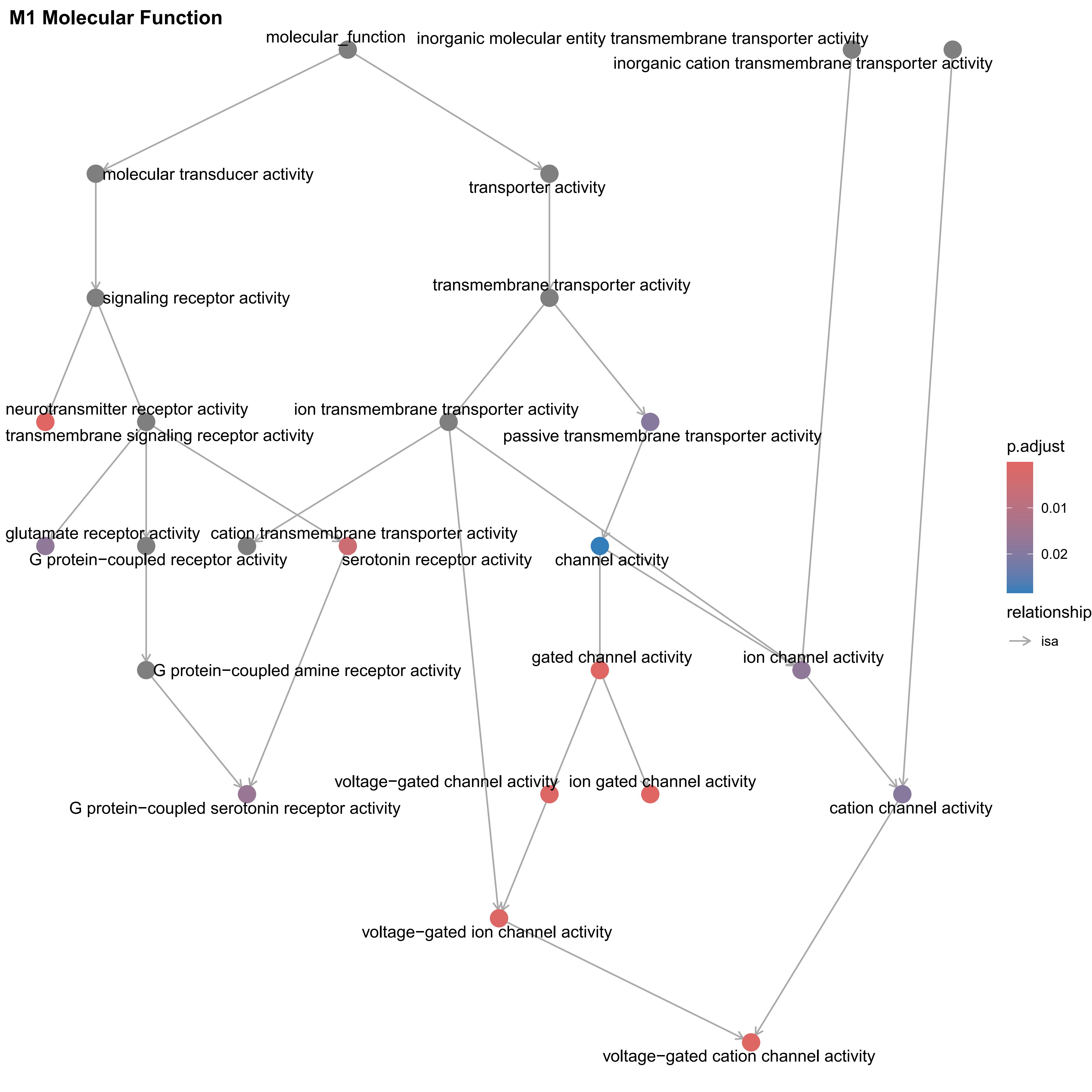
M1 Molecular Function
molecular_function
inorganic molecular entity transmembrane transporter activity
inorganic cation transmembrane transporter activity
molecular transducer activity
transporter activity
signaling receptor activity
transmembrane transporter activity
neurotransmitter receptor activity
transmembrane signaling receptor activity
ion transmembrane transporter activity
passive transmembrane transporter activity
glutamate receptor activity
G protein−coupled receptor activity
cation transmembrane transporter activity
serotonin receptor activity
channel activity
G protein−coupled amine receptor activity
gated channel activity
ion channel activity
voltage−gated channel activity
ion gated channel activity
G protein−coupled serotonin receptor activity
cation channel activity
voltage−gated ion channel activity
voltage−gated cation channel activity
p.adjust
0.01
0.02
relationship
isa

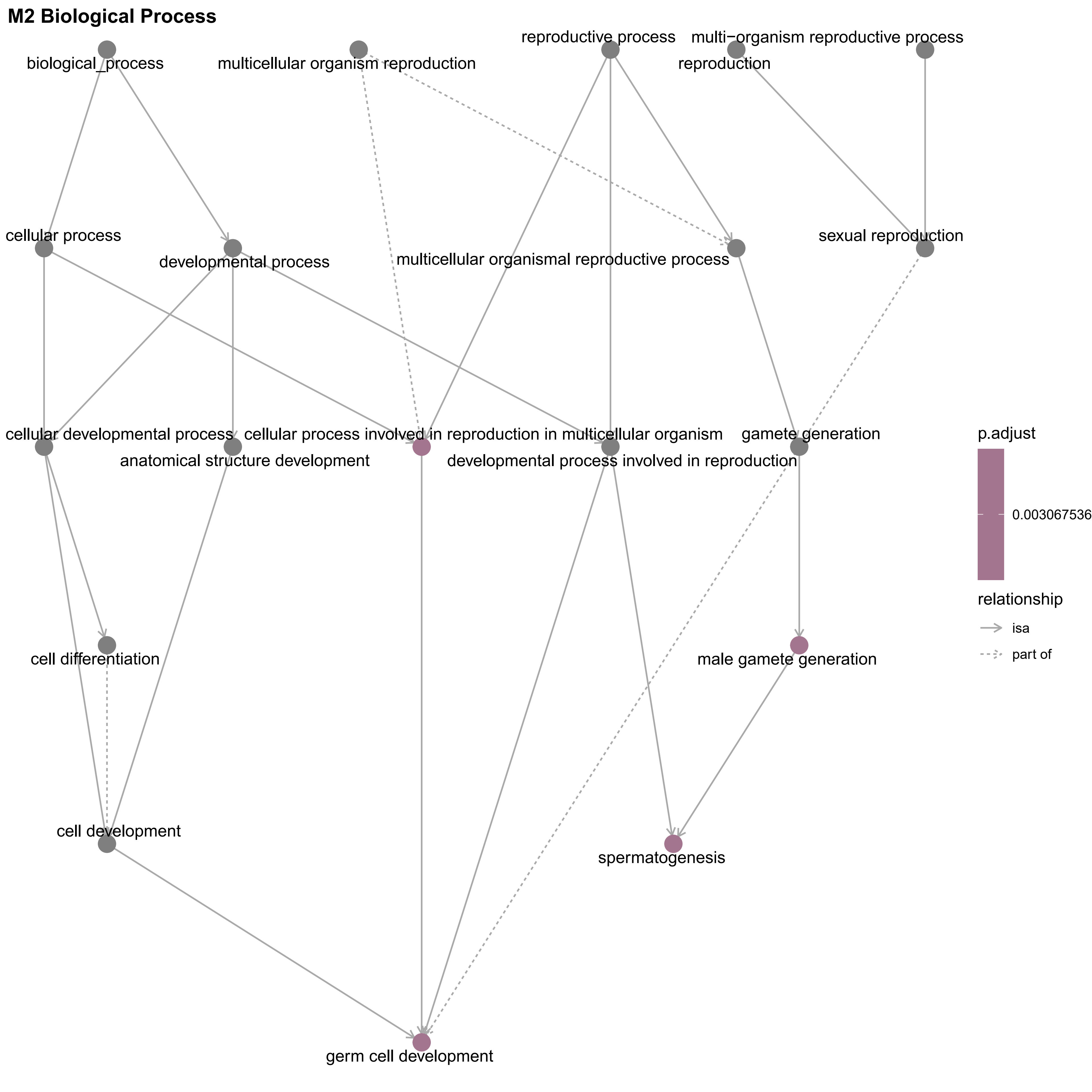
M2 Biological Process
biological_process
multicellular organism reproduction
reproductive process
multi−organism reproductive process
reproduction
cellular process
developmental process
multicellular organismal reproductive process
sexual reproduction
cellular developmental process
cellular process involved in reproduction in multicellular organism
gamete generation
anatomical structure development
developmental process involved in reproduction
cell differentiation
male gamete generation
cell development
spermatogenesis
germ cell development
p.adjust
0.003067536
relationship
isa
part of

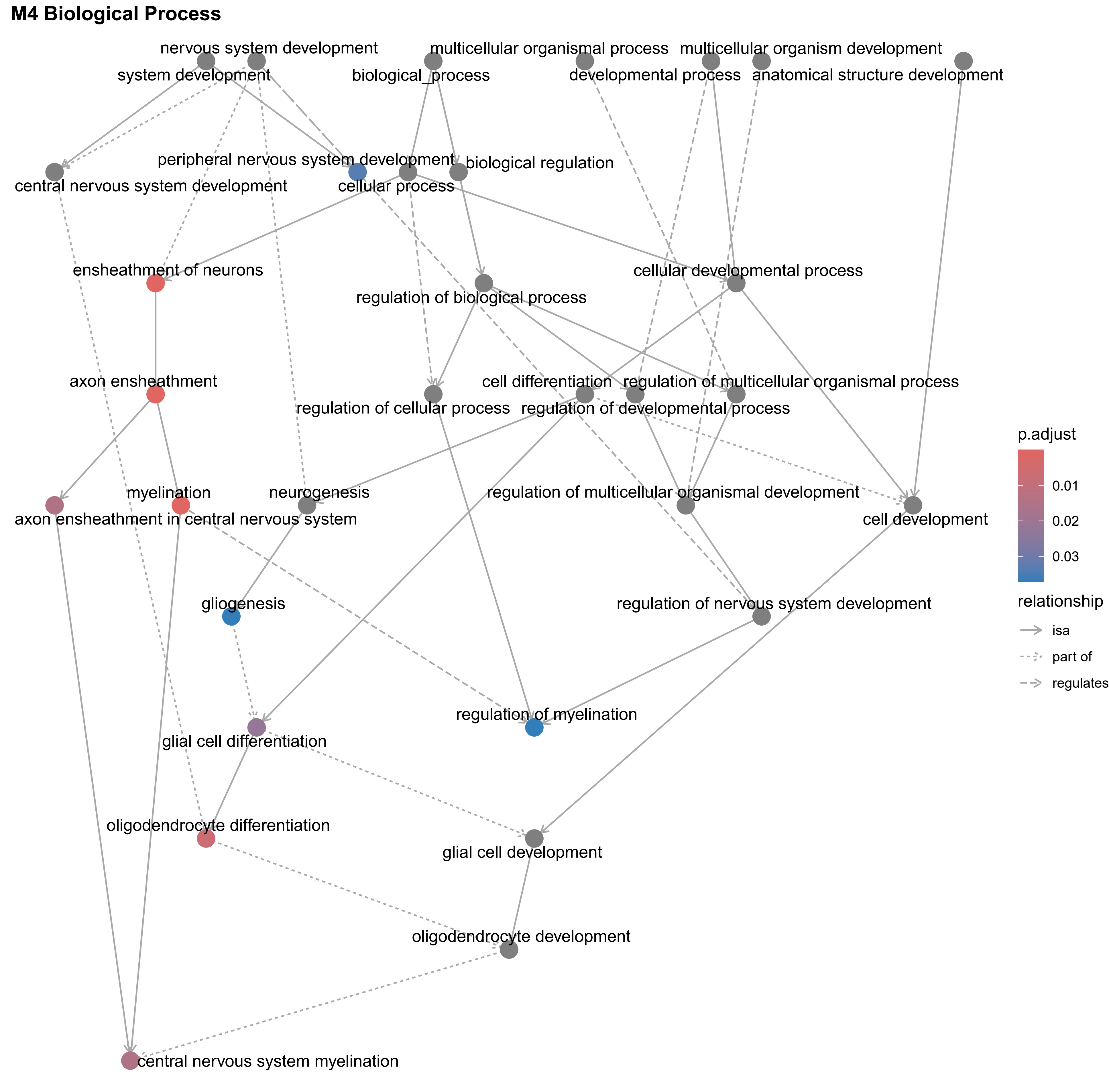
M4 Biological Process
nervous system development
system development
multicellular organismal process
biological_process
multicellular organism development
developmental process
anatomical structure development
peripheral nervous system development
central nervous system development
cellular process
biological regulation
ensheathment of neurons
cellular developmental process
regulation of biological process
axon ensheathment
cell differentiation
regulation of multicellular organismal process
regulation of cellular process
regulation of developmental process
myelination
neurogenesis
regulation of multicellular organismal development
axon ensheathment in central nervous system
cell development
gliogenesis
regulation of nervous system development
regulation of myelination
glial cell differentiation
oligodendrocyte differentiation
glial cell development
oligodendrocyte development
central nervous system myelination
p.adjust
0.01
0.02
0.03
relationship
isa
part of
regulates

M4 Cell Component

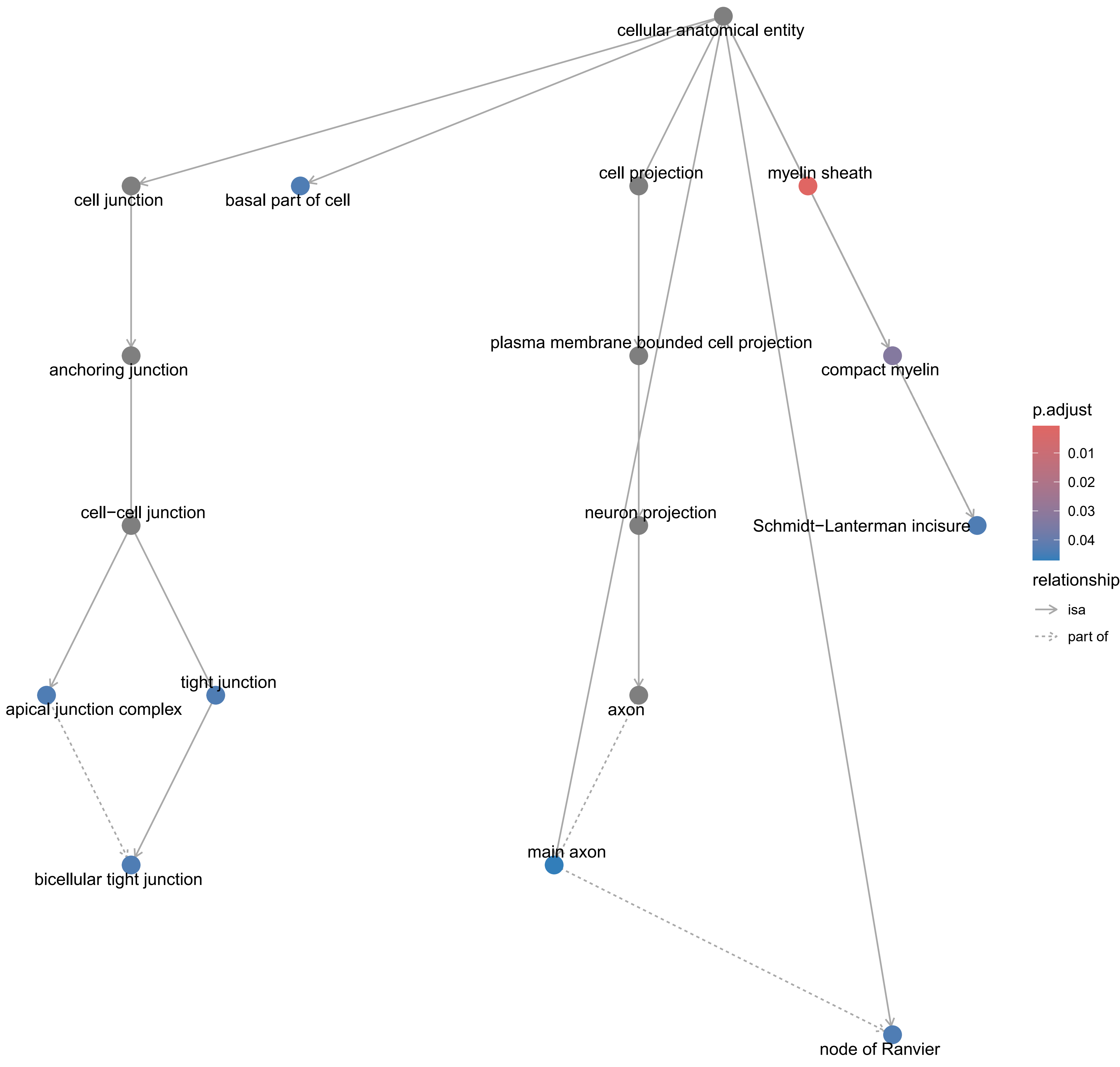

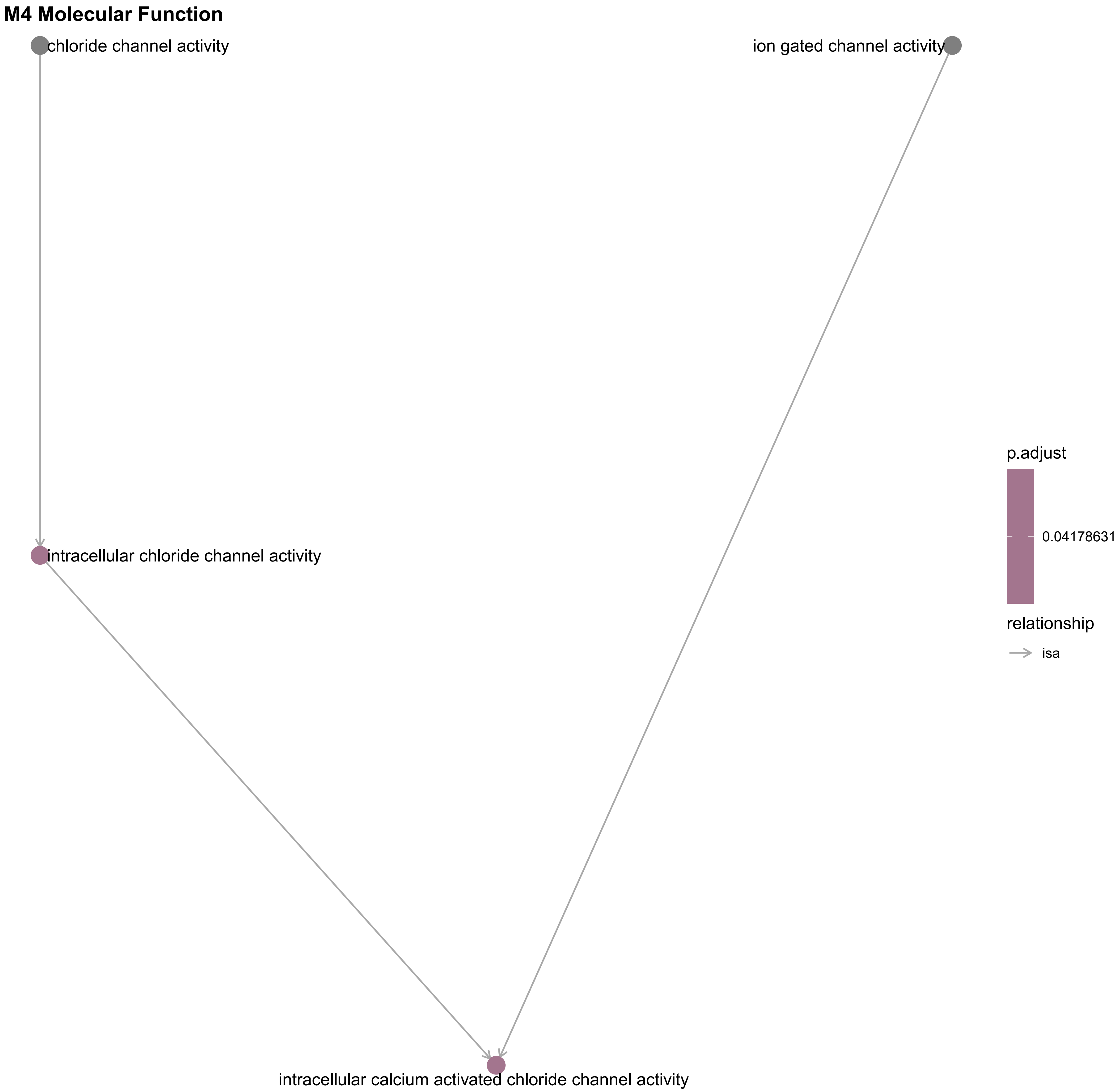
M4 Molecular Function
chloride channel activity
ion gated channel activity
intracellular chloride channel activity
intracellular calcium activated chloride channel activity
p.adjust
0.04178631
relationship
isa

M5 Biological Process

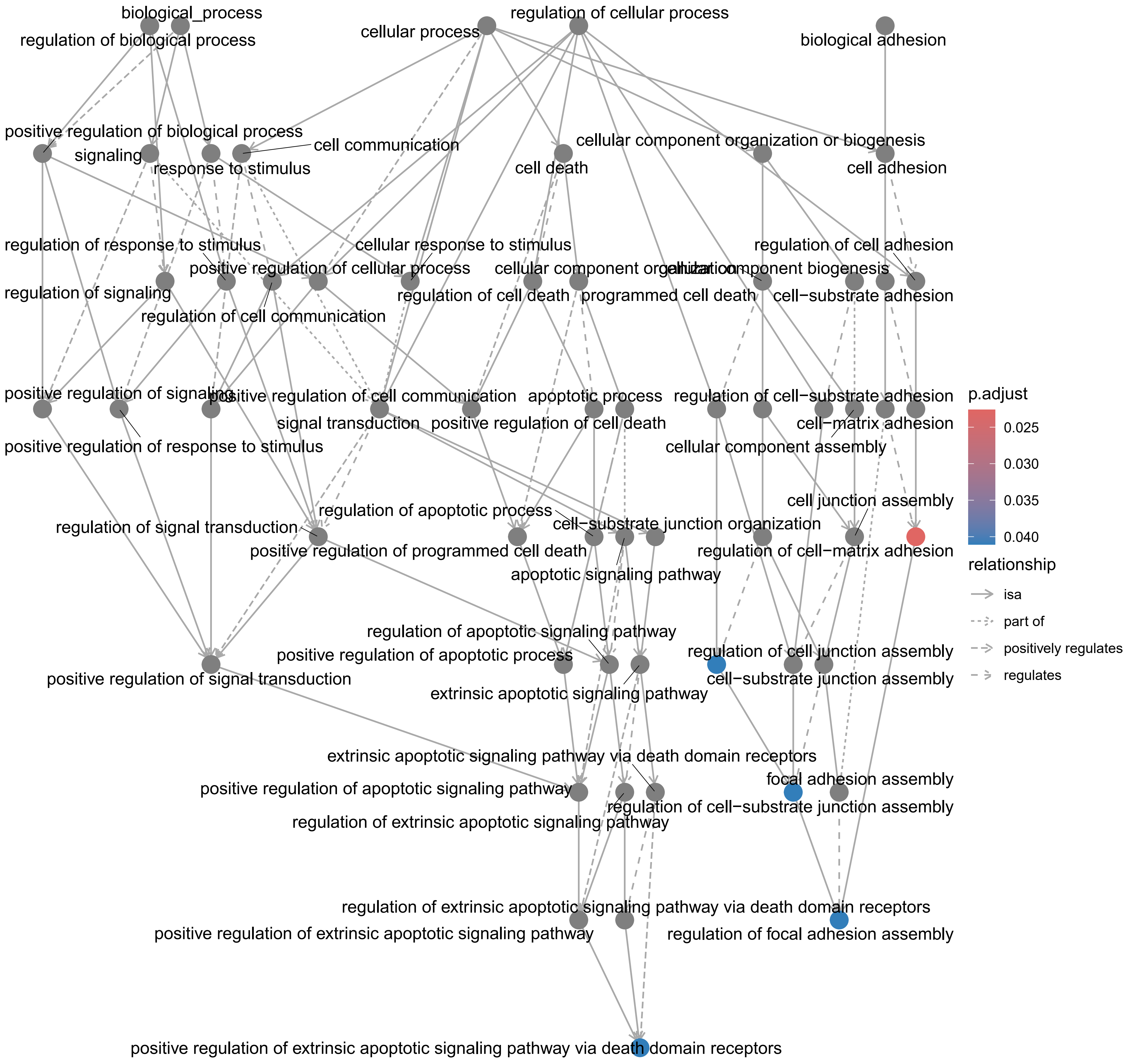

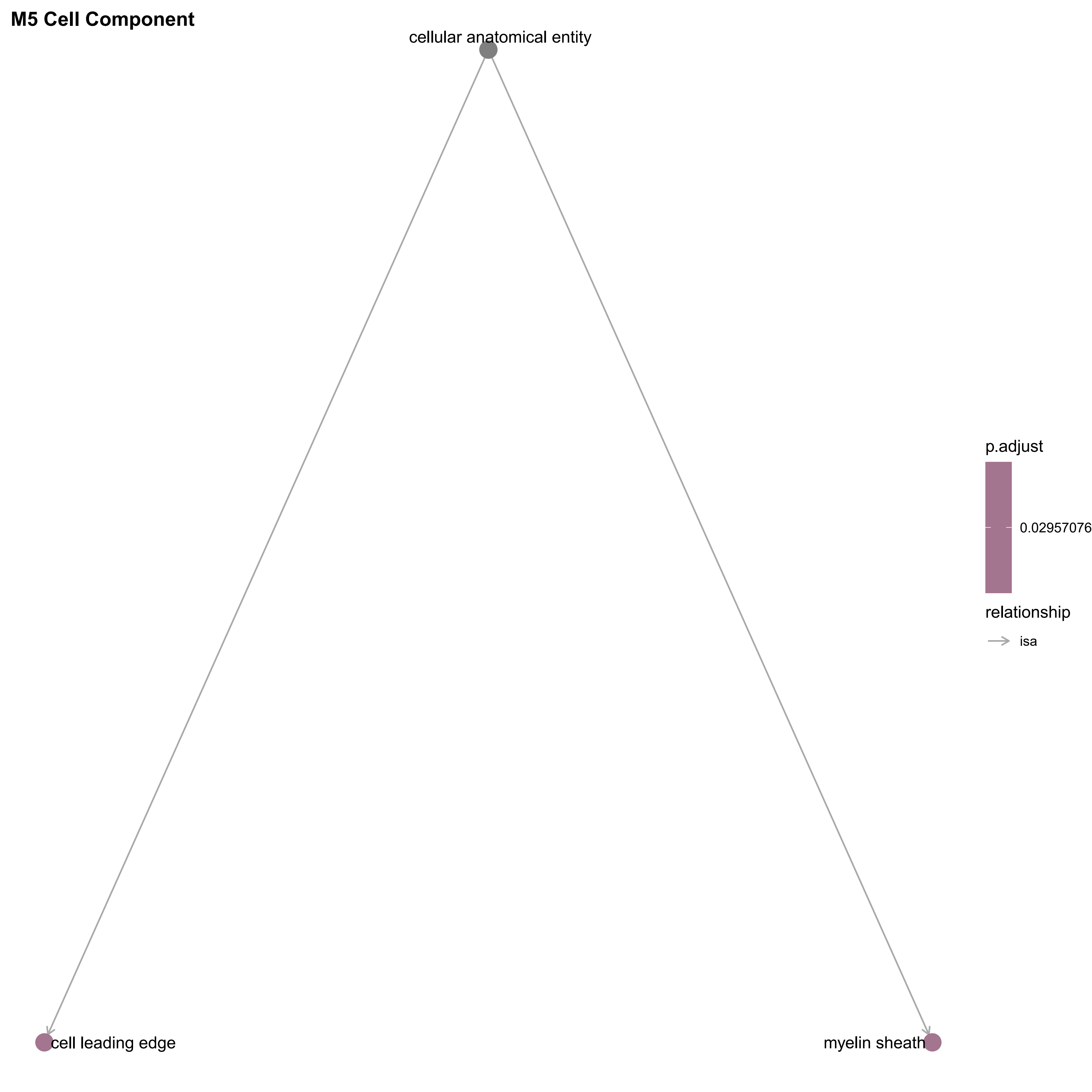
M5 Cell Component
cellular anatomical entity
cell leading edge
myelin sheath
p.adjust
0.02957076
relationship
isa

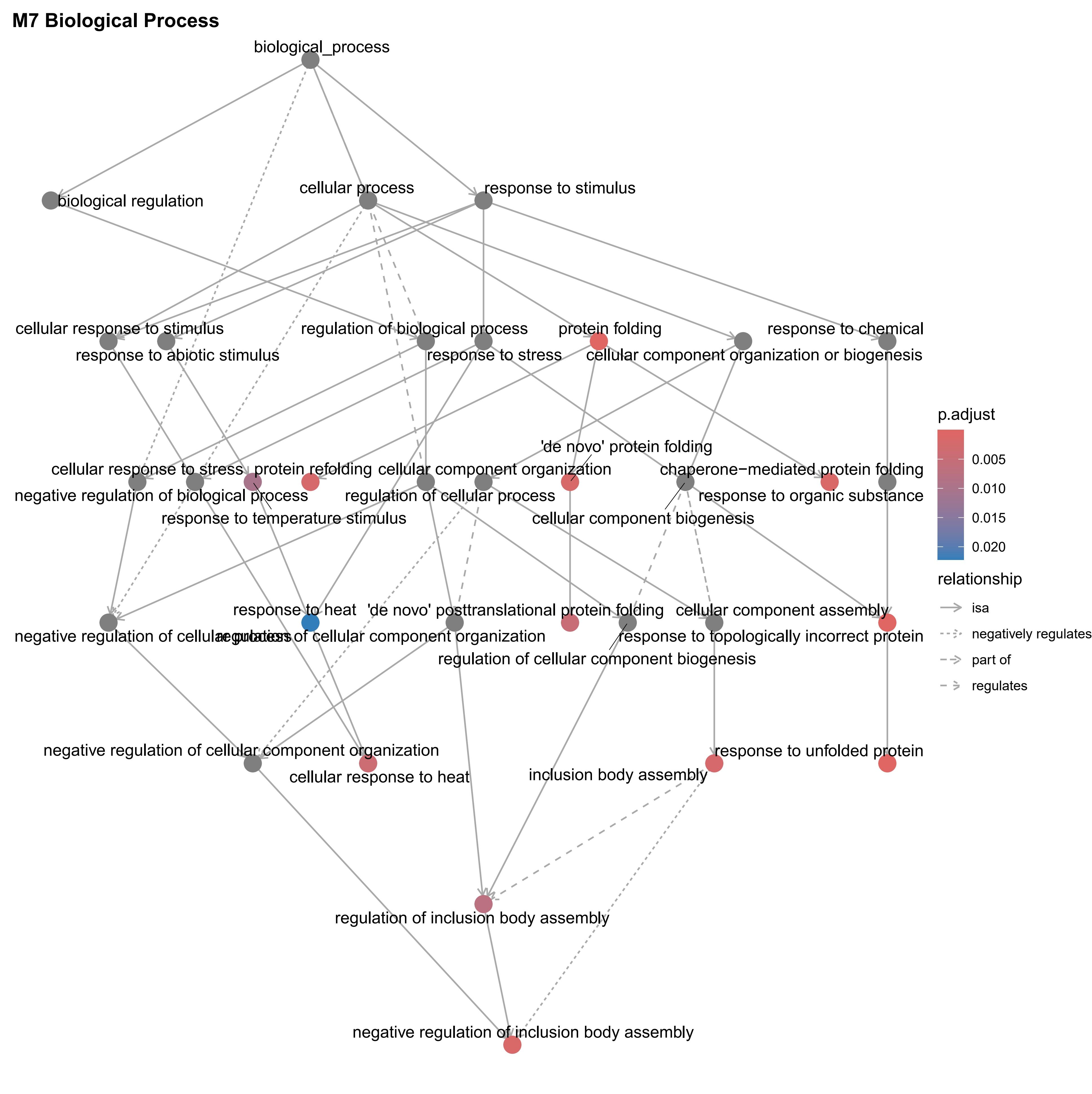

M7 Biological Process
biological_process
biological regulation
cellular process
response to stimulus
cellular response to stimulus
response to abiotic stimulus
regulation of biological process
response to stress
protein folding
cellular component organization or biogenesis
response to chemical
'de novo' protein folding
cellular response to stress
protein refolding
cellular component organization
chaperone−mediated protein folding
negative regulation of biological process
regulation of cellular process
response to organic substance
response to temperature stimulus
cellular component biogenesis
response to heat
'de novo' posttranslational protein folding
cellular component assembly
negative regulation of cellular process
regulation of cellular component organization
response to topologically incorrect protein
regulation of cellular component biogenesis
negative regulation of cellular component organization
response to unfolded protein
cellular response to heat
inclusion body assembly
regulation of inclusion body assembly
negative regulation of inclusion body assembly
p.adjust
0.005
0.010
0.015
0.020
relationship
isa
negatively regulates
part of
regulates

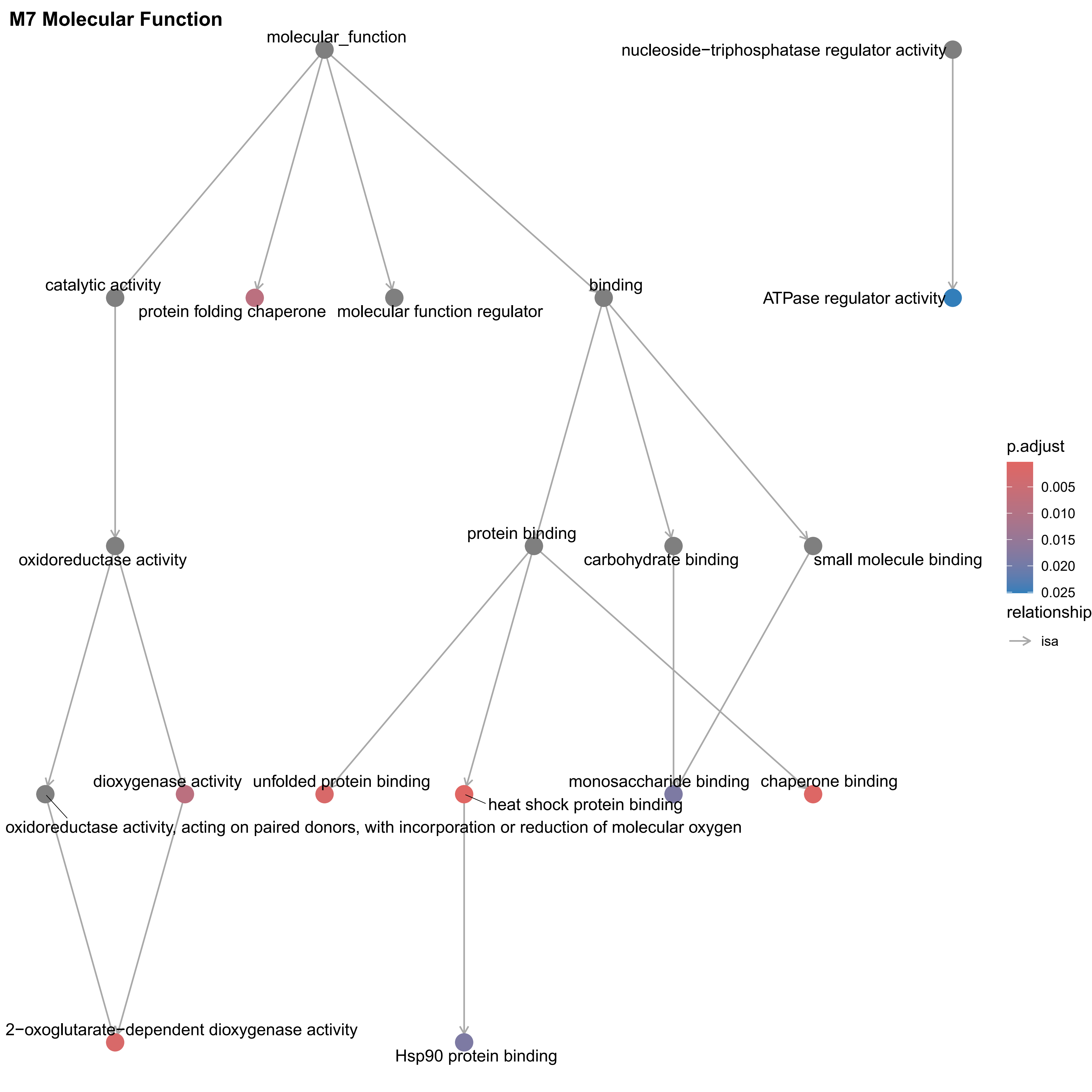
M7 Molecular Function
molecular_function
nucleoside−triphosphatase regulator activity
catalytic activity
protein folding chaperone
molecular function regulator
binding
ATPase regulator activity
p.adjust
0.005
0.010
0.015
0.020
0.025
relationship
isa
oxidoreductase activity
protein binding
carbohydrate binding
small molecule binding
dioxygenase activity
unfolded protein binding
monosaccharide binding
chaperone binding
heat shock protein binding
oxidoreductase activity, acting on paired donors, with incorporation or reduction of molecular oxygen
2−oxoglutarate−dependent dioxygenase activity
Hsp90 protein binding

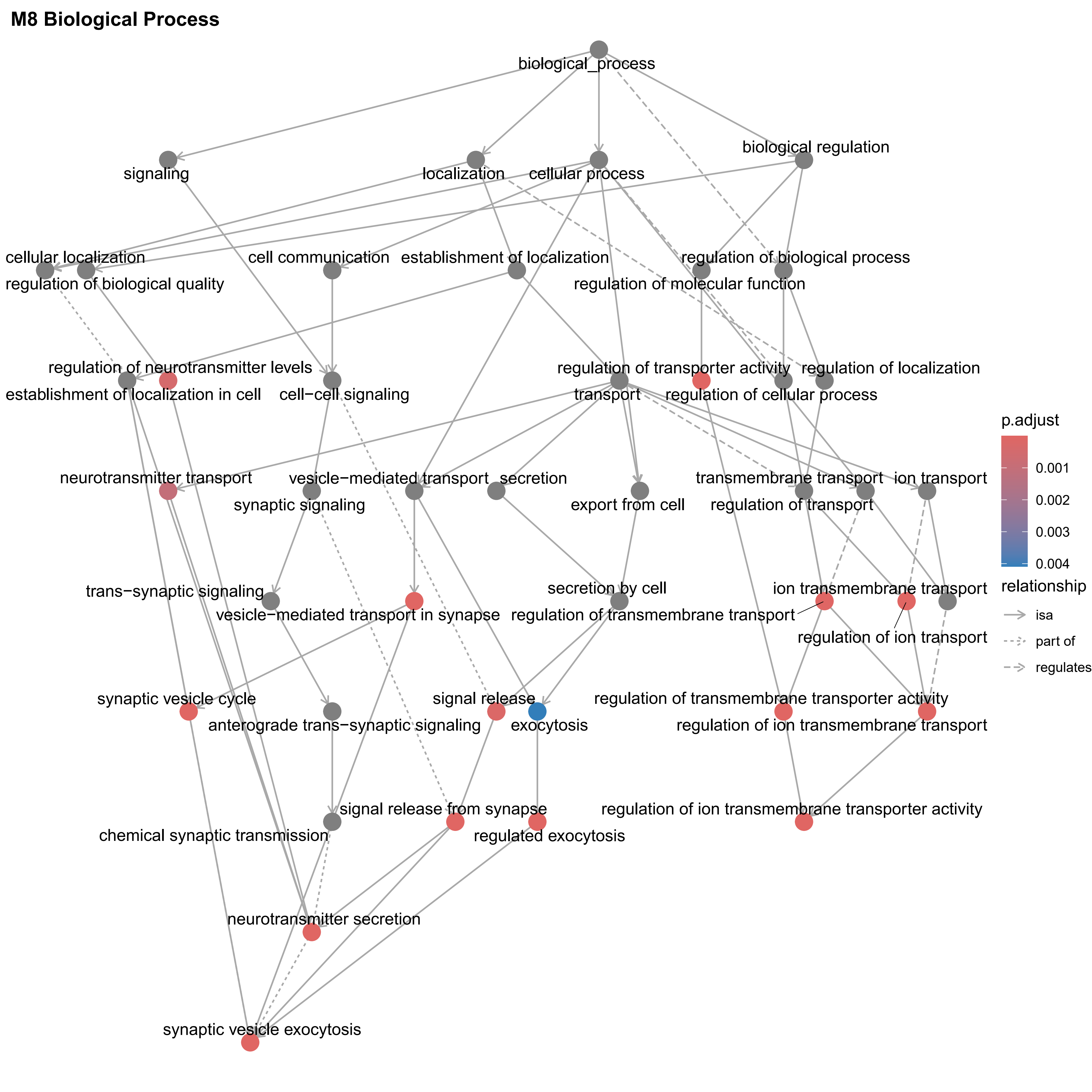
M8 Biological Process
biological_process
signaling
localization
cellular process
biological regulation
cellular localization
regulation of biological quality
cell communication
establishment of localization
regulation of biological process
regulation of molecular function
regulation of neurotransmitter levels
establishment of localization in cell
cell−cell signaling
regulation of transporter activity
transport
regulation of localization
regulation of cellular process
neurotransmitter transport
vesicle−mediated transport
secretion
synaptic signaling
export from cell
transmembrane transport
regulation of transport
ion transport
trans−synaptic signaling
vesicle−mediated transport in synapse
secretion by cell
regulation of transmembrane transport
ion transmembrane transport
regulation of ion transport
synaptic vesicle cycle
anterograde trans−synaptic signaling
signal release
exocytosis
regulation of transmembrane transporter activity
regulation of ion transmembrane transport
signal release from synapse
chemical synaptic transmission
regulated exocytosis
regulation of ion transmembrane transporter activity
neurotransmitter secretion
synaptic vesicle exocytosis
p.adjust
0.001
0.002
0.003
0.004
relationship
isa
part of
regulates

M8 Cell Component

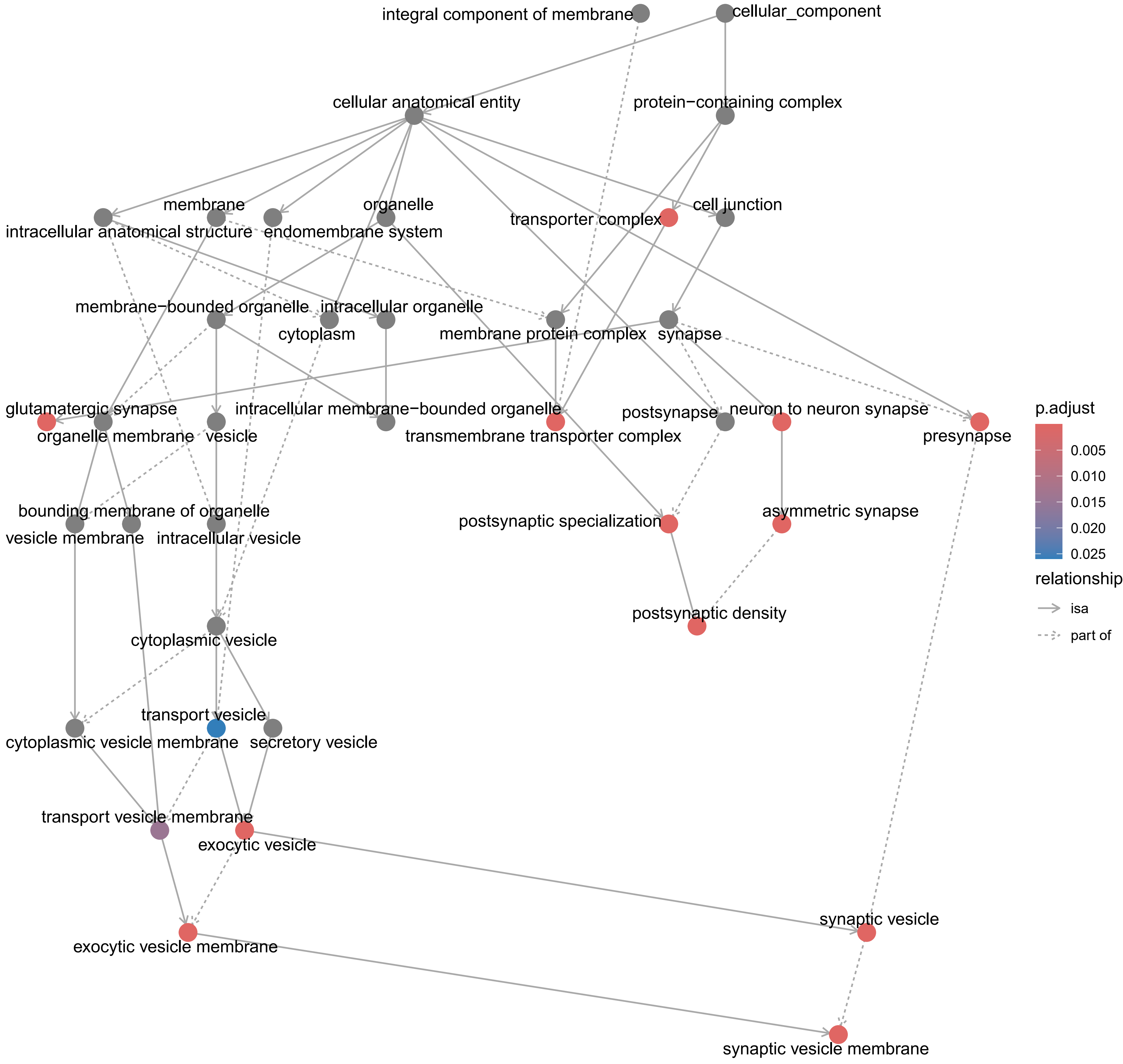

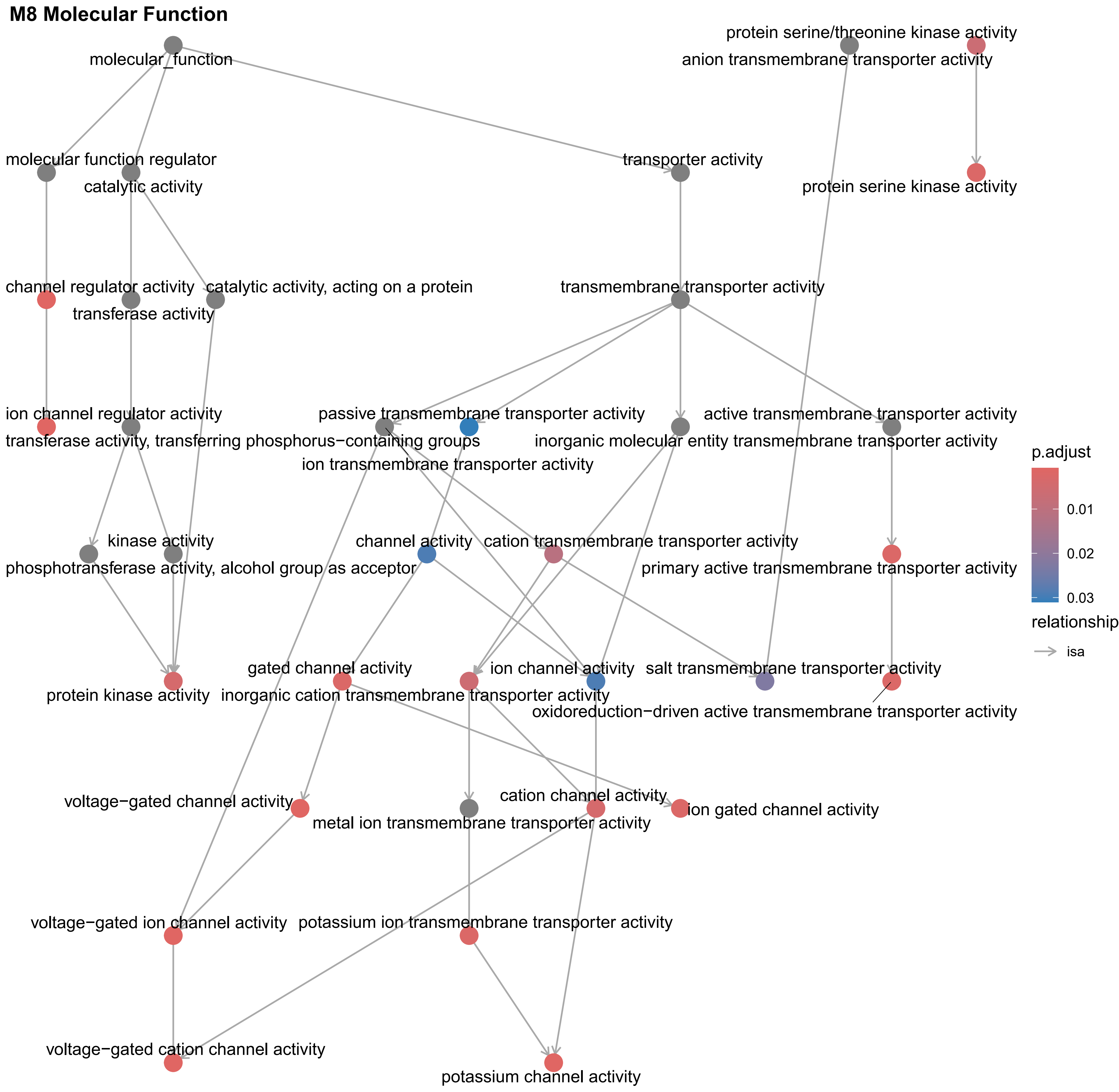
M8 Molecular Function
molecular_function
protein serine/threonine kinase activity
anion transmembrane transporter activity
molecular function regulator
catalytic activity
transporter activity
protein serine kinase activity
channel regulator activity
transferase activity
catalytic activity, acting on a protein
transmembrane transporter activity
ion channel regulator activity
transferase activity, transferring phosphorus−containing groups
passive transmembrane transporter activity
ion transmembrane transporter activity
inorganic molecular entity transmembrane transporter activity
active transmembrane transporter activity
kinase activity
phosphotransferase activity, alcohol group as acceptor
channel activity
cation transmembrane transporter activity
primary active transmembrane transporter activity
protein kinase activity
gated channel activity
inorganic cation transmembrane transporter activity
ion channel activity
salt transmembrane transporter activity
oxidoreduction−driven active transmembrane transporter activity
voltage−gated channel activity
metal ion transmembrane transporter activity
cation channel activity
ion gated channel activity
voltage−gated ion channel activity
potassium ion transmembrane transporter activity
voltage−gated cation channel activity
potassium channel activity
p.adjust
0.01
0.02
0.03
relationship
isa

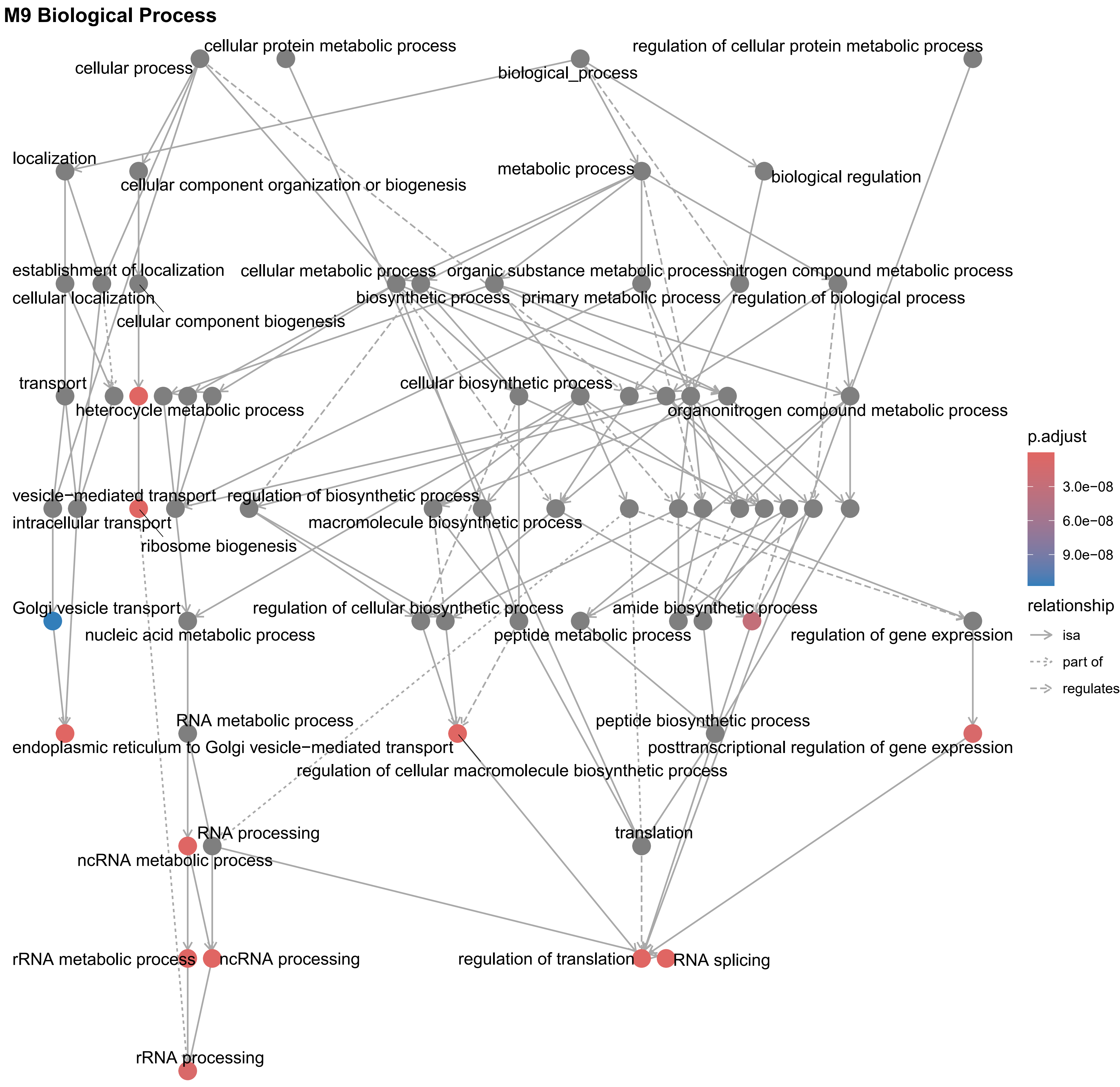
M9 Biological Process
cellular protein metabolic process
regulation of cellular protein metabolic process
cellular process
biological_process
localization
cellular component organization or biogenesis
metabolic process
biological regulation
establishment of localization
cellular metabolic process
organic substance metabolic process
nitrogen compound metabolic process
cellular localization
biosynthetic process
primary metabolic process
regulation of biological process
cellular component biogenesis
transport
cellular biosynthetic process
heterocycle metabolic process
organonitrogen compound metabolic process
vesicle−mediated transport
regulation of biosynthetic process
intracellular transport
macromolecule biosynthetic process
ribosome biogenesis
Golgi vesicle transport
regulation of cellular biosynthetic process
amide biosynthetic process
nucleic acid metabolic process
peptide metabolic process
regulation of gene expression
RNA metabolic process
peptide biosynthetic process
endoplasmic reticulum to Golgi vesicle−mediated transport
posttranscriptional regulation of gene expression
regulation of cellular macromolecule biosynthetic process
RNA processing
translation
ncRNA metabolic process
rRNA metabolic process
ncRNA processing
regulation of translation
RNA splicing
rRNA processing
p.adjust
3.0e−08
6.0e−08
9.0e−08
relationship
isa
part of
regulates

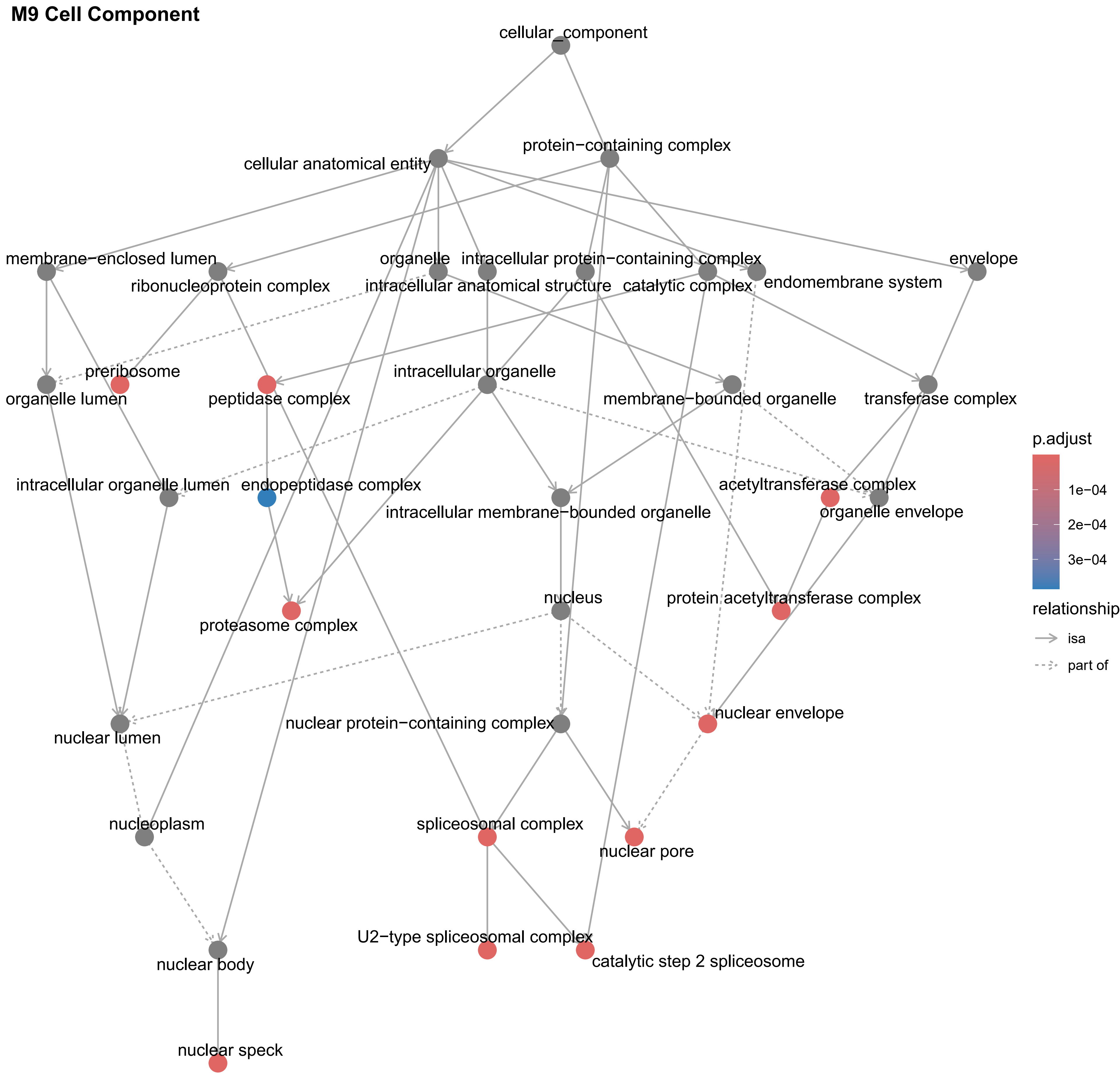

M9 Cell Component
cellular_component
protein−containing complex
cellular anatomical entity
membrane−enclosed lumen
ribonucleoprotein complex
organelle
intracellular anatomical structure
intracellular protein−containing complex
catalytic complex
endomembrane system
envelope
preribosome
organelle lumen
peptidase complex
intracellular organelle
membrane−bounded organelle
transferase complex
intracellular organelle lumen
endopeptidase complex
intracellular membrane−bounded organelle
acetyltransferase complex
organelle envelope
nucleus
proteasome complex
protein acetyltransferase complex
nuclear protein−containing complex
nuclear lumen
nuclear envelope
nucleoplasm
spliceosomal complex
nuclear pore
U2−type spliceosomal complex
catalytic step 2 spliceosome
nuclear body
nuclear speck
p.adjust
1e−04
2e−04
3e−04
relationship
isa
part of

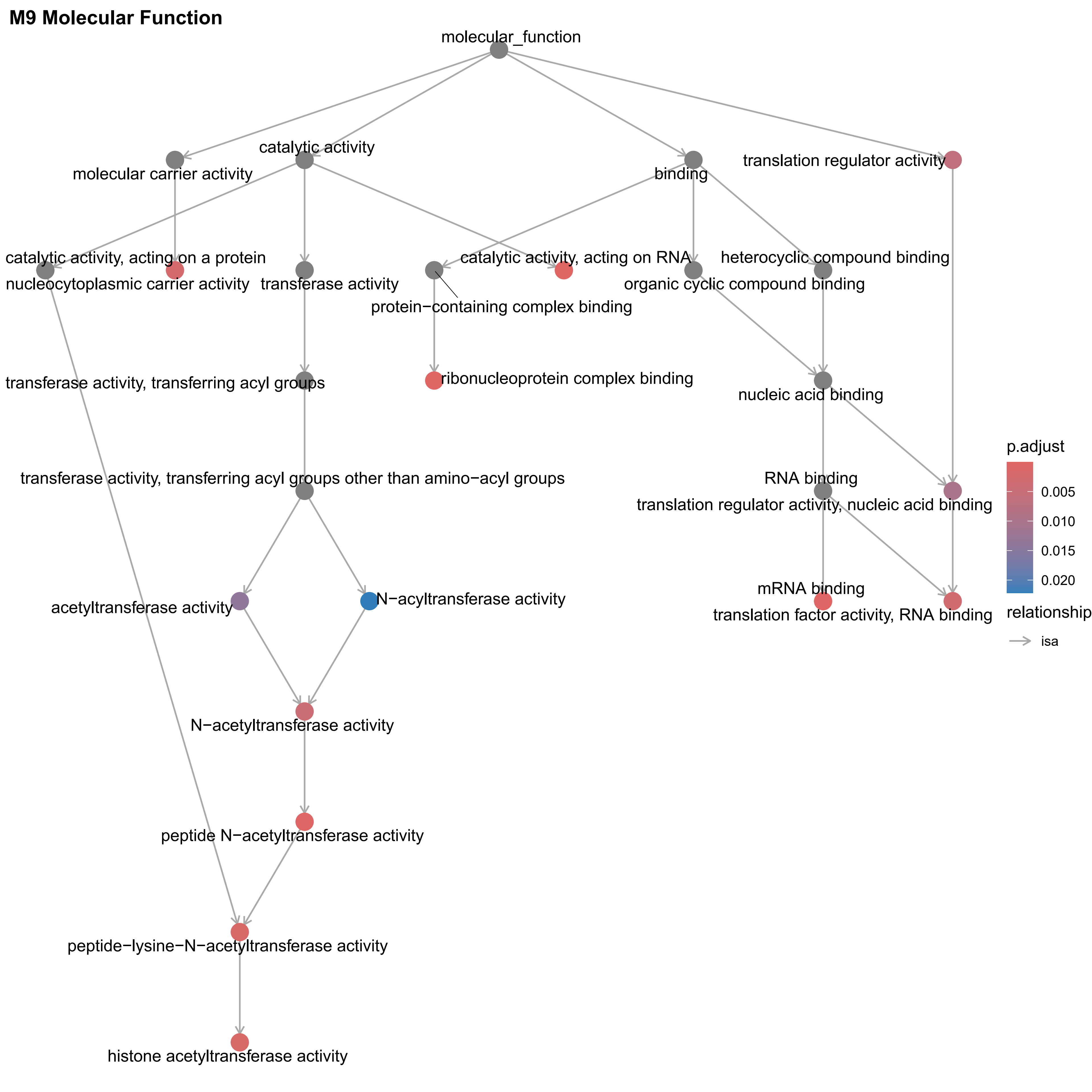
M9 Molecular Function
molecular_function
catalytic activity
molecular carrier activity
binding
translation regulator activity
catalytic activity, acting on a protein
nucleocytoplasmic carrier activity
transferase activity
catalytic activity, acting on RNA
protein−containing complex binding
heterocyclic compound binding
organic cyclic compound binding
transferase activity, transferring acyl groups
ribonucleoprotein complex binding
nucleic acid binding
transferase activity, transferring acyl groups other than amino−acyl groups
RNA binding
translation regulator activity, nucleic acid binding
acetyltransferase activity
N−acyltransferase activity
mRNA binding
translation factor activity, RNA binding
N−acetyltransferase activity
peptide N−acetyltransferase activity
peptide−lysine−N−acetyltransferase activity
histone acetyltransferase activity
p.adjust
0.005
0.010
0.015
0.020
relationship
isa

M10 Biological Process

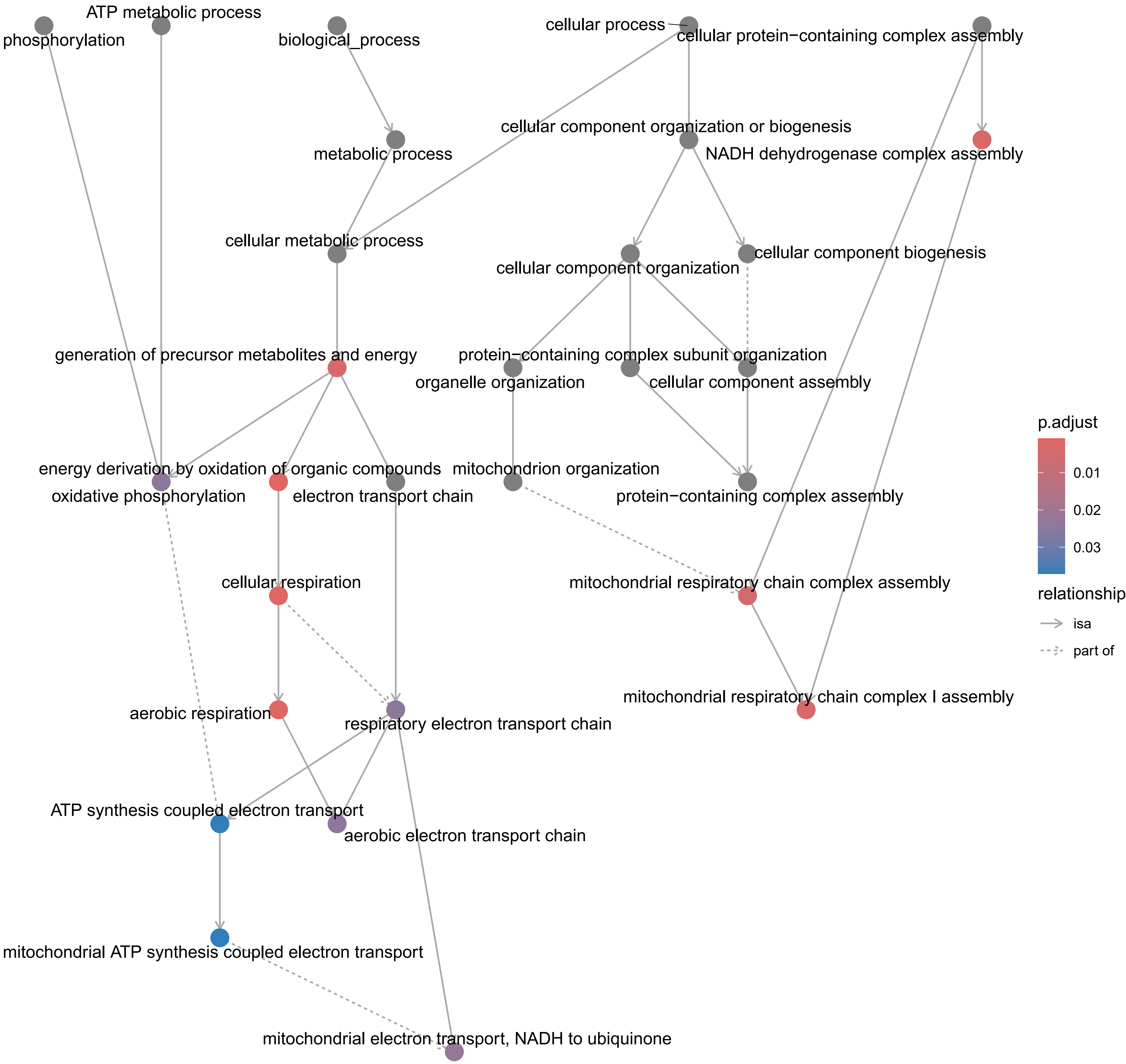

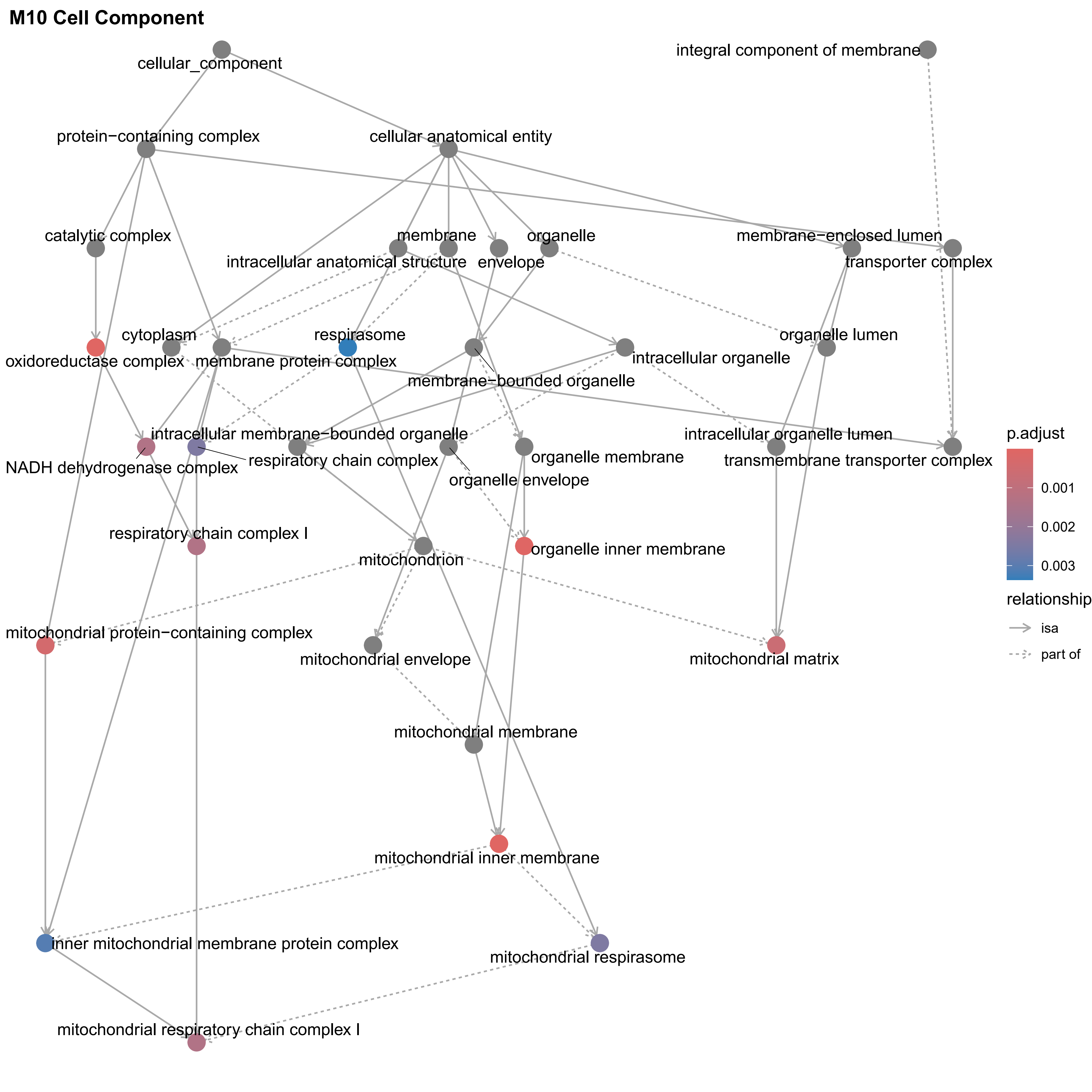
M10 Cell Component
cellular_component
integral component of membrane
protein−containing complex
cellular anatomical entity
catalytic complex
membrane
organelle
membrane−enclosed lumen
intracellular anatomical structure
envelope
transporter complex
cytoplasm
respirasome
organelle lumen
oxidoreductase complex
membrane protein complex
intracellular organelle
membrane−bounded organelle
intracellular membrane−bounded organelle
intracellular organelle lumen
NADH dehydrogenase complex
respiratory chain complex
organelle membrane
transmembrane transporter complex
organelle envelope
respiratory chain complex I
organelle inner membrane
mitochondrion
mitochondrial protein−containing complex
mitochondrial envelope
mitochondrial matrix
mitochondrial membrane
mitochondrial inner membrane
inner mitochondrial membrane protein complex
mitochondrial respirasome
mitochondrial respiratory chain complex I
p.adjust
0.001
0.002
0.003
relationship
isa
part of

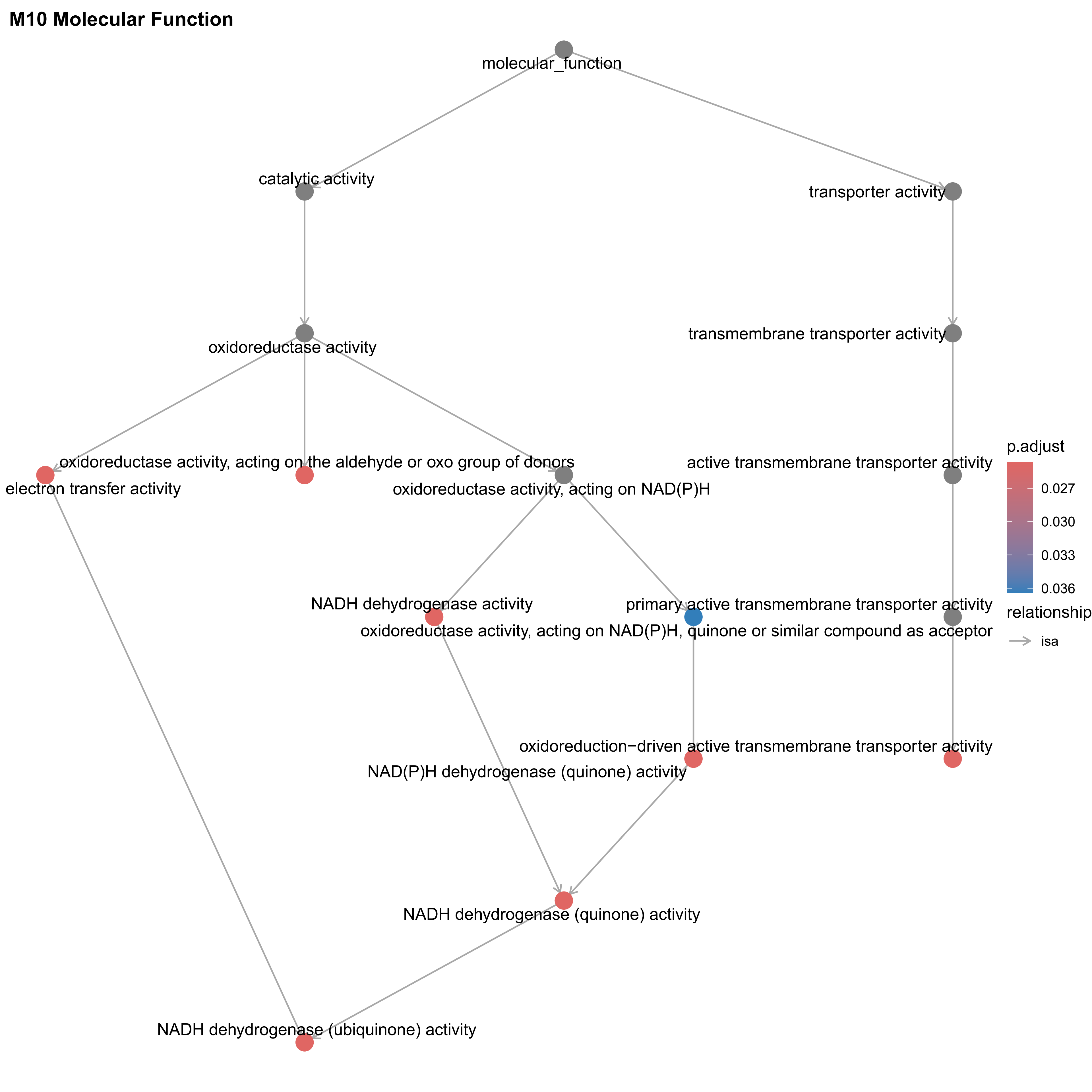
M10 Molecular Function
molecular_function
catalytic activity
transporter activity
oxidoreductase activity
transmembrane transporter activity
oxidoreductase activity, acting on the aldehyde or oxo group of donors
active transmembrane transporter activity
electron transfer activity
oxidoreductase activity, acting on NAD(P)H
NADH dehydrogenase activity
primary active transmembrane transporter activity
oxidoreductase activity, acting on NAD(P)H, quinone or similar compound as acceptor
oxidoreduction−driven active transmembrane transporter activity
NAD(P)H dehydrogenase (quinone) activity
NADH dehydrogenase (quinone) activity
NADH dehydrogenase (ubiquinone) activity
p.adjust
0.027
0.030
0.033
0.036
relationship
isa

M11 Biological Process

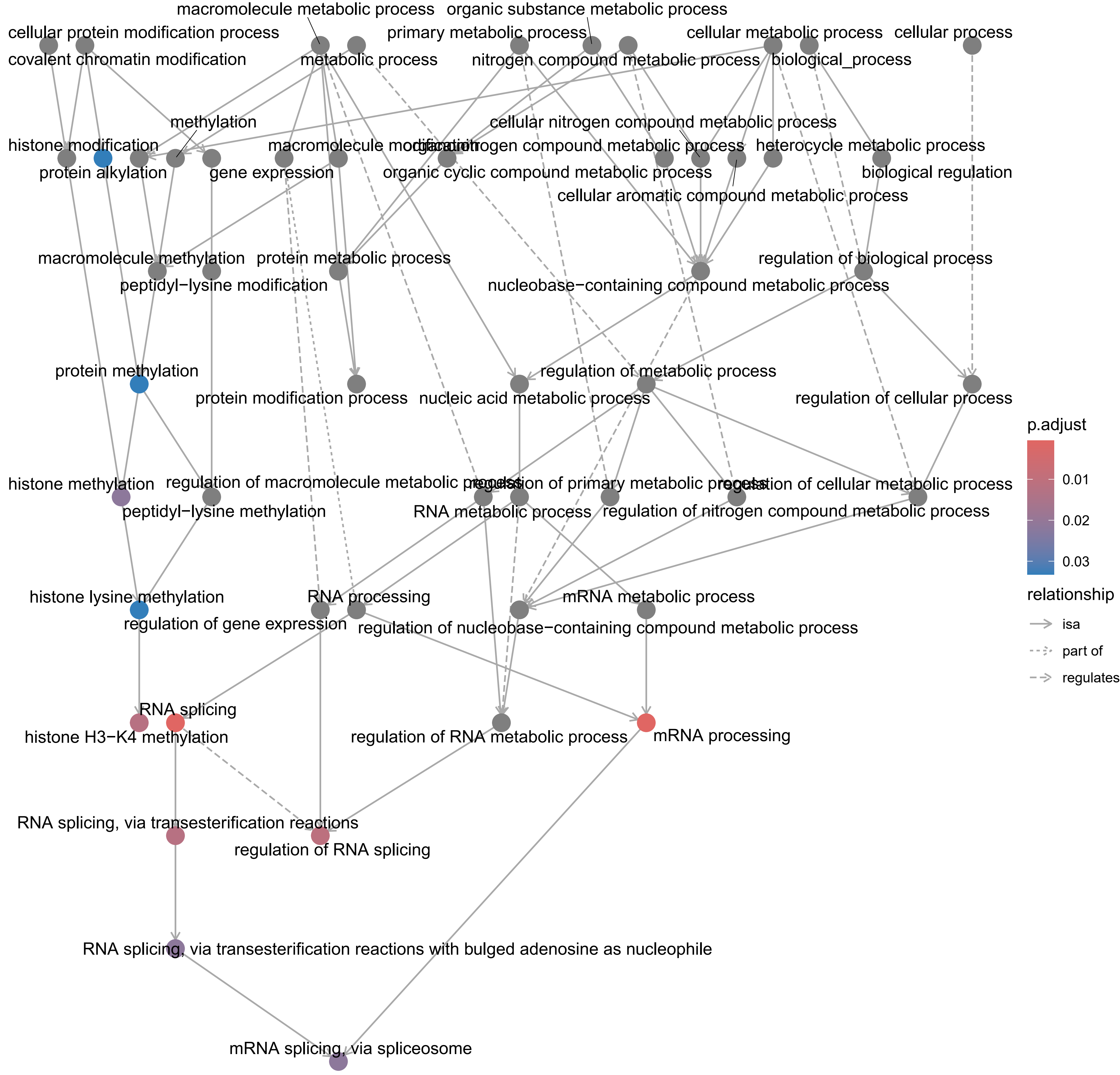

M12 Biological Process

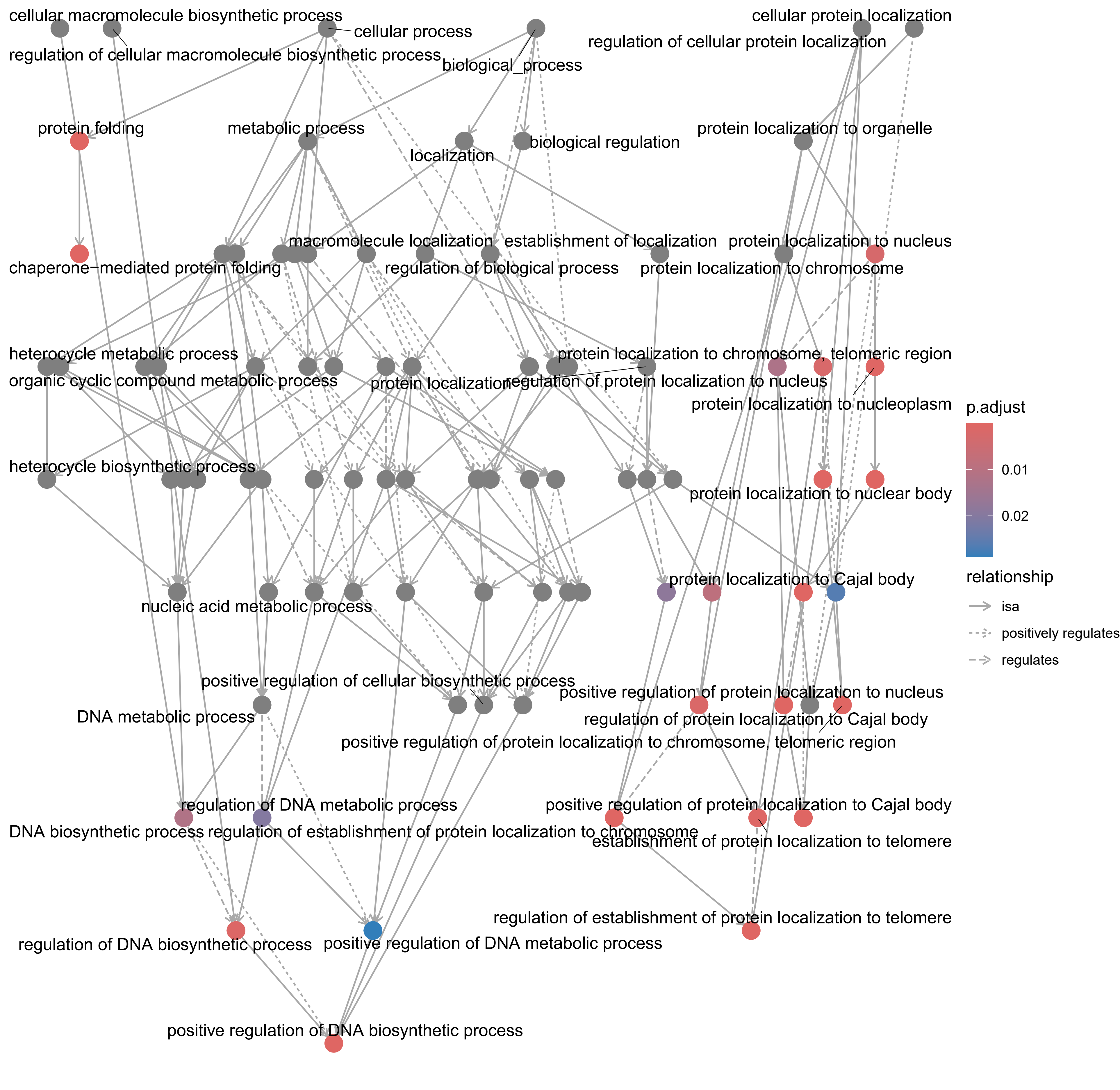

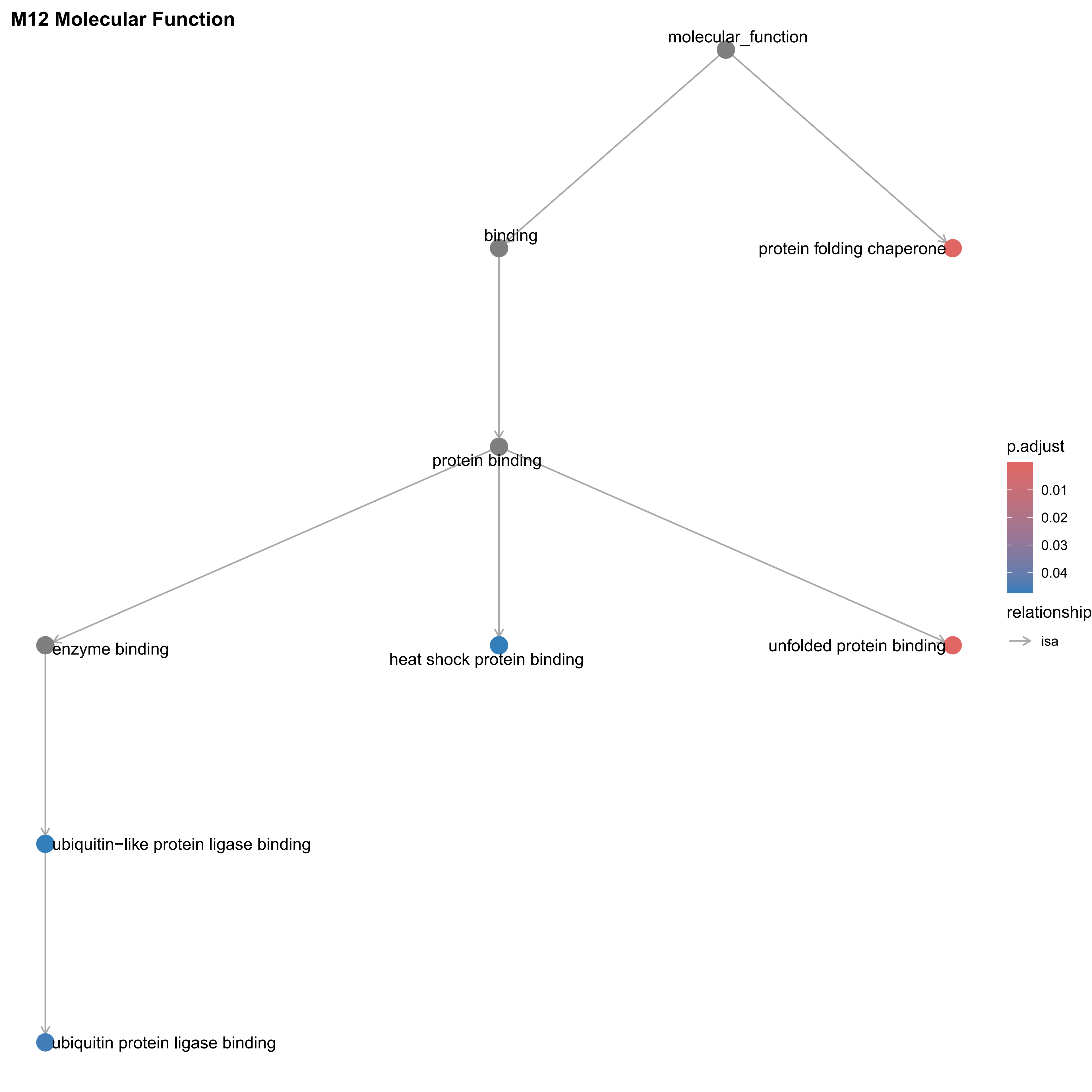
M12 Molecular Function
molecular_function
binding
protein folding chaperone
protein binding
enzyme binding
heat shock protein binding
unfolded protein binding
ubiquitin−like protein ligase binding
ubiquitin protein ligase binding
p.adjust
0.01
0.02
0.03
0.04
relationship
isa

M13 Biological Process

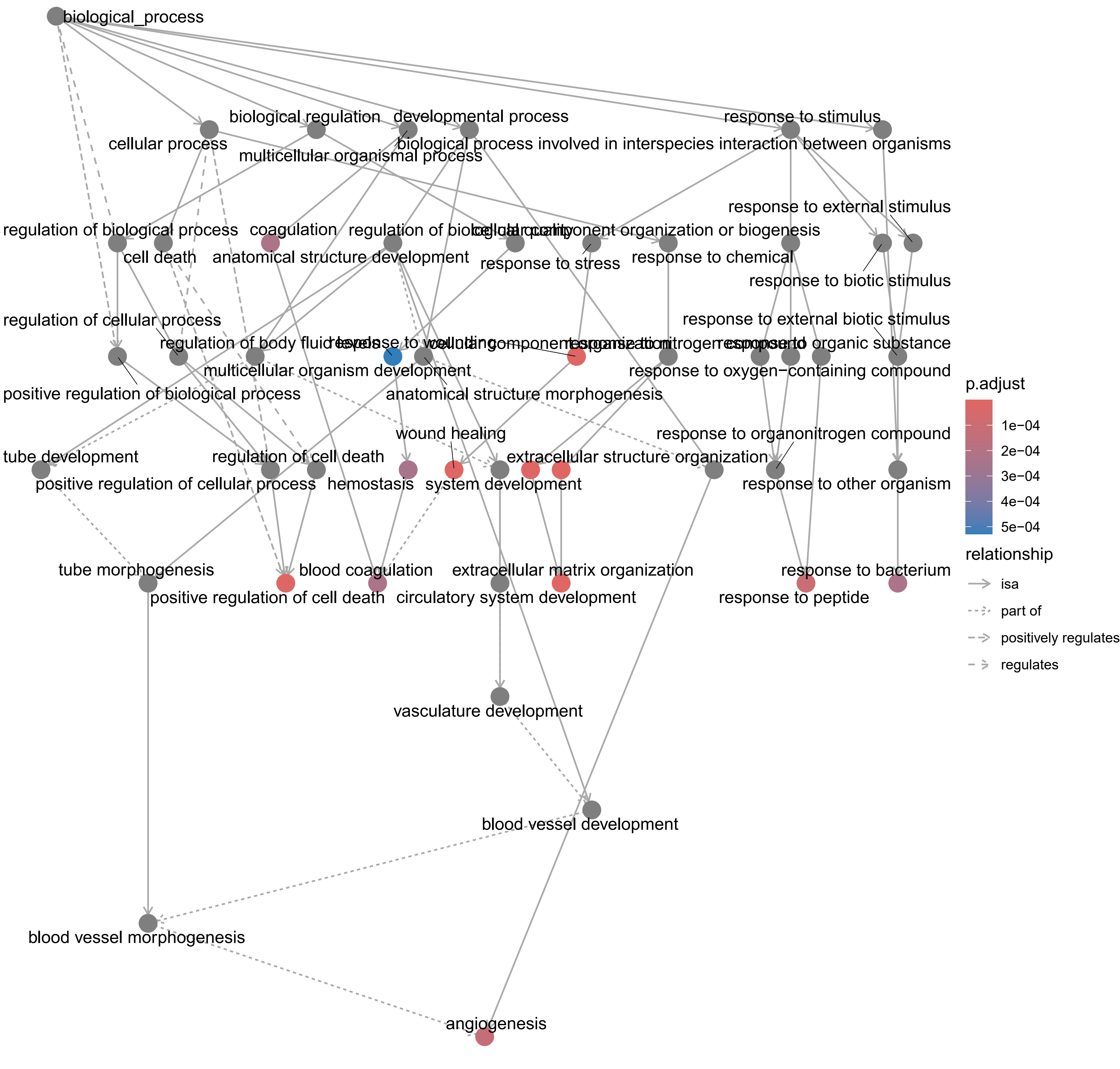

M13 Cell Component

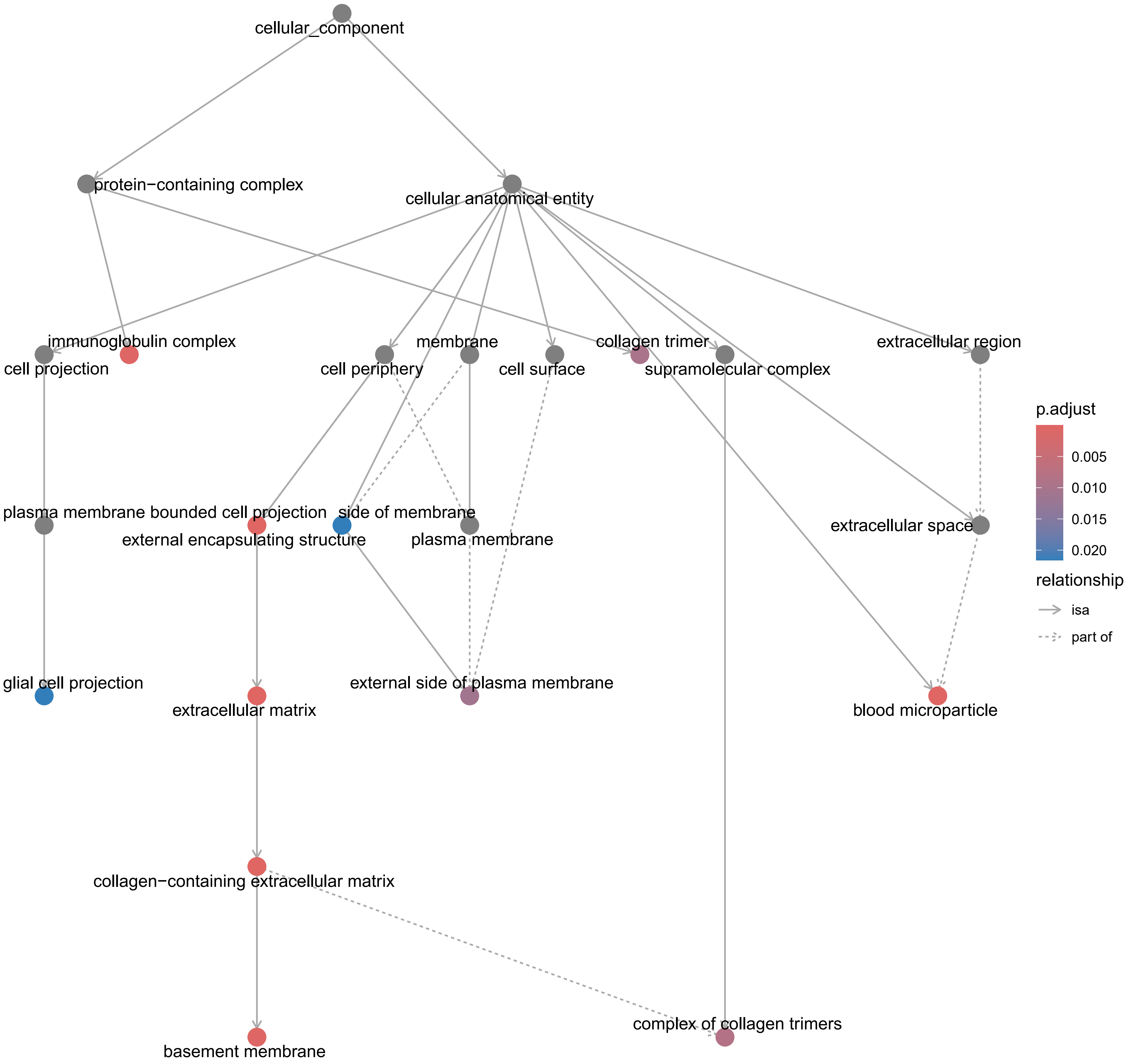

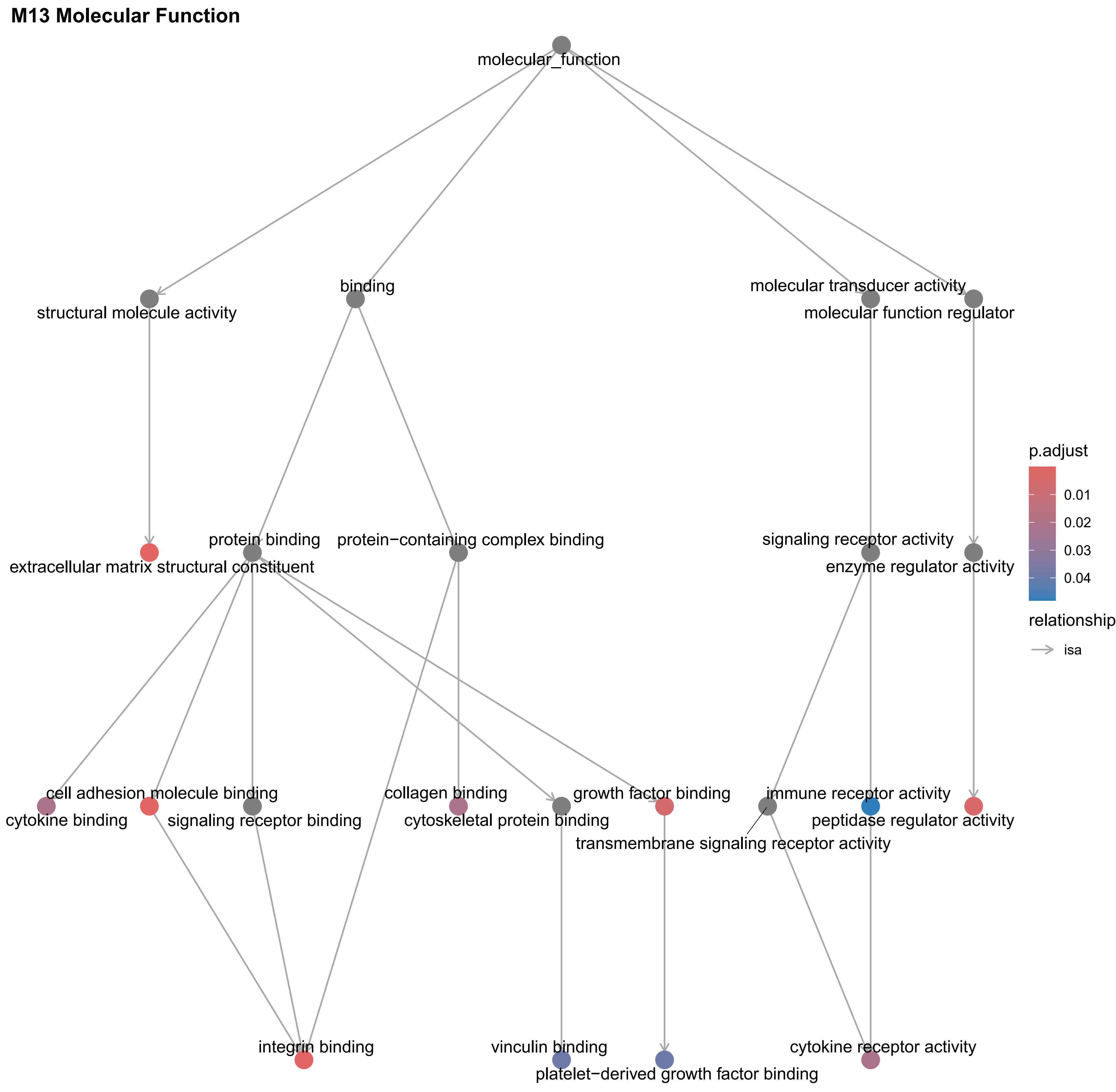
M13 Molecular Function
molecular_function
structural molecule activity
binding
molecular transducer activity
molecular function regulator
extracellular matrix structural constituent
protein binding
protein−containing complex binding
signaling receptor activity
enzyme regulator activity
cell adhesion molecule binding
cytokine binding
signaling receptor binding
collagen binding
cytoskeletal protein binding
growth factor binding
immune receptor activity
peptidase regulator activity
transmembrane signaling receptor activity
integrin binding
vinculin binding
platelet−derived growth factor binding
cytokine receptor activity
p.adjust
0.01
0.02
0.03
0.04
relationship
isa

M14 Biological Process

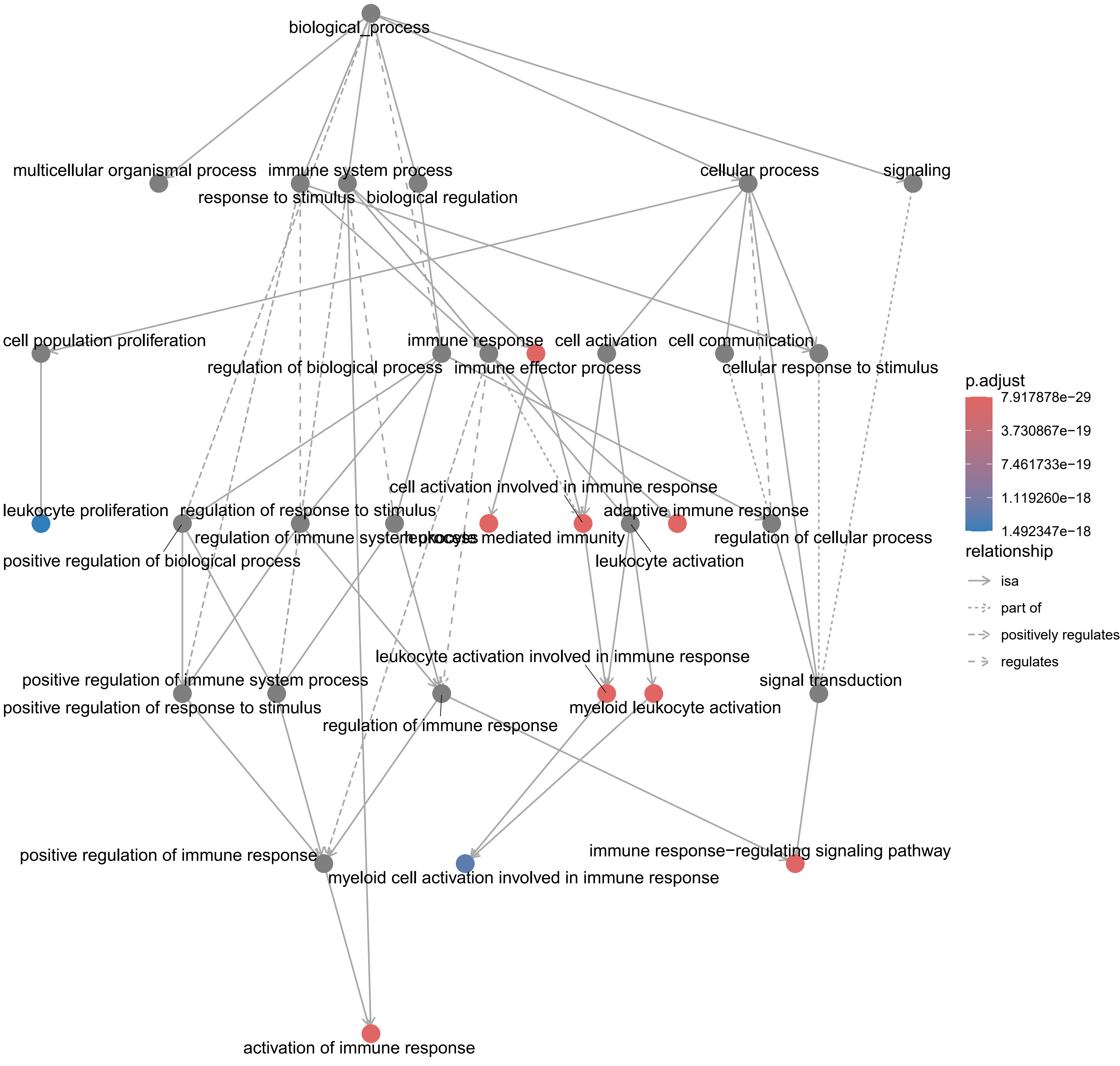

M14 Cell Component

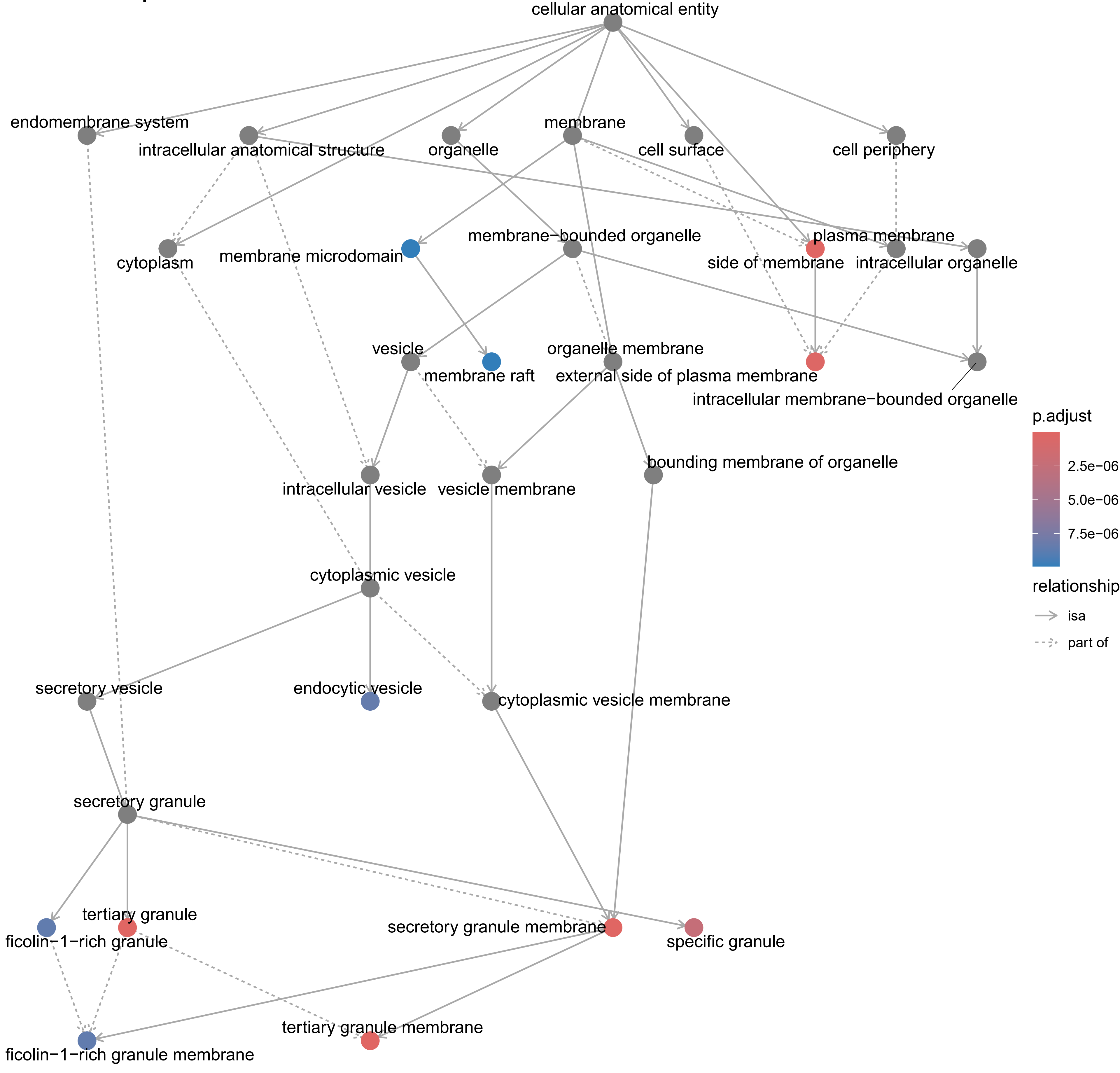

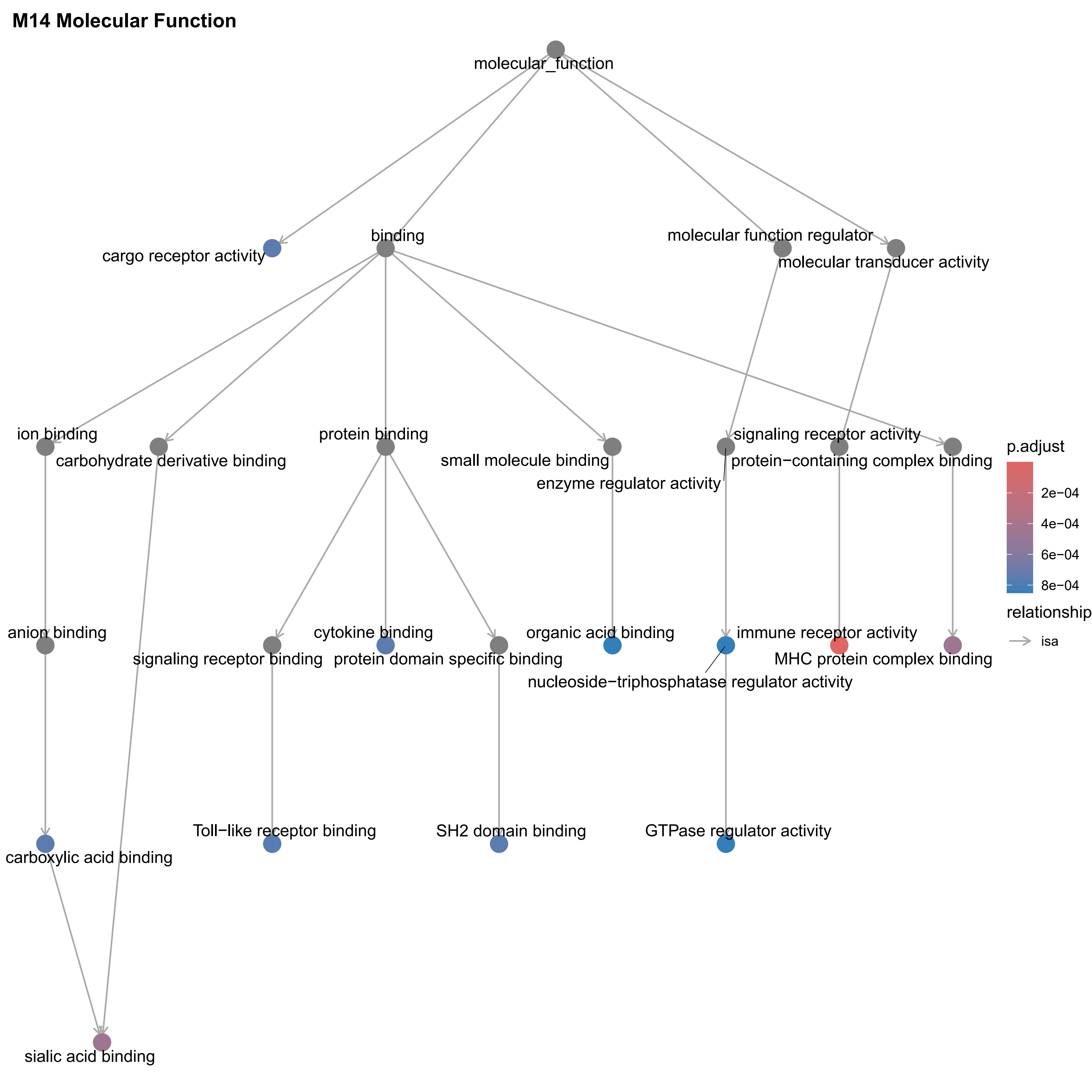
M14 Molecular Function
molecular_function
cargo receptor activity
binding
molecular function regulator
molecular transducer activity
ion binding
carbohydrate derivative binding
protein binding
small molecule binding
enzyme regulator activity
signaling receptor activity
protein−containing complex binding
anion binding
signaling receptor binding
cytokine binding
protein domain specific binding
organic acid binding
immune receptor activity
nucleoside−triphosphatase regulator activity
MHC protein complex binding
carboxylic acid binding
Toll−like receptor binding
SH2 domain binding
GTPase regulator activity
sialic acid binding
p.adjust
2e−04
4e−04
6e−04
8e−04
relationship
isa

M15 Biological Process

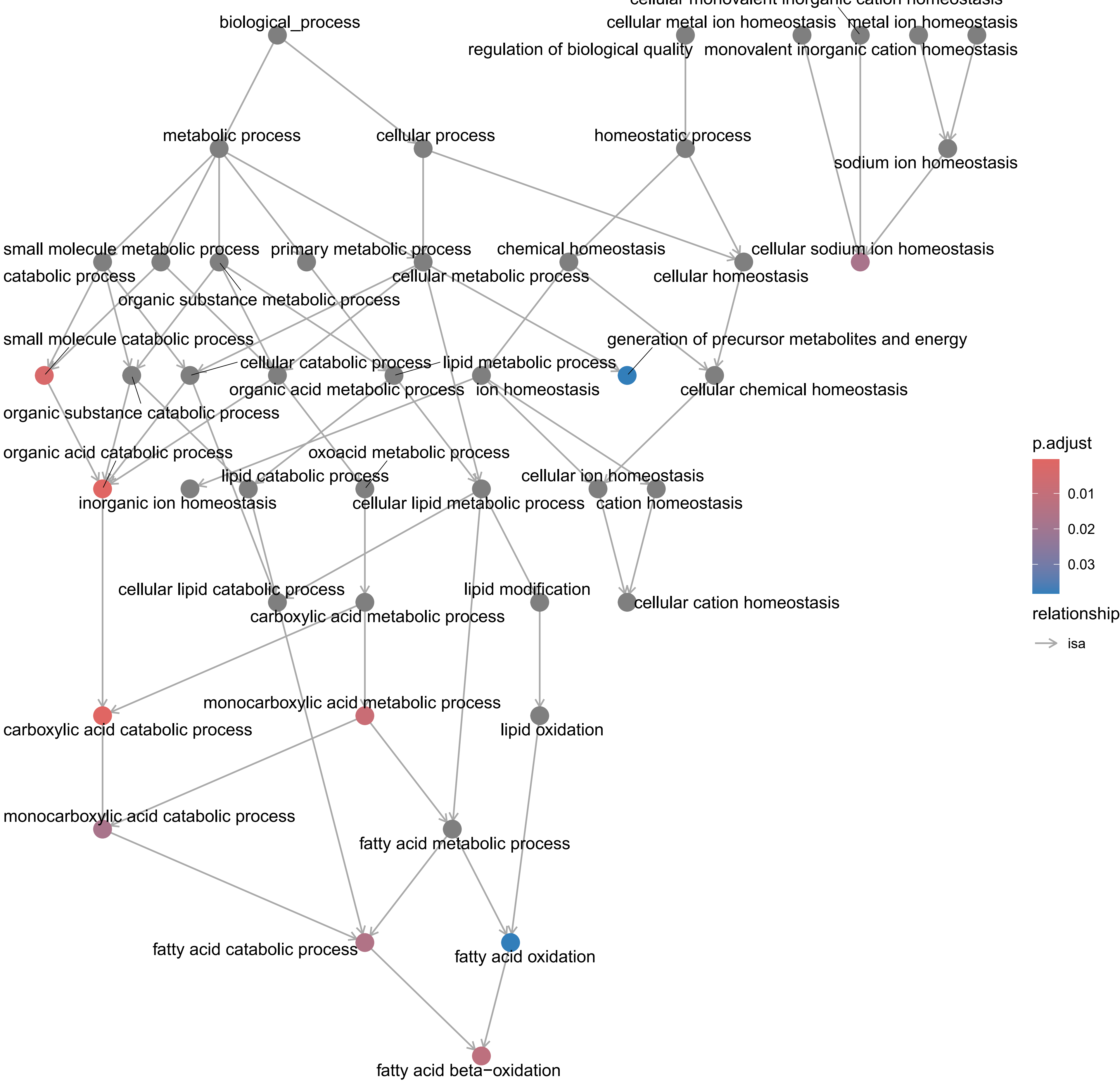

M15 Cell Component

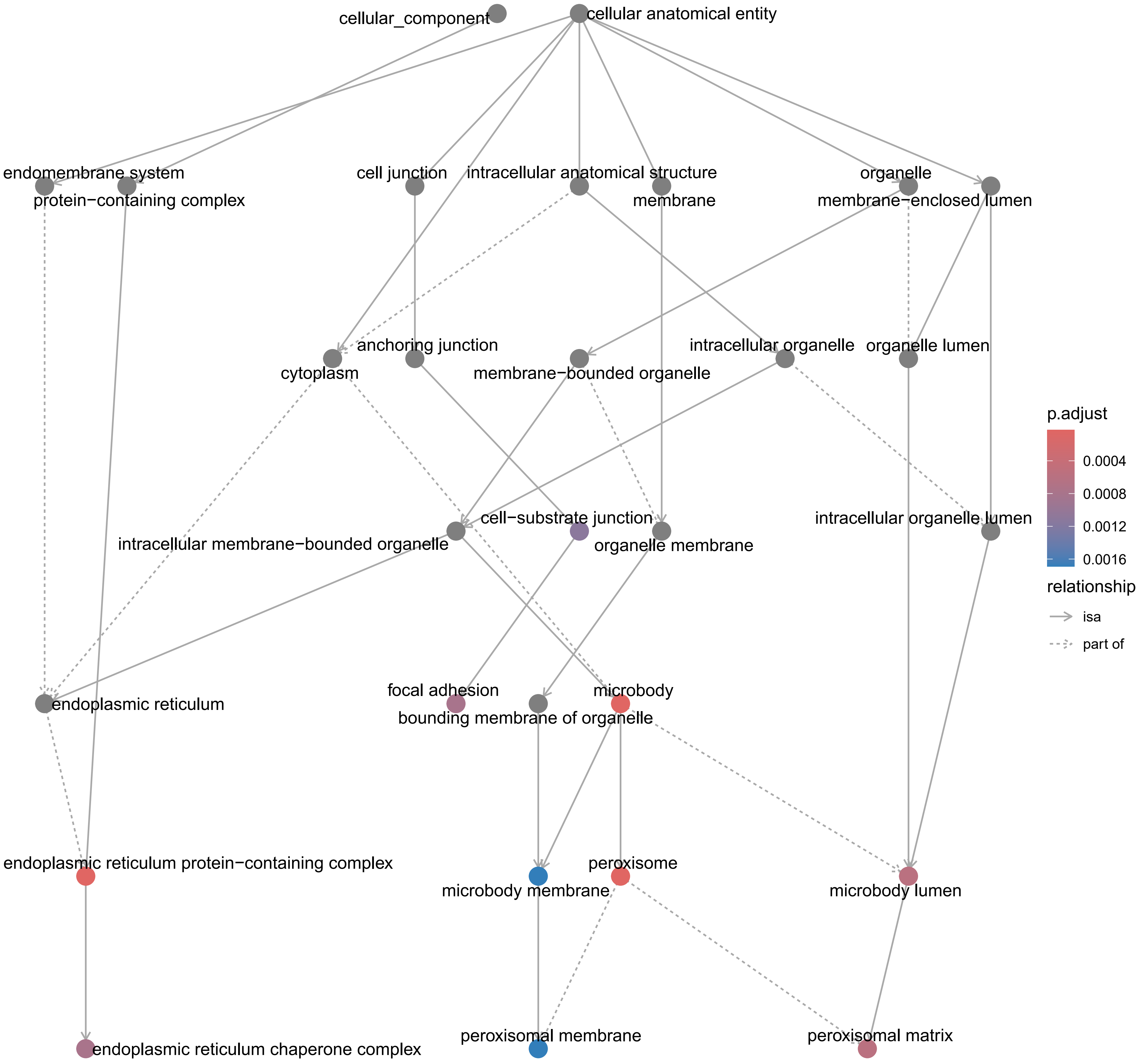

M15 Molecular Function

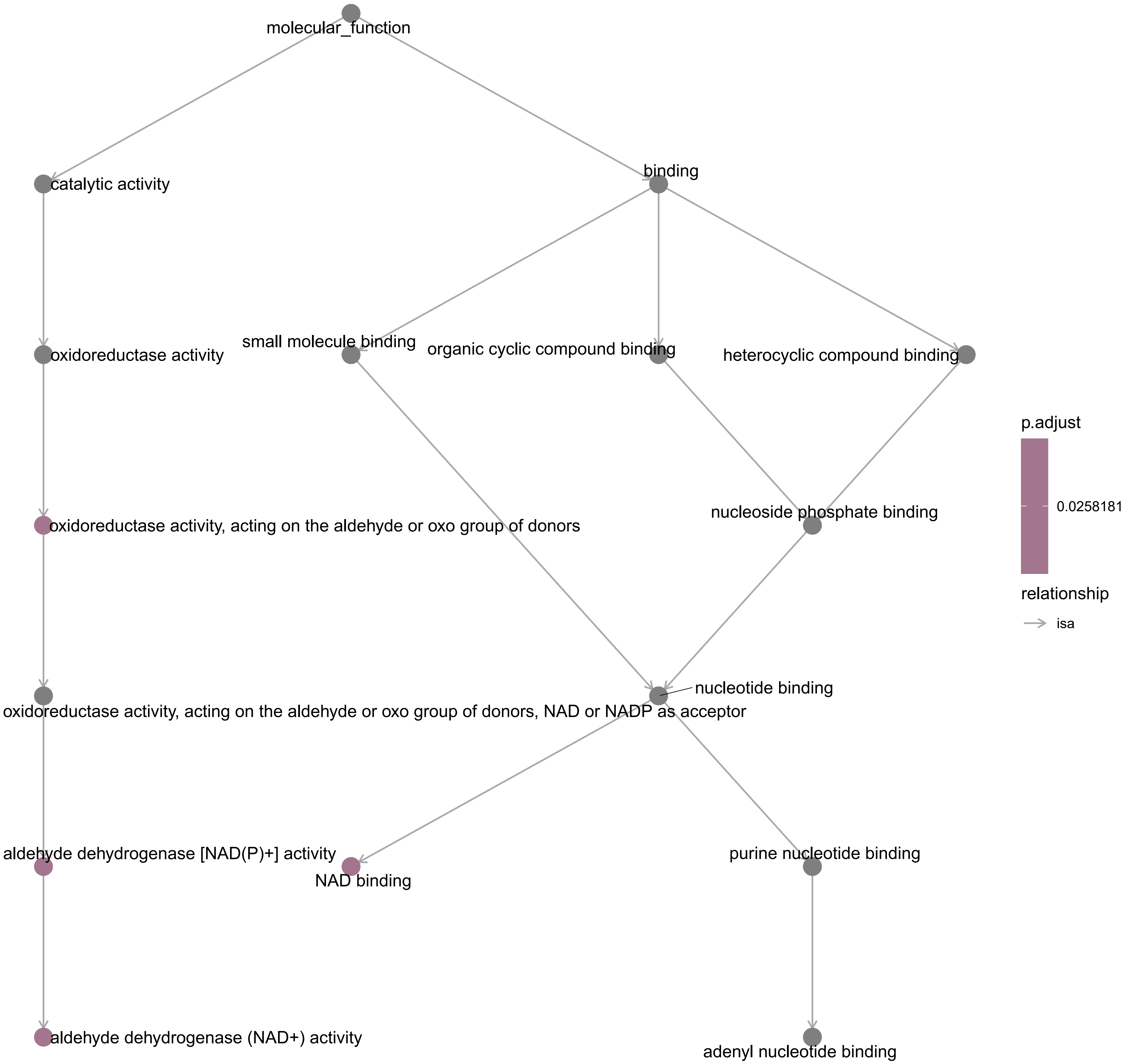

M16 Biological Process

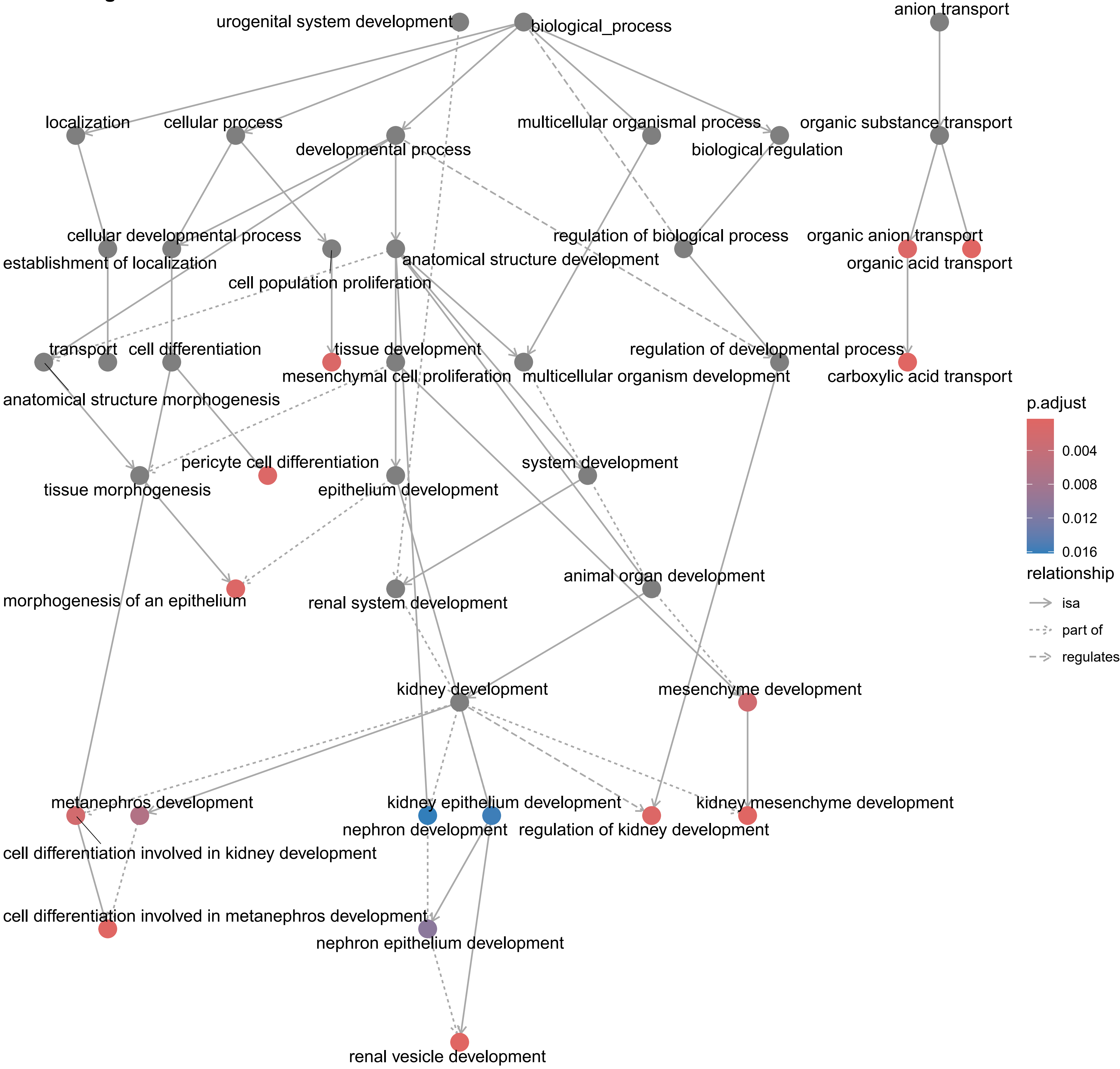

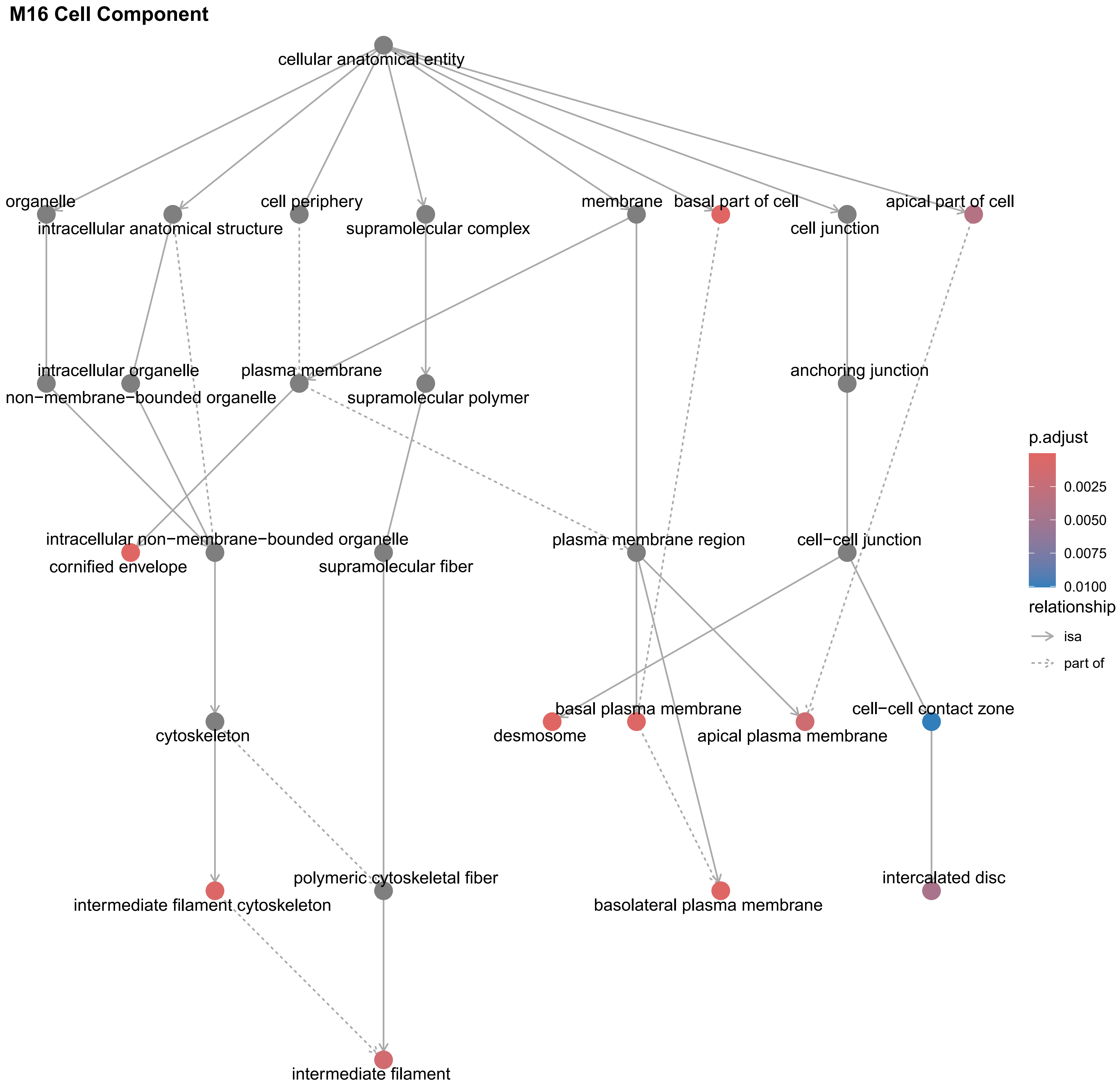
M16 Cell Component
cellular anatomical entity
organelle
intracellular anatomical structure
cell periphery
supramolecular complex
membrane
basal part of cell
cell junction
apical part of cell
intracellular organelle
non−membrane−bounded organelle
plasma membrane
supramolecular polymer
anchoring junction
intracellular non−membrane−bounded organelle
cornified envelope
supramolecular fiber
plasma membrane region
cell−cell junction
cytoskeleton
desmosome
basal plasma membrane
apical plasma membrane
cell−cell contact zone
intermediate filament cytoskeleton
polymeric cytoskeletal fiber
basolateral plasma membrane
intercalated disc
intermediate filament
p.adjust
0.0025
0.0050
0.0075
0.0100
relationship
isa
part of

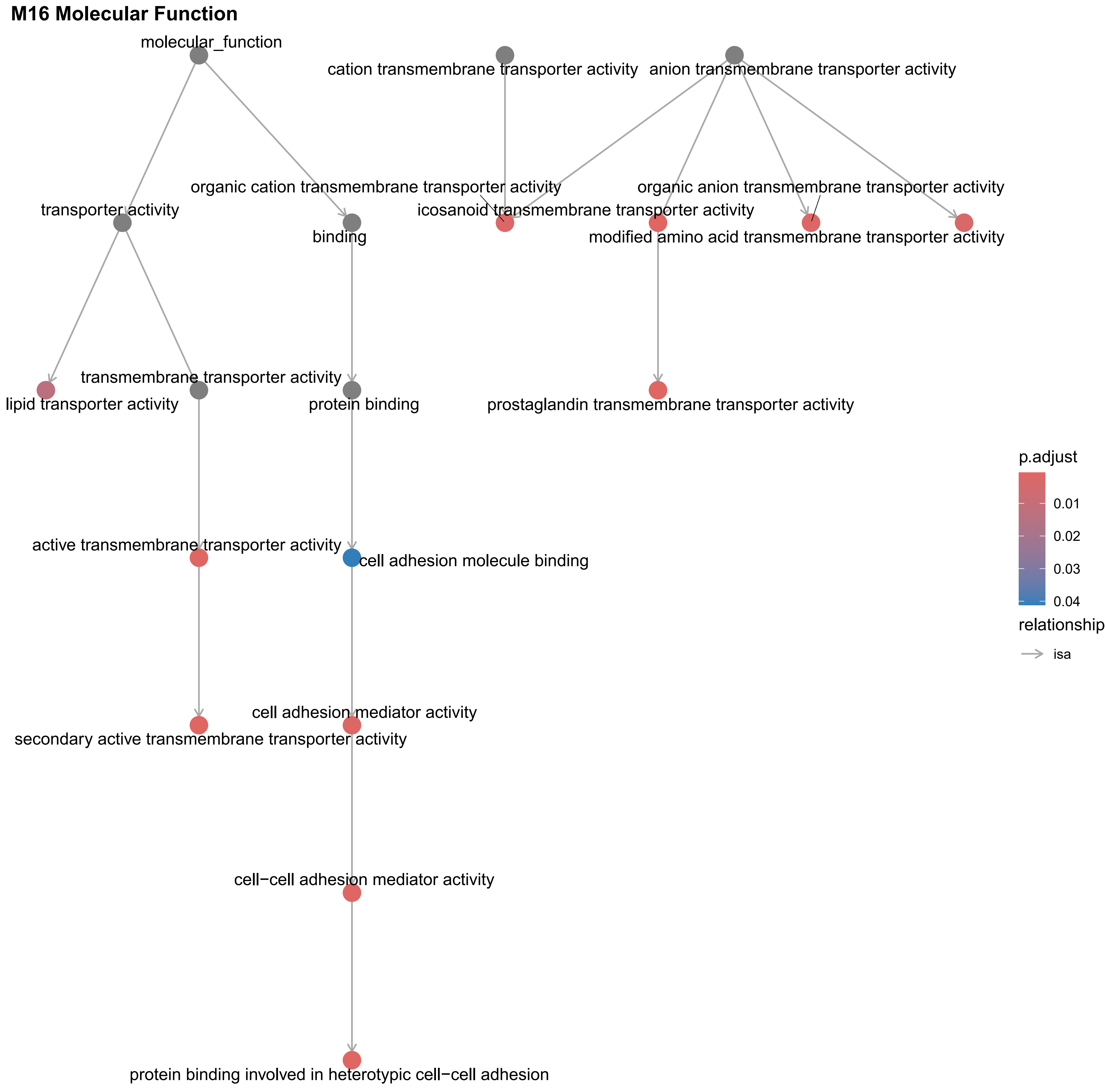

M16 Molecular Function
molecular_function
cation transmembrane transporter activity
anion transmembrane transporter activity
transporter activity
binding
organic cation transmembrane transporter activity
icosanoid transmembrane transporter activity
organic anion transmembrane transporter activity
modified amino acid transmembrane transporter activity
lipid transporter activity
transmembrane transporter activity
protein binding
prostaglandin transmembrane transporter activity
active transmembrane transporter activity
cell adhesion molecule binding
secondary active transmembrane transporter activity
cell adhesion mediator activity
cell−cell adhesion mediator activity
protein binding involved in heterotypic cell−cell adhesion
p.adjust
0.01
0.02
0.03
0.04
relationship
isa

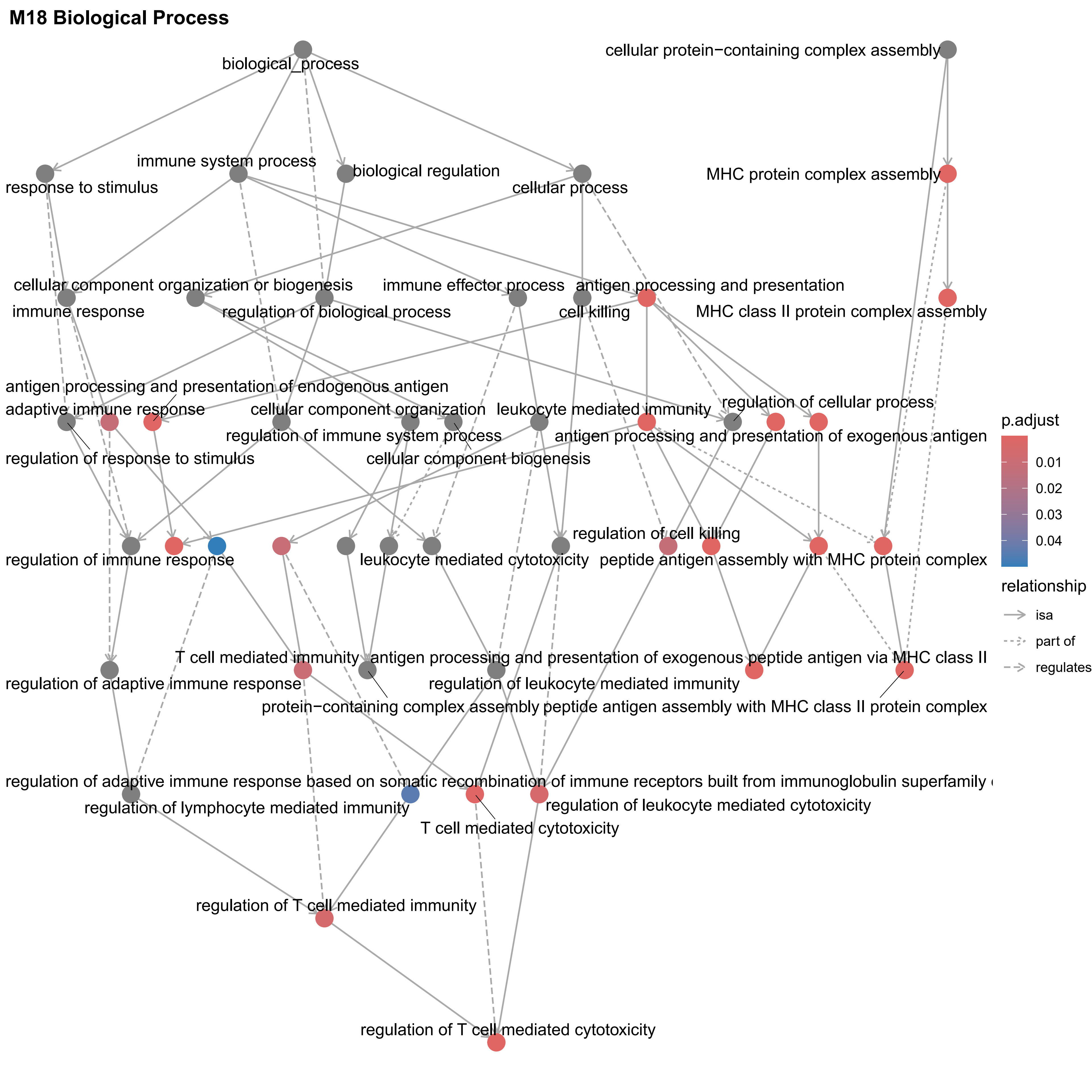
M18 Biological Process
biological_process
cellular protein−containing complex assembly
response to stimulus
immune system process
biological regulation
cellular process
MHC protein complex assembly
cellular component organization or biogenesis
immune effector process
antigen processing and presentation
immune response
regulation of biological process
cell killing
MHC class II protein complex assembly
antigen processing and presentation of endogenous antigen
adaptive immune response
cellular component organization
leukocyte mediated immunity
regulation of cellular process
regulation of immune system process
antigen processing and presentation of exogenous antigen
regulation of response to stimulus
cellular component biogenesis
regulation of cell killing
regulation of immune response
leukocyte mediated cytotoxicity
peptide antigen assembly with MHC protein complex
T cell mediated immunity
antigen processing and presentation of exogenous peptide antigen via MHC class II
regulation of adaptive immune response
regulation of leukocyte mediated immunity
protein−containing complex assembly
peptide antigen assembly with MHC class II protein complex
regulation of adaptive immune response based on somatic recombination of immune receptors built from immunoglobulin superfamily d
regulation of lymphocyte mediated immunity
regulation of leukocyte mediated cytotoxicity
T cell mediated cytotoxicity
regulation of T cell mediated immunity
regulation of T cell mediated cytotoxicity
p.adjust
0.01
0.02
0.03
0.04
relationship
isa
part of
regulates

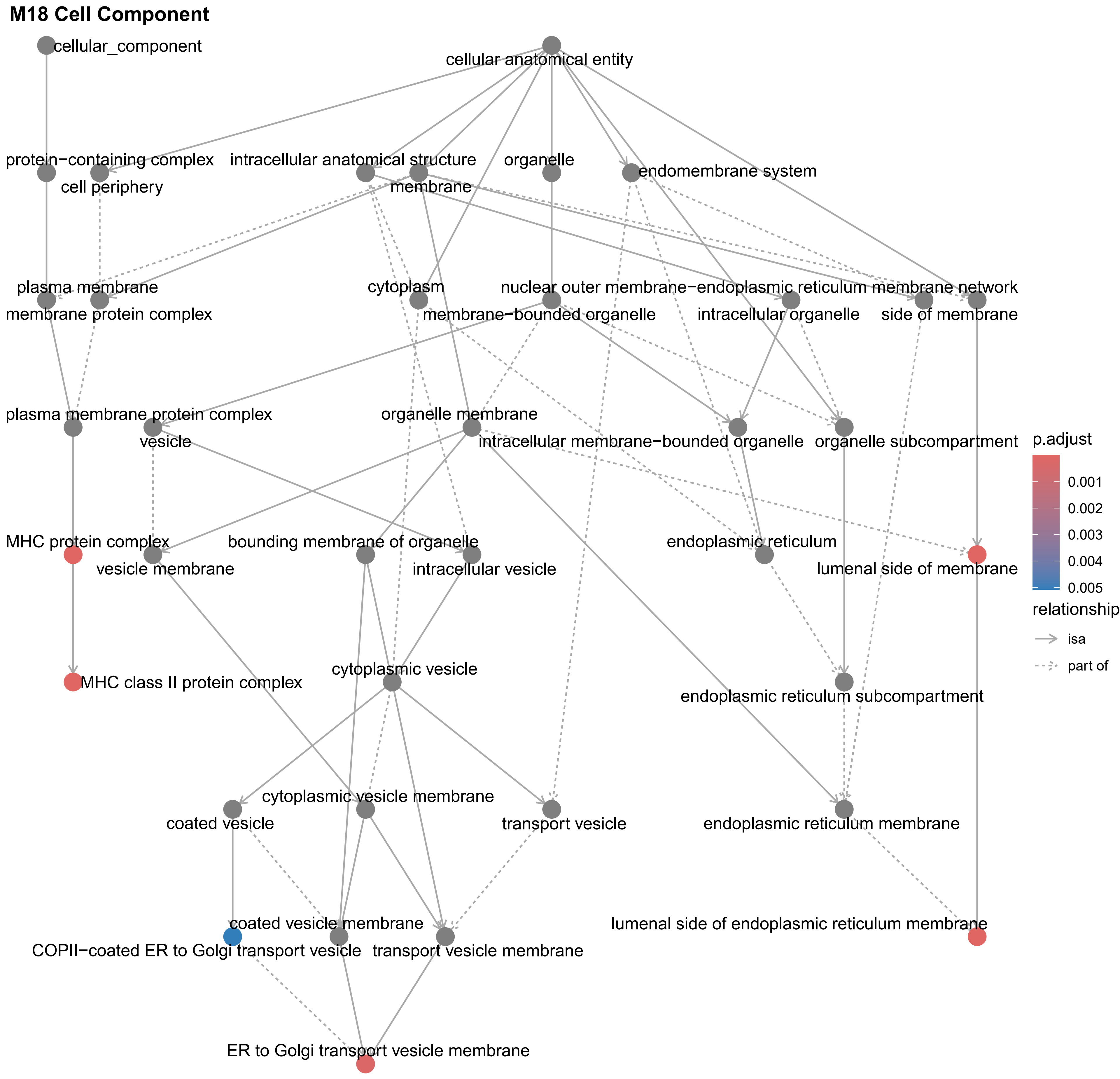
M18 Cell Component
cellular_component
cellular anatomical entity
protein−containing complex
cell periphery
intracellular anatomical structure
membrane
organelle
endomembrane system
plasma membrane
membrane protein complex
cytoplasm
nuclear outer membrane−endoplasmic reticulum membrane network
membrane−bounded organelle
intracellular organelle
side of membrane
plasma membrane protein complex
vesicle
organelle membrane
intracellular membrane−bounded organelle
organelle subcompartment
MHC protein complex
vesicle membrane
bounding membrane of organelle
intracellular vesicle
endoplasmic reticulum
lumenal side of membrane
MHC class II protein complex
cytoplasmic vesicle
endoplasmic reticulum subcompartment
cytoplasmic vesicle membrane
coated vesicle
transport vesicle
endoplasmic reticulum membrane
coated vesicle membrane
COPII−coated ER to Golgi transport vesicle
transport vesicle membrane
lumenal side of endoplasmic reticulum membrane
ER to Golgi transport vesicle membrane
p.adjust
0.001
0.002
0.003
0.004
0.005
relationship
isa
part of

M18 Molecular Function

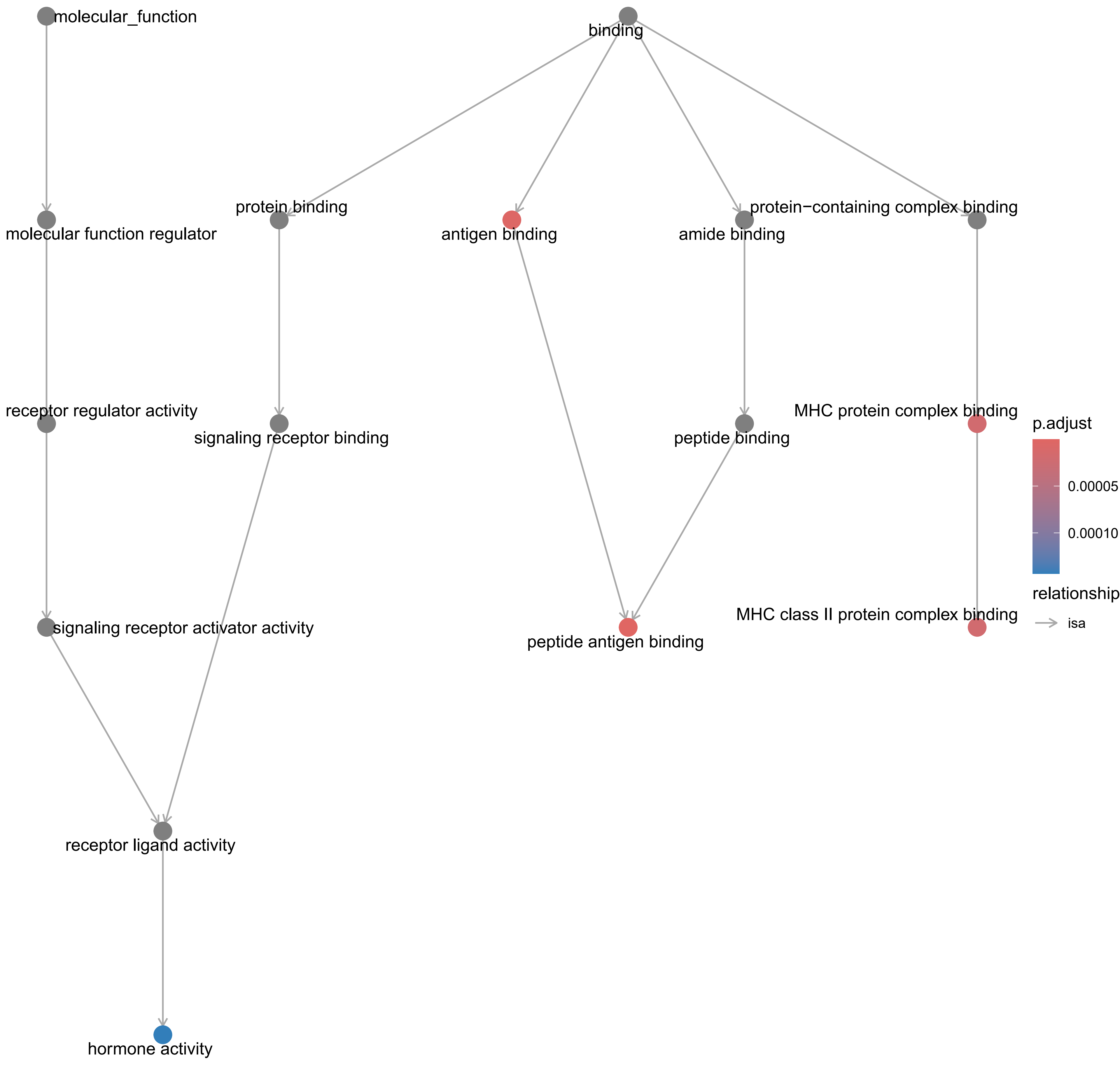

Supplement: Supplementary file 1 [file awag085_supplementary_data.zip › brain-2025-02148-File012.pdf]
